# Systematic Variation of 3d Metal Centers in a Redox-Innocent Ligand Environment: Structures, Electrochemical Properties, and Carbon Dioxide Activation

**DOI:** 10.1021/acs.inorgchem.1c02909

**Published:** 2021-12-01

**Authors:** Niklas W. Kinzel, Derya Demirbas, Eckhard Bill, Thomas Weyhermüller, Christophe Werlé, Nicolas Kaeffer, Walter Leitner

**Affiliations:** † Max Planck Institute for Chemical Energy Conversion, Stiftstrasse 34−36, 45470 Mülheim an der Ruhr, Germany; ‡ Institut für Technische und Makromolekulare Chemie, RWTH Aachen University, Worringer Weg 2, 52074 Aachen, Germany; § Ruhr University Bochum, Universitätsstrasse 150, 44801 Bochum, Germany; ⊥ Max-Planck-Institut für Kohlenforschung, Kaiser-Wilhelm-Platz 1, 45470 Mülheim an der Ruhr, Germany

## Abstract

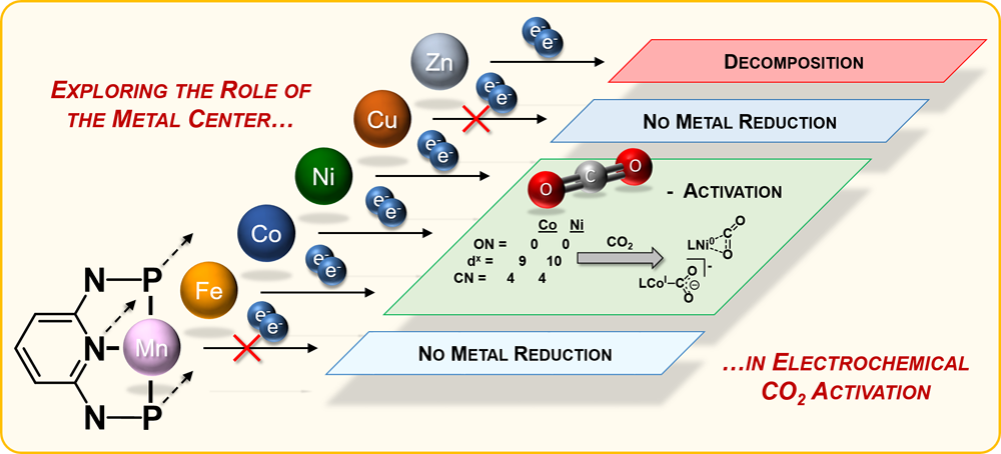

Coordination compounds of earth-abundant 3d transition metals are among the most effective catalysts for the electrochemical reduction of carbon dioxide (CO_2_). While the properties of the metal center are crucial for the ability of the complexes to electrochemically activate CO_2_, systematic variations of the metal within an identical, redox-innocent ligand backbone remain insufficiently investigated. Here, we report on the synthesis, structural and spectroscopic characterization, and electrochemical investigation of a series of 3d transition-metal complexes [M = Mn(I), Fe(II), Co(II), Ni(II), Cu(I), and Zn(II)] coordinated by a new redox-innocent PNP pincer ligand system. Only the Fe, Co, and Ni complexes reveal distinct metal-centered electrochemical reductions from M(II) down to M(0) and show indications for interaction with CO_2_ in their reduced states. The Ni(0) d^10^ species associates with CO_2_ to form a putative Aresta-type Ni-η^2^-CO_2_ complex, where electron transfer to CO_2_ through back-bonding is insufficient to enable electrocatalytic activity. By contrast, the Co(0) d^9^ intermediate binding CO_2_ can undergo additional electron uptake into a formal cobalt(I) metallacarboxylate complex able to promote turnover. Our data, together with the few literature precedents, single out that an unsaturated coordination sphere (coordination number = 4 or 5) and a d^7^-to-d^9^ configuration in the reduced low oxidation state (+I or 0) are characteristics that foster electrochemical CO_2_ activation for complexes based on redox-innocent ligands.

## Abstract 2

A series of 3d transition-metal complexes (M = Mn, Fe, Co, Ni, Cu, and Zn) coordinated by a new redox-innocent PNP pincer ligand system were synthesized and structurally as well as electrochemically analyzed to illuminate the role of the metal center in molecular electrochemical carbon dioxide (CO_2_) activation.

## 1 Introduction

**Figure 1:**
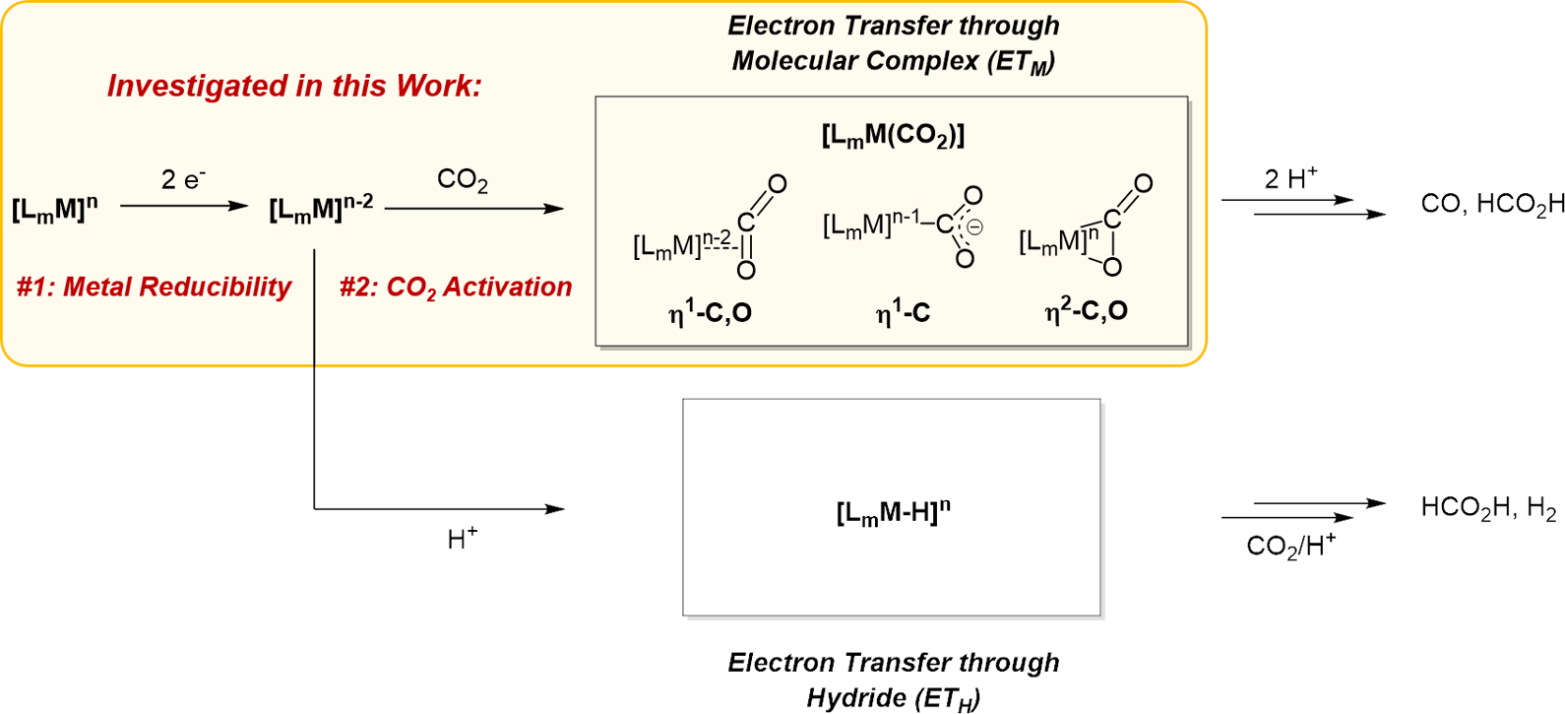
Electron transfers during transition-metal-catalyzed electroreduction of CO_2_: ET_M_ and ET_H_ pathways (M = metal, L = ligand, m = stoichiometry of coordinated ligands, and n = formal oxidation state of the metal).

Closing the anthropogenic carbon cycle constitutes a pivotal challenge to tackle climate change and provide humankind with the necessary carbon-based materials in a defossilized future.^1^ In this context, the catalytic conversion of carbon dioxide either taken from industrial waste streams or ultimately drawn from the atmosphere poses a powerful tool.^2,3^ Turning the chemically inert CO_2_ molecule into a C_1_ building block accessible for further transformation (e.g., copolymerization,^4−6^ hydrogenation,^7−9^ electro-/photochemical reduction^10,11^ or synthesis,^12,13^ and combinations thereof^14,15^) is a promising strategy toward that aim. The thermodynamic challenge of this approach can be virtuously addressed in supplying the required energy for the conversion of CO_2_ by renewable electricity. To overcome the kinetic barriers, molecular complexes of earth-abundant 3d transition metals are heavily investigated, ranging among the most effective and efficient electrochemical CO_2_ reduction (eCO_2_R) catalysts.^10,16^ In a recent literature survey, we analyzed the main reaction pathways of homogeneously catalyzed CO_2_ electroreduction from an organometallic perspective. We classified them into mechanisms traversing the direct coordination and activation of CO_2_ to the metal center (electron transfer through a molecular complex, ET_M_) and those requiring the previous formation of a metal hydride (electron transfer through hydride, ET_H_) as the reactive intermediate (Figure 1).^17^

**Figure 2:**
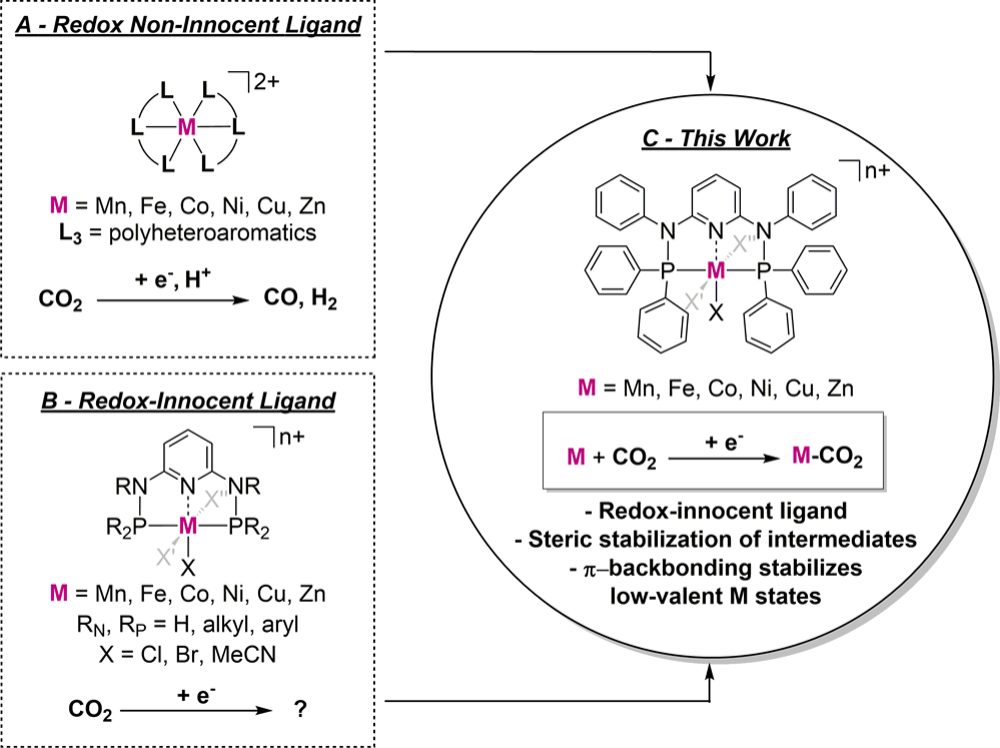
(A) Studies investigating 3d metal series in the same noninnocent ligand frameworks,^25,27^ (B) complexes reported in the literature coordinated by redox-innocent PN_3_P ligand frameworks,^32−38^ and (C) focus of this work.

The intrinsic characteristics of the metal center, such as hydricity^18−22^ or substrate (CO_2_) and product (CO, HCO_2_H, etc.) binding affinity,^23^ define the prevailing mechanistic route, which is directly linked to the catalytic performance. However, only a limited number of studies report on the systematic variation of the metal center within an identical ligand framework in relation to CO_2_ electroreduction (Figure 2A).^24−27^ The majority of these studies rely on the so-called “noninnocent” ligands because these ligands are often perceived as beneficial for the activity by sharing excess electron density (redox noninnocence) or relaying protons (chemical noninnocence).^28−30^ The resulting complex interplay of ligand- and metal-centered processes renders deconvolution of the individual contributions rather challenging.^31^ By contrast, systematic variations of the metal centers with redox-innocent ligands, such as pincer platforms,^32−39^ remain insufficiently investigated in the frame of CO_2_ electroreduction (Figure 2B).

**Figure 3:**
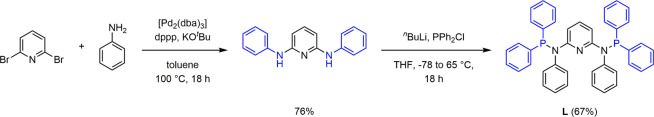
Synthesis of **L**.

In the present study, we thus investigated the structural and electrochemical properties toward CO_2_ conversion of 3d transition-metal complexes based on an identical “innocent” ligand framework. We purposely aimed at involving a ligand inert to redox and protonation processes under common electrocatalytic conditions (Figure 2C) and therefore designed the new PNP ligand **L** (Figure 3). The ligand displays a large steric demand to shield the metal center and a high degree of aromaticity, which we surmised would only marginally interfere in the redox processes but still provide sufficient π-back-bonding ability to stabilize low-valent metals. We report the synthesis, structural, and spectroscopic characterizations of a series of complexes comprising mid-to-late 3d transition metals (Mn to Zn) coordinated by this pincer-type ligand platform. The redox properties of the complexes were then studied, employing cyclic voltammetry (CV) to probe the reducibility of the core metal (requisite #1 in Figure 1). We then investigated the most promising set of complexes (Fe, Co, and Ni) toward electrochemical activation of the CO_2_ substrate (requisite #2 in Figure 1). We specifically focus this study on the metal–CO_2_ interaction as the entry into an electrocatalytic CO_2_ reduction cycle along the ET_M_ pathway (highlighted in Figure 1) by depriving the system of protons to hinder the ET_H_ route.

## 2 Results

### 2.1 Synthesis and Structural Characterization

#### 2.1.1 *N*^2^,*N*^6^-Bis(diphenylphosphanyl)-*N*^2^,*N*^6^-diphenylpyridine-2,6-diamine (**L**)

Ligand **L** was prepared following a two-step synthesis route (Figure 3).

In the first step, 2,6-dibromopyridine and aniline were reacted following a reported palladium-catalyzed Buchwald–Hartwig coupling to produce *N*^2^,*N*^6^-diphenylpyridine-2,6-diamine,^40^ which was isolated in 76% yield after purification by column chromatography on silica. Subsequently, diphenylphosphino moieties were installed by low-temperature lithiation of the diamine in tetrahydrofuran (THF) before the addition of chlorodiphenylphosphine and stirring at 65 °C for 18 h. NMR spectroscopic analysis (Figures S1–S3), high-resolution mass spectrometry (HRMS), and elemental analysis confirmed the structure and purity of ligand **L** received in 67% yield after workup (detailed synthetic procedures are given in the Experimental Section).

**Figure 4:**
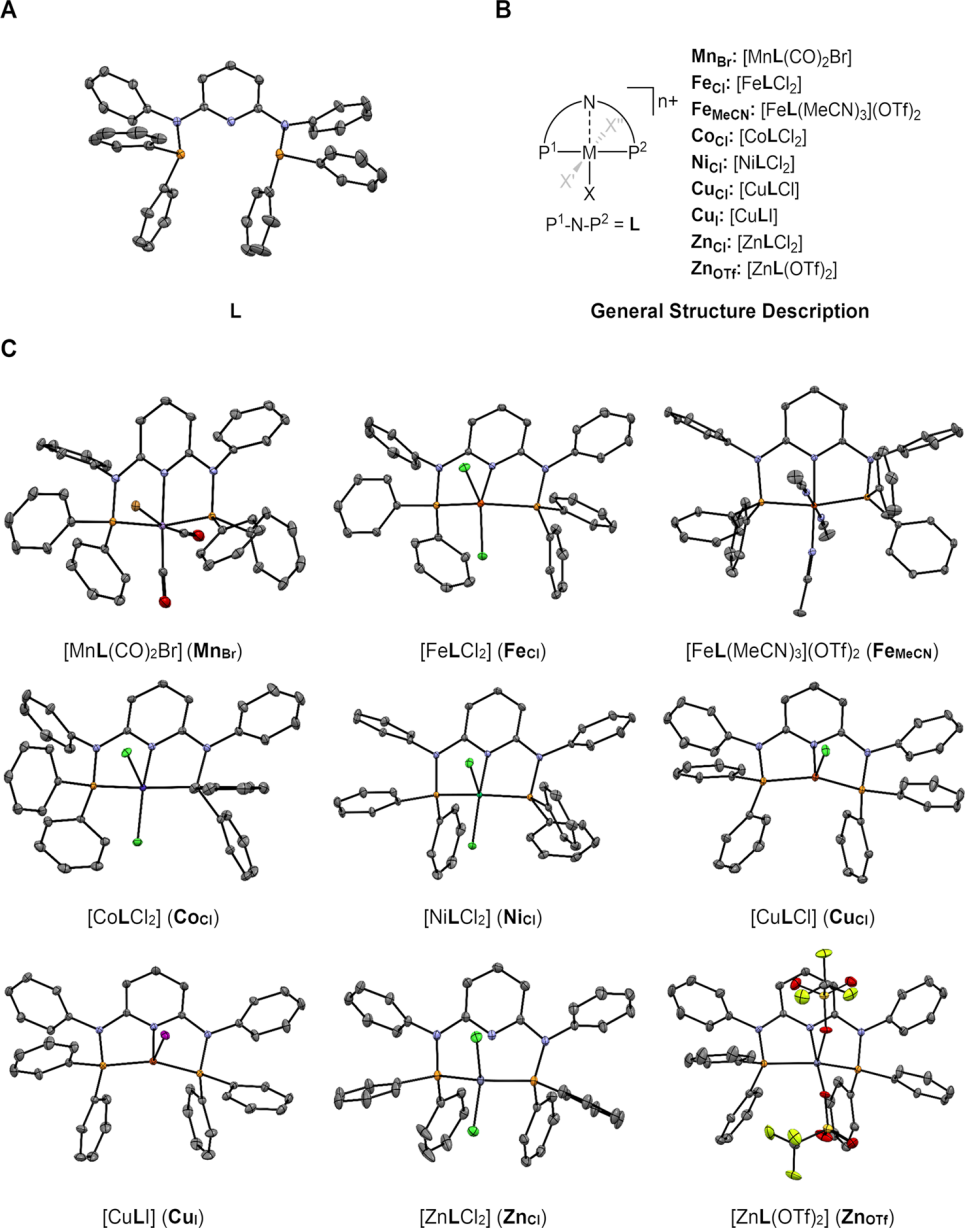
(A) Molecular structure of **L**, (B) general structure description, and (C) molecular structures of the complexes **M**_**X**_ investigated in this study. H atoms, outer-sphere ligands, and cocrystallized solvent molecules were omitted for clarity; thermal ellipsoids are shown at the 50% probability level. For **Mn**_**Br**_, the major *cis*-CO configuration is presented. Color code: gray, C; blue, N; red, O; yellow, F; orange, P; green, Cl; brown, Br; pink, I.

Even before the coordination of a metal atom, the molecular structure of the ligand (Figure 4A) exhibits the typical pincer shape, with the five atoms forming the PN_3_P pincer belt nearly coplanar (PNCN torsion angles at 8.0° and −15.4°). This planarity might result from a steric hindering of the rotation around the C–N bond linking the pyridine core and the bulky aniline moiety or from conjugation of the lone pairs of the aniline N atoms with the heterocycle, inferring in-plane triangular geometry at these N atoms. We also note that the *pinching* character of **L** is marked with a relatively short P–P distance (*d*_P–P_ = 4.226 Å), likely by virtue of the steric constraint imposed at the aniline N atoms, making the C–N–P angles narrow (119.5° and 115.7°).

Pincer transition-metal complexes were obtained by the metalation of **L** with 3d transition-metal precursors in yields ranging from 52 to 95%, as indicated in the Experimental Section. Schematic structures of the resulting **M**_**X**_ complexes (where M = metal center and X = inner-sphere coordinating unit regardless of the stoichiometry) are presented in Figure 4B.

For a systematic comparison, we first targeted pincer complexes bearing metal centers in their +II oxidation state and bis-chloride coordination. These structures were successfully obtained for Fe (**Fe**_**Cl**_), Co (**Co**_**Cl**_), Ni (**Ni**_**Cl**_), and Zn (**Zn**_**Cl**_) but remained elusive for Mn and Cu (*vide infra*). In these cases, complexes **Mn**_**Br**_ and **Cu**_**Cl**_ were derived from the Mn(CO)_5_Br and CuCl precursors, respectively, in their +I oxidation state. Along with these complexes, we also made variations to less electronegative or more weakly coordinating anions and prepared **Fe**_**MeCN**_, **Cu**_**I**_, and **Zn**_**OTf**_.

Single crystals could be obtained for **L** and each of the **M**_**X**_ complexes, allowing elucidation of their molecular structures by X-ray diffraction (XRD), as represented in Figure 4C.

Characteristic features of the structures (Figure 4C), calculated structural parameters and coordination geometries (Table 1), and selected NMR spectroscopic data (Table 2) will be discussed for each species individually in the following sections.

**Table 1:** Selected Bond Distances (Å) and Angles (deg) as well as Structural Parameters τ and Idealized Coordination Geometries for the Complexes in this Study

	M–N	M–P^1^	M–P^2^	P^1^–M–N	P^1^–M–P^2^	τ_4_	τ_5_^41^	idealized geometry
**Mn**_**Br**_	2.0391(13)	2.2623(5)	2.2273(5)	83.22(4)	166.305(18)			*O*_*h*_
**Fe**_**Cl**_	2.3269(10)	2.4260(3)	2.4291(3)	73.77(2)	130.409(13)		0.38	SBP
**Fe**_**MeCN**_	1.9717(9)	2.2411(3)	2.2238(3)	83.67(3)	167.824(12)			*O*_*h*_
**Co**_**Cl**_	1.9465(10)	2.1882(4)	2.1847(4)	84.90(3)	166.277(14)		0.04	SBP
**Ni**_**Cl**_	1.9084(11)	2.1407(4)	2.1574(4)	85.26(3)	155.509(15)		0.23	SBP
**Cu**_**Cl**_	2.1377(8)	2.2556(3)	2.2687(3)	80.89(2)	135.982(11)	0.78		*T*_*d*_
**Cu**_**I**_	2.1283(7)	2.2299(2)	2.2589(2)	78.69(19)	133.215(9)	0.77		*T*_*d*_
**Zn**_**Cl**_	2.7383(9)^a^	2.4489(3)	2.4182(3)	64.50(2)^a^	112.597(11)	0.95		*T*_*d*_
**Zn**_**OTf**_	2.335(2)	2.3979(8)	2.3758(8)	72.38(6)	129.09(3)		0.82	TBP

a No bond between the metal and N.

**Table 2:** **^31^**P NMR (δ_P_) and Coordination (Δδ) Chemical Shifts, ^1^H NMR Chemical Shifts of the Pyridinic Protons in the Meta Position [δ_H_(Py*H*_m_); CD_2_Cl_2_, 500 MHz, 296 K] for Complexes in this Study, and Spin *S* for Paramagnetic Complexes in this Study

	δ_P_ (ppm)	Δδ (ppm)	δ_H_(Py*H*_m_) (ppm)^a^	*S*
**L**	52.8		5.81 (8.0)	
**Mn**_**Br**_	138.9	86.1	5.79 (8.2)	
**Fe**_**Cl**_			57.14 (−)	2
**Fe**_**MeCN**_	129.2	76.4	5.99 (8.2)	
**Co**_**Cl**_				^1^/_2_
**Ni**_**Cl**_	85.0	32.2	5.73 (8.2)	
**Cu**_**Cl**_	39.9	–12.9	5.68 (8.0)	
**Cu**_**I**_	40.0	–12.8	5.67 (8.0)	
**Zn**_**Cl**_	30.9	–21.9	5.85 (8.1)	
**Zn**_**OTf**_	28.8	–24.0	5.94 (8.2)	

a ^3^*J*_doublet_ (Hz) indicated in parentheses.

#### 2.1.2 [Mn**L**(CO)_2_Br]

In the case of Mn, metalation with MnCl_2_ at room temperature (rt) in THF, as reported for related pyridine-based PNP pincers (R_N_ = H; R_P_ = ^*i*^Pr),^37^ did not succeed with our ligand nor did other variations in the conditions (solvents, temperatures, precursors, etc.). The only reported [Mn(PN_3_P)Cl_2_] structure within a pyridine-based pincer framework displays an almost ideal square-based-pyramidal (SBP) geometry (τ_5_ = 0.04) with two P atoms in the trans position of the basal plane and relatively distant from Mn (*d*_Mn–P_ = 2.574 and 2.590 Å and *d*_P–P_ = 4.886 Å), in a high-spin, five-unpaired-electron configuration (μ_eff_ ≈ 6).^37^ We surmise that **L** does not allow such significant elongation between the two P atoms, likely required in a putative [Mn**L**Cl_2_] complex. The electron-withdrawing character of the phenyl substituent at the P atom may also disfavor the coordination of a Mn(II) fragment.

By contrast, coordination of the Mn^I^(CO)_5_Br precursor effectively yields the Mn(I) complex **Mn**_**Br**_, suggesting stabilization of the lower oxidation state by **L**. This 18-electron Mn complex crystallizes in a distorted octahedral (*O*_*h*_) coordination environment, as is commonly encountered for related pincer manganese(I) carbonyl bromide complexes.^42^ In this particular case, XRD analysis revealed a mixture of the cis and trans configurations of the CO ligands in a 95:5 ratio in the crystal.

The diamagnetic d^6^ low-spin **Mn**_**Br**_ complex exhibits distinguishable, but overlapping sets of ^1^H NMR signals for the chemically inequivalent aromatic protons of the R_N_ and R_P_ phenyl moieties positioned syn or anti of Br in the predominant *cis*-CO configuration (Figure S4).

#### 2.1.3 [Fe**L**Cl_2_]

**Fe**_**Cl**_ crystallizes in a strongly distorted SBP structure, as reflected by a τ_5_ value of 0.38. The PNP coordination sites of **L** exhibit relatively extended bond distances of 2.327 Å for Fe–N as well as 2.429 and 2.426 Å for both Fe–P bonds. Interestingly, the steric demand of the R_N_ = Ph group in **L** seems to prevent the formation of bis-PN_3_P-coordinated species, which readily occurs for R_N_ = H.^33^

Although ^1^H NMR analysis of **Fe**_**Cl**_ in CD_2_Cl_2_ exhibits the contact-shifted peaks diagnostic of a paramagnetic Fe(II) center, signals at 57.50 and −11.79 ppm are assigned by integration to pyridinic protons in the meta and para positions, respectively, and indicate a high level of symmetry in the structure (Figure S7).

The zero-field ^57^Fe Mössbauer spectrum of **Fe**_**Cl**_ recorded with a powder sample at 80 K showed a quadrupole doublet with high isomer shift, δ = 0.80 mm·s^–1^, and large quadrupole splitting, Δ*E*_Q_ = 3.33 mm·s^–1^ (Figure S32). The values are typical of high-spin Fe(II) with *S* = 2. Accordingly, the compound was electron paramagnetic resonance (EPR)-silent in a THF solution at X-band frequencies [because the zero-field splitting of the quintet state exceeds the microwave quantum energy, as is often encountered for the 3d^6^ configuration of Fe(II)]. Moreover, the effective magnetic moment of solid **Fe**_**Cl**_ was μ_eff_ = 4.9 μ_B_ at 270 K (Figure S35), in agreement with the spin-only value expected for *S* = 2. The axial zero-field splitting parameter obtained from the temperature variation of the magnetic susceptibility χ*T* versus temperature *T* is in the usual range for Fe(II), *S* = 2 with *D* = 4 cm^–1^.

In the 3d^6^ high-spin electronic configuration of Fe(II) in **Fe**_**Cl**_, the highest occupied molecular orbital (HOMO) is a d_*x*^2^–*y*^2^_ one, which is expected to be stabilized by distortion of the SBP structure to high θ_apic_ angle values (up to 117°).^43^ Similar distorted SBP geometries were observed on a series of related PN_3_P-coordinated iron bis-chloride complexes, for which extended Fe–N and Fe–P distances were related to a high-spin state at Fe.^34^

#### 2.1.4 [Fe**L**(MeCN)_3_]Cl_2_ and [Fe**L**(MeCN)_3_](OTf)_2_

Dissolving **Fe**_**Cl**_ in acetonitrile (MeCN; e.g., for CV analysis) results in a color change from yellow to red (for UV/vis spectra see Figure S31), indicative of a substantial change in the coordination sphere. Corroborating this point, new diamagnetic signals build up between 5.6 and 8.0 ppm in ^1^H NMR spectra taken in CD_3_CN.

To further elucidate the identity of the formed species, we assessed the structure of an {Fe^II^**L**}^2+^ fragment in MeCN by reacting in this solvent the ligand **L** with the Fe(OTf)_2_ precursor bearing weakly coordinating anions. The resulting **Fe**_**MeCN**_ complex exhibits a distorted octahedral geometry, where **L** occupies three meridional positions of the inner coordination sphere, completed by three MeCN ligands. The P^1^–Fe–N bond angles are close to the expected 90° for the two axial solvent ligands (91.40 and 89.75°) but are significantly distorted for the equatorial MeCN (98.56°), as is also reported for Fe(II) PN_3_P tris-MeCN complexes (R_N_ = H; R_P_ = Ph).^32^^1^H NMR analysis of **Fe**_**MeCN**_ (Figure S11) revealed equatorial and axial MeCN signals (2.43 and 1.73 ppm, integration 1:2) in CD_2_Cl_2_ and the disappearance of these signals by exchange with the deuterated solvent in CD_3_CN (Figure S14). The Mössbauer spectrum of solid **Fe**_**MeCN**_ showed a significantly lower isomer shift than **Fe**_**Cl**_ with δ = 0.34 mm·s^–1^, as well as weak quadrupole splitting of 0.87 mm·s^–1^ (Figure S33). These parameters readily exclude a high-spin configuration but reveal a diamagnetic d^6^ low-spin configuration of **Fe**_**MeCN**_ with *S* = 0, as was already inferred from the NMR response.

Comparing the NMR spectra of **Fe**_**Cl**_ in CD_3_CN with that of an authentic **Fe**_**MeCN**_ sample in the same solvent points to identical coordination environments. The conversion of **Fe**_**Cl**_ in MeCN was further supported by crystals grown from an MeCN solution of **Fe**_**Cl**_ that reveal an inner-sphere molecular structure (Figure S37) identical with that of **Fe**_**MeCN**_.

Further NMR studies yet showed that the conversion of **Fe**_**Cl**_ into **Fe**_**MeCN**_ in MeCN is incomplete in solution; the two species are in equilibrium with other intermediates (Figure S9). We, therefore, use **Fe**_**MeCN**_ for further electrochemical analysis in MeCN to analyze a single well-defined species.

#### 2.1.5 [Co**L**Cl_2_]

**Co**_**Cl**_ coordinates in an almost ideal SBP structure with τ_5_ very close to zero. For comparison, with R_N_ = H, Rösler et al. reported a structure also resembling the ideal SBP (τ_5_ = 0.01).^36^**Co**_**Cl**_ displays a bond length of 239 pm between Co and the apical (apic) chloride atom (241 pm for the example by Rösler et al.^36^), which suggests that this ligand is only weakly bound. However, crystals prepared from the strongly coordinating MeCN solvent (Figure S38) showed neither formation of the outer-sphere chlorido complex nor substitution of the ligand by a solvent molecule but only a fractional elongation of the Co–Cl_apic_ bond to 242 pm.

**Co**_**Cl**_ exhibits heavily broadened ^1^H NMR signals in the 5–12 ppm chemical shift region (Figure S15) that are in accordance with a paramagnetic d^7^ electron configuration. Although integration of the peaks sums to the expected 33 protons, the paramagnetic character of the substance prohibited further NMR spectroscopic analysis on other nuclei.

Solid **Co**_**Cl**_ gave an effective magnetic moment of 1.9 μ_B_ at 270 K (Figure S36), consistent with the spin-only value for *S* = ^1^/_2_ (1.73 μ_B_) and in line with related complexes in the literature (an effective magnetic moment of 2.3 μ_B_ was reported for R_N_ = H).^36^ The corresponding 3d^7^ low-spin configuration of **Co**_**Cl**_ was further corroborated by the X-band EPR spectrum of the compound in an MeCN solution at 10 K, showing distinct anisotropic *g* splitting and ^59^Co hyperfine splitting (Figure S34).

We posited that the nearly ideal SBP geometry observed for **Co**_**Cl**_ is favored over distortions to higher values of τ_5_ because the corresponding structures would stabilize the singly occupied d_*z*^2^_ orbital but concomitantly destabilize the fully occupied d_*yz*_ and d_*xz*_ orbitals.^43^

#### 2.1.6 [Ni**L**Cl_2_]

**Ni**_**Cl**_ displays an SBP structure, with a distortion intermediate of **Fe**_**Cl**_ and **Co**_**Cl**_. Electronic stabilization of the d_*z*^2^_ HOMO procured by a θ_apic_ value of up to 110°^43^ is particularly favorable for the 18-electron d^8^ configuration of **Ni**_**Cl**_. Here, the apical chlorido ligand is weakly bound to the metal as well, manifesting in a bond length of 255 pm. Although the formation of a cationic almost ideally square-planar (SP) d^8^ [Ni(PN_3_P)Br]Br complex (with R_N_ = H) was observed in methanol (MeOH),^32^**Ni**_**Cl**_ crystals grown from MeCN (Figure S39) maintain the apical chlorido ligand coordinated with a marginally elongated bond distance.

NMR spectroscopy revealed a ^31^P singlet peak at 85.0 ppm (Figure S18) and well distinguished sets of ^1^H signals (Figure S16) for the different aromatic positions, most indicative for the pyridinic protons in the meta position that exhibit a characteristic doublet at 5.73 ppm (*J* = 8.2 Hz; Table 2).

#### 2.1.7 [Cu**L**Cl] and [Cu**L**I]

For Cu, the reaction of **L** with CuCl_2_ produced a diamagnetic complex, as inferred from NMR analysis, whereas a putative monomeric [Cu**L**Cl_2_] complex would be paramagnetic (d^9^). We suspect *in situ* reduction of Cu(II) to Cu(I) upon coordination to **L** with simultaneous oxidation of the ligand (additional diamagnetic signals in the ^1^H and ^31^P{^1^H} spectra), as was already reported in the literature.^44−47^ We thus aimed for the Cu(I) complex **Cu**_**Cl**_, which could be successfully synthesized starting directly from CuCl. The identical NMR spectra and crystal structures (data not shown) of the products synthesized from CuCl_2_ and CuCl supported this approach. Similarly, **Cu**_**I**_ was synthesized from CuI to introduce the less electronegative iodo ligand.

The tetracoordinated d^10^ complexes both exhibit a distorted tetrahedral (*T*_*d*_) structure, with τ_4_ values of 0.78 (chloride) and 0.77 (iodide) being intermediate between that of the ideal tetrahedron (τ_4_ = 1) and that of a butterfly/seesaw structure (τ_4_ ≈ 0.43). The coordination geometries found are in accordance with known bromide analogues (R_N_ = Me; τ_4_ = 0.78).^35^

NMR spectroscopic analysis (Figures S19–S24) reflects the similar coordination structure, with the ^31^P peaks (39.9 and 40.0 ppm for **Cu**_**Cl**_ and **Cu**_**I**_, respectively) and ^1^H peaks [e.g., 5.68 ppm (*J* = 8.0 Hz) and 5.67 ppm (*J* = 8.0 Hz) for PyH_m_ in **Cu**_**Cl**_ and **Cu**_**I**_, respectively] only marginally deviating (Table 2).

#### 2.1.8 [Zn**L**Cl_2_] and [Zn**L**(OTf)_2_]

In **Zn**_**Cl**_, the pyridine unit is not bound to the metal center, in contrast to the shared behavior of the earlier **M(II)**_**Cl**_ complexes, leaving the complex in an almost ideal tetrahedral coordination geometry (τ_4_ = 0.95). The 18-electron valence of this d^10^ configuration at the Zn center likely favors tetracoordinated structures over pentacoordinated structures. **Zn**_**Cl**_ preferentially adopts a tetrahedral structure over a SP one, possibly because HOMOs in the former (t_2_ orbitals) are significantly more stabilized than the HOMO in the latter (b_1g_ orbital). Additionally, the structural constraints of the pincer ligand might disfavor formation of the SP configuration. A bromide analogue with R_N_ = Me also exhibits the tetrahedral structure.^38^

By contrast, **Zn**_**OTf**_ retains pentacoordination and forms a 20-valence electron (VE) complex of distorted trigonal-bipyramidal (TBP) structure, as reflected in the τ_5_ value of 0.82. 20-electron complexes are only accessible in very weak field complexes, where the antibonding molecular orbital hosting two electrons is sufficiently low in energy, with the TBP geometry stabilized compared to SBP.^43^**Zn**_**OTf**_ is the only example of a pyridine-based PNP pincer complex derived from the Zn(OTf)_2_ precursor to the best of our knowledge. Therefore, the closest relatable system is the terpyridine (tpy)-coordinated Zn complex reported by Bocian et al., which crystallizes in a seesaw/butterfly structure (τ_5_ = 0.47).^48^ Here, the bond between Zn and the N atom of the pyridine core is significantly shorter than the Zn–N bond in **Zn**_**OTf**_ (204 vs 233 pm), indicating that the N is loosely coordinated in the latter system.

Distinctions in the molecular structures of **Zn**_**Cl**_ and **Zn**_**OTf**_ also can be seen in the recorded NMR spectra (Figures S25–S30), with a difference of 2.1 ppm for the ^31^P nucleus (30.9 and 28.8 ppm for **Zn**_**Cl**_ and **Zn**_**OTf**_, respectively) and 0.09 ppm [5.85 ppm (*J* = 8.1 Hz) and 5.94 ppm (*J* = 8.2 Hz) for **Zn**_**Cl**_ and **Zn**_**OTf**_, respectively] for the meta protons of the pyridine unit.

### 2.2 Electrochemical Analysis under an Ar Atmosphere

**Figure 5:**
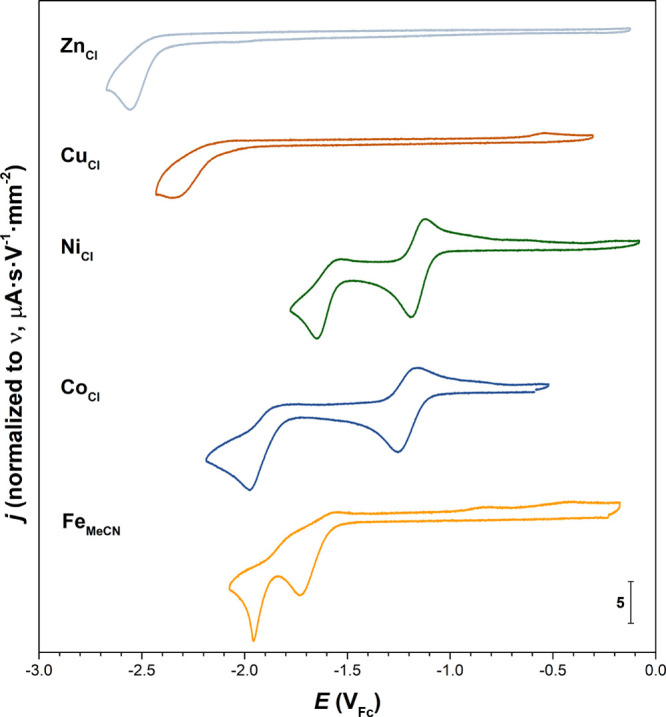
Cyclic voltammograms of selected complexes investigated in this study under an Ar atmosphere ([**M**_**X**_] = 1 mM, MeCN, 0.1 M ^*n*^Bu_4_NPF_6_, glassy carbon working electrode, and ν = 100 mV·s^–1^).

The electrochemical behaviors of **L** and **M**_**X**_ will be discussed individually in this section. The cyclic voltammograms under an Ar atmosphere of the **M**_**Cl**_ complexes bearing chlorido ligands and **Fe**_**MeCN**_ are depicted in Figure 5. The potentials of the redox events derived thereof are summarized in Table 3.

**Table 3:** Redox Potentials of the Complexes Investigated in This Study under an Ar Atmosphere^a^

	M^II/I^			M^I/0^			ligand
	*E*^0^/*E*_p,c_ (V_Fc_)	I/R	Δ*E*_p_ (mV)	*E*^0^/*E*_p,c_ (V_Fc_)	I/R	Δ*E*_p_ (mV)	*E*_p,c_ (V_Fc_)
**Mn**_**Br**_^b^							–2.64
**Fe**_**MeCN**_	–1.73	I		–1.96	I		
**Co**_**Cl**_	–1.21	R	94	–1.98	I		–2.61
**Ni**_**Cl**_	–1.16	R	69	–1.59^d^	R	114	–2.41
**Cu**_**Cl**_	–0.09	R	130				–2.35
**Cu**_**I**_	–0.03	R	128				–2.27
**Zn**_**Cl**_							–2.56
**Zn**_**OTf**_	–1.86^c^	I					–2.94

a [**M_X_**] = 1 mM, MeCN, 0.1 M ^*n*^Bu_4_NPF_6_, glassy carbon working electrode, and ν = 100 mV·s^–1^; *E*^0^ values are reported for reversible (R) waves and *E*_p,c_ for irreversible (I) waves.

b Recorded in DMF because of the poor solubility in MeCN. [**Mn**_**Br**_] = 0.5 mM.

c Likely Zn^II/0^ reduction.

d At ν = 5 V·s^–1^.

#### 2.2.1 Ligand **L**

**L** was found to be electrochemically inert in the window of potentials from −2.5 to −0.1 V versus Fc^+/0^ (abbreviated V_Fc_ in the following) in MeCN and *N*,*N*-dimethylformamide (DMF; Figure S40), thus falling in the category of redox-innocent systems.

#### 2.2.2 [Mn**L**(CO)_2_Br]

**Figure 6:**
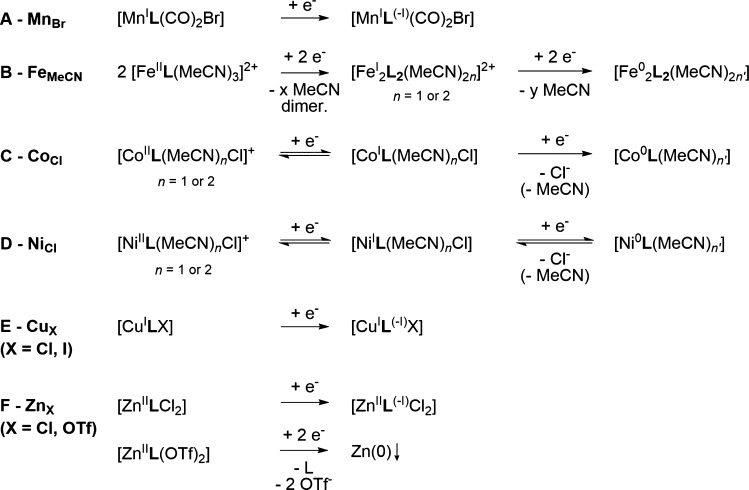
Proposed (electro)chemical steps for the reduction of complexes investigated in this study under an Ar atmosphere.

**Mn**_**Br**_ shows an irreversible reduction wave at *E*_p,c_ = −2.64 V_Fc_ (Figure S41) in CV. For related Mn complexes [Mn(tpy)(CO)_2_Br]^49^ (tpy = 2,2′:6′,2″-terpyridine) bearing the tris-chelating planar tpy ligand, a metal-centered reduction to a Mn(0) species with concomitant halide loss and subsequent fast dimerization was suggested. Nevertheless, the lack of an anodic wave for the reoxidation of a putative dimer of reduced **Mn**_**Br**_ makes such a metal-centered reduction event appear unlikely. In addition, **Mn**_**Br**_ is already in an 18-VE, alleged d^6^ low-spin configuration, and reduction would populate a high-lying e_g_ orbital. We therefore propose that reduction is centered on the ligand at the strongly cathodic potential observed here (Figure 6A).

#### 2.2.3 [Fe**L**(MeCN)_3_](OTf)_2_

**Fe**_**MeCN**_ exhibits two narrowly separated irreversible cathodic waves at *E*_p,c_ = −1.73 and −1.96 V_Fc_ (Figure 5), which we attribute to the Fe^II/I^ and Fe^I/0^ reductions, respectively, accompanied by chemical steps, viz., the loss of MeCN ligands and major structural changes. The corresponding back-oxidation waves are only visible at scan rates (ν) higher than 20 V·s^–1^, suggesting that follow-up chemical events are fast. The occurrence of structural reorganization upon reduction is also supported by the observation of a reoxidation wave at a considerably more positive potential (*E*_p,a_ = −1.24 V_Fc_) that follows reversal of the potential scan between the first and second reduction waves (Figure S44). This oxidation wave vanishes at low scan rates when the scan is reversed after the second reduction wave (Figure S45). We thus suggest that **Fe**_**MeCN**_ reduces to a [Fe^I^**L**(MeCN)_*n*_]^+^ complex (*n* = 3), which undergoes fast MeCN loss into short-lived [Fe^I^**L**(MeCN)_*n*_]^+^ with *n* = 1 or 2 (Figure 6B). Further evolution of this Fe(I) intermediate likely generates a dimeric [Fe^I^_2_**L**_2_(MeCN)_2*n*_]^2+^ compound, in the form of an Fe–Fe-bonded or an MeCN-bridged one. We tentatively attribute the oxidation wave at −1.24 V_Fc_ to the oxidation of such dimeric species. The second reduction wave is then assigned to reduction of the putative [Fe^I^_2_**L**_2_(MeCN)_2*n*_]^2+^ dimer into an [Fe^0^_2_**L**_2_(MeCN)_2*n*'_] species. When the scan reversal is negative to this second reduction, the disappearance of the reoxidation wave at −1.24 V_Fc_ for low sweep rates suggests the evolution of the postulated [Fe^0^_2_**L**_2_(MeCN)_2*n*'_] dimer into monomeric Fe(0) species or other degradation products [e.g., Fe(0) deposits]. The reversibility of the second reduction wave is gradually established when the scan rate is raised (from 5 V·s^–1^), corresponding to reoxidation of the transient Fe(0) complex, from which the follow-up chemical step is only moderately fast.

#### 2.2.4 [Co**L**Cl_2_]

In the cyclic voltammogram of **Co**_**Cl**_ (Figure 5), a partially reversible reduction wave at *E*^0^ = −1.21 V_Fc_ (Δ*E*_p_ = 94 mV) and an irreversible wave at *E*_p,c_ = −1.98 V_Fc_ can be observed, to which we assign the Co^II/I^ and Co^I/0^ couples, respectively. Although the molecular structure obtained for **Co**_**Cl**_ from an MeCN solution confirmed the inner-sphere coordination of both chlorido ligands in the solid state, we hypothesize that a dynamic ligand exchange between Cl^–^ and MeCN takes place in solution.^50^ Likely, a cationic [Co^II^**L**(MeCN)_*n*_Cl]^+^ (*n* = 1 or 2) complex forms upon the dissolution of **Co**_**Cl**_ into MeCN. We postulate that the complex reversibly reduces into a neutral [Co^I^**L**(MeCN)_*n*_Cl] species (Figure 6C). Further reduction of the Co(I) complex then proceeds concomitantly with a chemical event in an electrochemical–chemical (EC) sequence. The Co^I/0^ wave remaining irreversible even at high sweep rates (up to 100 V·s^–1^; Figure S48) suggests that the follow-up chemical step is fast. We attribute this fast chemical step to the dissociation of a chloride ligand, forming a putative [Co^0^**L**(MeCN)_*n*__'_] complex.

#### 2.2.5 [Ni**L**Cl_2_]

**Ni**_**Cl**_ shows a voltamperometric pattern at 100 mV·s^–1^ similar to that of **Co**_**Cl**_ with a reversible reduction wave at *E*^0^ = −1.16 V_Fc_, followed by an irreversible process at a more negative potential of *E*_p,c_ = −1.65 V_Fc_ (Figure 5), to which we respectively assign the Ni^II/I^ and Ni^I/0^ events. With a weakly coordinated apical chlorido ligand in the solid state, we postulate that, similar to **Co**_**Cl**_, **Ni**_**Cl**_ exchanges in a MeCN solution with a cationic [Ni^II^**L**(MeCN)_*n*_Cl]^+^ (*n* = 1 or 2) complex. The reduction of [Ni^II^**L**(MeCN)_*n*_Cl]^+^ is proposed to generate a neutral [Ni^I^**L**(MeCN)_*n*_Cl] species (Figure 6D). As stems from the irreversibility of the Ni^I/0^ event, the formation of a Ni(0) species is here also coupled to a chemical step (EC), likely a Cl^–^ elimination producing a low-valent [Ni^0^**L**(MeCN)_*n*'_] complex. In that case, however, the reversibility of the Ni^I/0^ couple can be recovered by elevating the scan rate (Figure S50), suggesting a slow chemical step. A standard potential of *E*^0^(Ni^I/0^) = −1.59 V_Fc_ is recovered from the reversible wave at a fast scan rate. The *E*_p,c_ versus log(ν) graph decays linearly in the irreversibility region (*KP*, *pure kinetics* zone of the kinetic zone diagram) with a −22.1 mV·dec^–1^ slope (Figure S51), in reasonable agreement with the theoretical value for an EC mechanism expected at −29.6 mV·dec^–1^.^51^ From this data, the apparent forward rate constant *k*_f,app_ of the coupled chemical step was estimated at *k*_f,app_(Ar) = 3.6 × 10^–1^ s^–1^.

Two more negative irreversible reduction waves appear at *E*_p,c_ = −2.41 and −2.75 V_Fc_ (Figure S42). We assign the wave at −2.41 V_Fc_ to a ligand-centered reduction, but the second reduction wave has remained unassigned so far.

#### 2.2.6 [Cu**L**Cl] and [Cu**L**I]

**Cu**_**Cl**_ exhibits an irreversible reduction wave at *E*_p,c_ = −2.35 V_Fc_ (Figure 5). At first sight, the irreversibility of the wave may be explained by Cu(I) to Cu(0) reduction with concomitant loss of the halide. While putative Cu(0) complexes are expected to decompose into heterogeneous Cu(0) deposits at the electrode surface, no substantial oxidative desorption peak commonly accounting for the oxidation of such Cu(0) deposits could be observed in the backward reaction of the investigated potential window, regardless of the scan rate. Therefore, we propose that the reduction wave relates to a reduction centered at the ligand rather than at the metal (Figure 6E). Moreover, a reversible Cu^II/I^ oxidation wave can be observed at −0.09 V_Fc_ (Figure S43). Referring to the challenging synthesis of the Cu(II) species, it is interesting that the +II oxidation state apparently can be accessed by electrochemical means. The Cu(II) complex is possibly only stable in the time scale of the CV experiment and decomposes during the reaction time of the synthesis.

**Cu**_**I**_ shows almost identical redox features compared to **Cu**_**Cl**_ with an irreversible reduction wave at *E*_p,c_ = −2.27 V_Fc_ (Figure S41), which we assume is a ligand-centered reduction as well (Figure 6E) and a reversible wave at *E*^0^ = −0.03 V_Fc_ assigned to a Cu^II/I^ couple (Figure S43).

#### 2.2.7 [Zn**L**Cl_2_] and [Zn**L**(OTf)_2_]

An irreversible reduction wave was found for **Zn**_**Cl**_ at *E*_p,c_ = −2.56 V_Fc_. d^10^ Zn ions are usually redox-innocent in accessible reduction windows, and the corresponding complexes are thus commonly used to identify the redox behavior of the coordinated ligand system.^52−54^ In particular, **Zn**_**Cl**_ is already in a stable 18-electron configuration with metal 3d orbitals fully occupied (d^10^), making a metal-centered reduction unlikely. We therefore posited that the reduction observed at low potential is centered on the ligand (Figure 6F).

The cathodic scan of **Zn**_**OTf**_ displays a reduction wave at *E*_p,c_ = −1.86 V_Fc_ (Figure S41), whose trace is crossed by that of the backward anodic scan and followed by a sharp oxidative desorption peak at higher potential (*E*_p,a_ = −0.79 V_Fc_). The observation of a line crossing and a desorption wave is diagnostic of electrodeposition upon reduction. Therefore, we assume that the reduction wave of **Zn**_**OTf**_ represents a two-electron reduction to Zn(0) with concomitant decomposition in (nanoparticulate) deposits on the electrode surface (Figure 6F). Solution dissociation of **Zn**_**OTf**_ into a dicationic complex would rationalize the positive shift in the reduction potential compared to **Zn**_**Cl**_.

#### 2.2.8 Proposed (Electro)chemical Steps upon the Reduction of **M_X_** Complexes

The proposed electrochemical and chemical steps traversed by the presented complexes upon reduction in CV are summarized in Figure 6.

Because **Fe**_**MeCN**_, **Co**_**Cl**_, and **Ni**_**Cl**_ exhibit distinct metal-centered reduction waves, which is a prerequisite for an ET_M_ mechanism in CO_2_ reduction, the electrochemical behavior of these complexes was analyzed further in the presence of CO_2_.

### 2.3 Electrochemical Analysis under a CO_2_ Atmosphere

**Figure 7:**
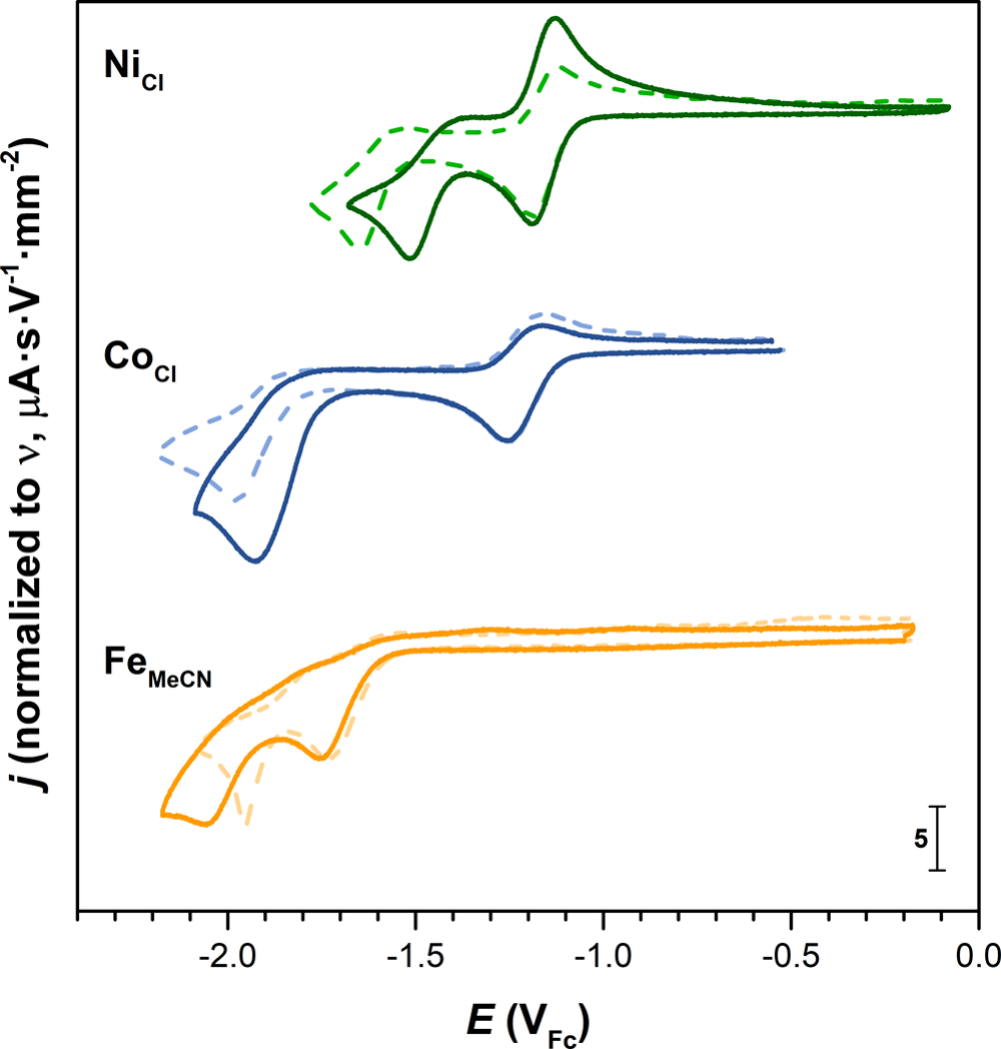
Cyclic voltammograms of selected complexes investigated in this study under Ar (dashed line) and CO_2_ (full line) atmospheres ([**M**_**X**_] = 1 mM, MeCN, 0.1 M ^*n*^Bu_4_NPF_6_, glassy carbon working electrode, and ν = 100 mV·s^–1^).

The cyclic voltammograms under a CO_2_ atmosphere for **Fe**_**MeCN**_, **Co**_**Cl**_, and **Ni**_**Cl**_ are reported in Figure 7. The shifts in the potential of the two metal-centered reduction waves upon switching of the gases Δ*E*_p,c_^Ar→CO_2_^ are summarized in Table 4.

**Table 4:** Δ*E*_p,c_^Ar→CO_2_^(M^II/I^) and Δ*E*_p,c_^Ar→CO_2_^(M^I/0^) for **M**_**X**_ under Ar and CO_2_ Atmospheres^a^

	Δ*E*_p,c_^Ar→CO_2_^(M^II/I^) (mV)	Δ*E*_p,c_^Ar→CO_2_^(M^I/0^) (mV)
**Fe**_**MeCN**_	–20	–100
**Co**_**Cl**_	<10	50
**Ni**_**Cl**_	<10	130

a [**M**_**X**_] = 1 mM, MeCN, 0.1 M ^*n*^Bu_4_NPF_6_, glassy carbon working electrode, and ν = 100 mV·s^–1^.

Under a CO_2_ atmosphere, **Fe**_**MeCN**_ exhibits potentials shifted cathodically by approximately −20 and −100 mV for the M^II/I^ and M^I/0^ couples, respectively, compared to data under an Ar atmosphere. Moreover, at a scan rate of 100 V·s^–1^, the reversibility of the two metal-centered waves is not retained to the extent observed under an Ar atmosphere (Figures S46 and S47). With a focus on the second reduction wave, a noticeable change in the reaction mechanism is observable from the change in the peak shape. While dimer formation is likely under Ar atmosphere, this pathway might be inhibited by the coordination of CO_2_ at this stage.

For **Co**_**Cl**_, the Co^II/I^ redox wave remains unaffected by CO_2_, discarding the reaction with this substrate at the Co(II) and Co(I) states. By contrast, the Co^I/0^ irreversible wave shifts by approximately 50 mV in the anodic direction upon CO_2_ saturation. This observation evidences CO_2_ coordination upon the reduction to Co(0) in an EC mechanism. Even at elevated scan rates, the Co^I/0^ wave remains irreversible (Figure S49), evidencing a fast CO_2_ association rate. The lack of knowledge of *E*_Ar_^0^(Co^I/0^) (*vide supra*) yet prevented quantification of the rate constant of CO_2_ association under our electrochemical conditions. Interestingly, however, a slight increase in the cathodic peak current (ratio *j*_p,c_ = 1.4) is also observed and suggests electrocatalytic activity. Although reductive disproportionation of CO_2_ into CO and CO_3_^2–^ is conceivable under the dry conditions used here, we suspect that traces of protons (from residual water or Hoffman degradation of the ^*n*^Bu_4_N^+^ cation) are involved in the CO_2_ electroreduction.

For **Ni**_**Cl**_, the Ni^II/I^ couple is also left unaffected by CO_2_, discarding coordination at the corresponding +II and +I oxidation states. On the contrary, **Ni**_**Cl**_ exhibits a very strong anodic shift (∼130 mV) at the Ni^I/0^ wave under CO_2_, the largest observed at the M^I/0^ couples of the analyzed complexes. At variance with the observation under an Ar atmosphere, the reversibility of the Ni^I/0^ reduction wave cannot be recovered by a faster scan rate (up to 100 V·s^–1^; Figure S52). These observations mark a clear indication of a fast CO_2_ association at the Ni(0) stage (EC). Corroborating this process, *E*_p,c_ = *f*[log(ν)] linearly decays by −38.7 mV·dec^–1^ in the *KP* zone (Figure S53). Prior knowledge of *E*_Ar_^0^(Ni^I/0^) allows one to evaluate the CO_2_ association apparent rate constant (section 2.2), found at *k*_f,app_(CO_2_) = 4.2 × 10^3^ s^–1^, which gives a bimolecular rate constant of *k*_A,f_(CO_2_) = 1.5 × 10^4^ M^–1^·s^–1^ (taking a saturation concentration at 1 atm of CO_2_ of [CO_2_] = 0.28 M in an MeCN solution^23^).

Furthermore, from scan rates above 10 V·s^–1^, an additional oxidation wave (*E*_p,a_ = −0.39 V_Fc_ for ν = 50 V·s^–1^) becomes visible. We associate this anodic wave with the oxidation of a [Ni^II^**L**(MeCN)_*n*_(CO_2_H)]^+^ or [Ni^II^**L**(MeCN)_*n*_(CO)]^2+^ (*n* = 1 or 2) adduct only accessible at elevated scan rates due to the limited lifetime of the species. We can, however, not fully exclude that this wave corresponds to the oxidation of a putative [Ni^II^**L**(MeCN)_*n*_H]^+^ hydride species formed by the reaction of a Ni(0) intermediate with residual protons.

## 3 Discussion

### 3.1 Structural and Spectroscopic Properties

**Figure 8:**
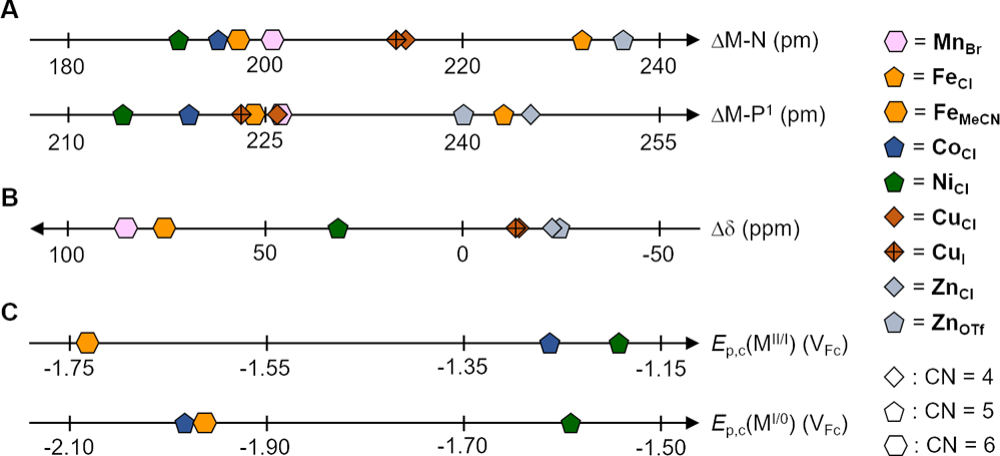
(A) Metal–ligand bond distances, (B) Δδ derived from ^31^P{^1^H} NMR spectra in CD_2_Cl_2_ (**Fe**_**Cl**_ and **Co**_**Cl**_ did not exhibit a ^31^P signal due to the paramagnetic nature of these complexes), and (C) *E*_p,c_(M^II/I^) and *E*_p,c_(M^I/0^) of applicable complexes studied in this work.

The bond distances between the coordination sites and the donor atoms (P and N) of the ligand scaffold are depicted in Figure 8A. In the subgroup of SBP complexes **M**_**Cl**_ (M = Fe, Co, and Ni), the M–P and M–N bonds shorten while the 3d row is incremented, as expected from the decreasing metal ionic radii in the 3d block.^55^ The trend is overruled as other factors such as the auxiliary ligands and coordination geometries are varied.

The ^31^P Δδ values, defined as the difference between the chemical shift of the free versus coordinated ligand (see the Supporting Information for details), show a systematic variation along with the metal series. The value decreases when the electron count is incremented from the left (**Mn**_**Br**_) to the right (**Zn**_**Cl/OTf**_) of the 3d row (Figure 8B). The observed trend is consistent with increasing electron density at the metal center within the same oxidation state when traversing from left to right in the 3d period. For the lowest 3d electron counts (Mn, Fe, and Ni), the predominant σ donation of the phosphine deshields the coordinated P and produces positive Δδ values, whereas the highest electron counts (Cu and Zn) induce an additional π-acceptor ability of the phosphine, resulting in negative Δδ values.

### 3.2 Metal-Based Reducibility

**Figure 9:**
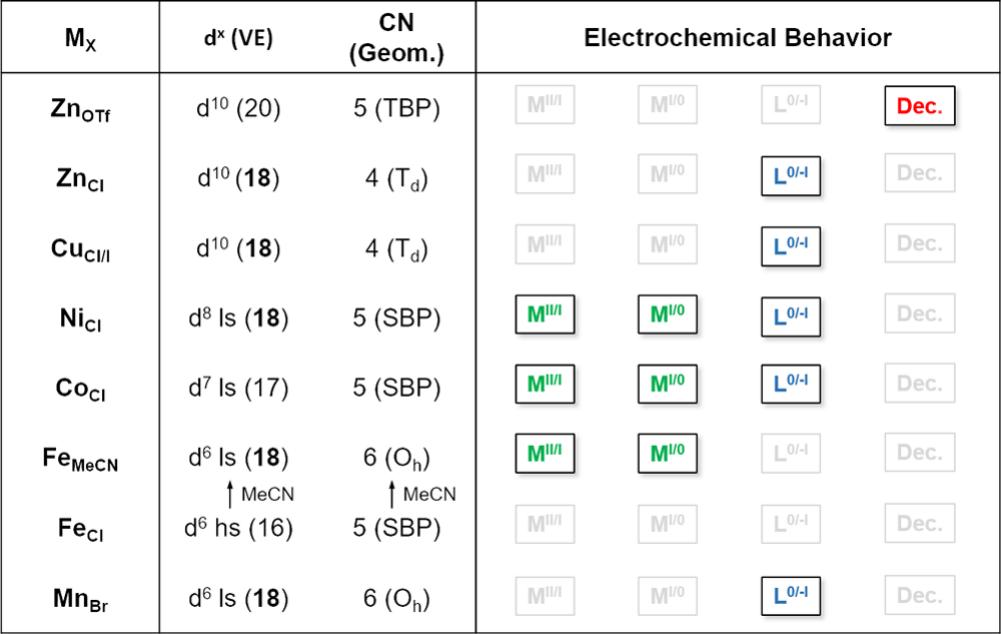
Observed electronic/geometric properties and electrochemical behavior for the complexes in this study (Dec. = decomposition).

In the series of complexes under scrutiny, metal-centered reductions are generally prevented with a 3d^10^ configuration (viz., **Cu**_**Cl**/**I**_ and **Zn**_**Cl**_) likely because of the combined saturation of the 3d metal orbitals and the valence shell (Figure 9). The ligand only reduces at very negative potentials (below −2 V_Fc_), irrespective of the metal center, confirming the redox-innocent nature of this ligand. Nevertheless, below these potentials, reduction of the ligand is possible.

Although deviating from a d^10^ configuration, the d^6^ low-spin **Mn**_**Br**_ complex also exhibits a ligand-centered reduction event only. We posited that the strongly field-splitting carbonyl ligands destabilize the unoccupied e_g_ orbitals of the metal center to the extent where ligand reduction is energetically more favorable than metal reduction, although at very cathodic potentials (<−2.5 V_Fc_). One may thus conclude that CO-coordinated Mn complexes generally require redox-active ligands (e.g., bipyridine^56^) or a cationic state^57^ to be active in CO_2_ electroreduction. This point is in sharp contrast to thermochemical CO_2_ reduction passing through the Mn–H pathway, where manganese(I) carbonyl complexes based on redox-innocent ligands are highly efficient catalysts.^58−61^

For complexes with lower ligand-field splitting, the deviation from the d^10^ state unlocks metal-centered reduction processes, as observed in the case of **Fe**_**MeCN**_, **Co**_**Cl**_, and **Ni**_**Cl**_ (Figure 9). Here we note that Fe, Co, and Ni complexes are among the most prominent eCO_2_R catalysts.^17^ While ligand-centered reductions at mildly negative potentials are commonly proposed for Fe, Co, or Ni complexes^17,25,30,31,50,62,63^ (e.g., with polypyridinic ligands having delocalized, low-energy π* orbitals), in our series, a comparison with the other complexes (of Mn, Cu, and Zn) allows assignment of the first two reductions (Figure 8C) solely to metal-centered orbitals in **Fe**_**MeCN**_, **Co**_**Cl**_, and **Ni**_**Cl**_.

### 3.3 Electrochemical CO_2_ Activation

Complex **Fe**_**MeCN**_ shows an Fe^II/I^ voltamperometric wave that is affected by the presence of CO_2_ (although marginally), indicating coordination at the Fe(I) state. With **Fe**_**MeCN**_ adopting a distorted octahedral geometry in a d^6^ low-spin configuration, the one-electron reduction from Fe(II) to Fe(I) populates an e_g_ antibonding orbital. In the presence of CO_2_, the fast chemical step deduced from the irreversibility of the Fe^II/I^ wave is consistent with the high reactivity of Fe(I) species. The activation of CO_2_ at Fe(I) centers reported previously, namely, in eCO_2_R, has been mainly observed with complexes bearing redox-active ligands,^64^ which are thus best described as ligand-reduced Fe(II) complexes. For the redox-inactive pincer ligand used here, we postulate that the metal-centered radical binds CO_2_ to form [Fe^I^**L**(MeCN)(CO_2_)]^+^ as the primary intermediate. We note that one of the rare reported metal-centered Fe(I) radicals is known to result in the bimolecular cleavage of CO_2_ into a (μ-CO)(μ-O)-bridged Fe(II) dimer^65^ and a similar bimolecular CO_2_ activation with **Fe**_**MeCN**_ also seems possible.

**Figure 10:**
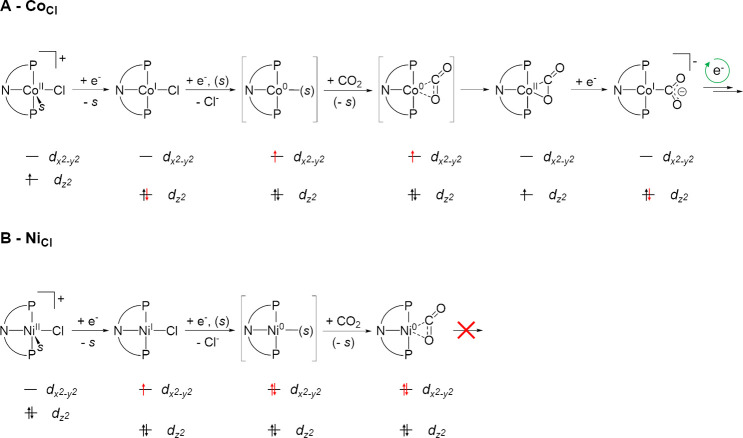
Proposed electrochemical pathways with schematic frontier orbital diagrams for the reduction of (A) **Co**_**Cl**_ and (B) **Ni**_**Cl**_ under a CO_2_ atmosphere (*s* = MeCN).

Moving from Fe to Co in **Co**_**Cl**_, CO_2_ activation occurs only at the M(0) state instead of M(I). The slightly distorted SBP geometry determined for **Co**_**Cl**_ in the solid state is expected to be retained in an MeCN solution, possibly with chloride and solvent in dynamic exchange at the apical position. The HOMO is consequently a single occupied d_*z*^2^_ one at the d^7^ configuration of paramagnetic **Co**_**Cl**_. The first reduction is expected to fill this d_*z*^2^_ orbital, producing a diamagnetic [Co^I^**L**(MeCN)Cl] complex (Figure 10A). Release of the apical ligand would stabilize the now fully occupied d_*z*^2^_ orbital in a SP [Co^I^**L**Cl] 16-electron complex, as found for the related pincer complex [Co^I^(PN_3_P)(MeCN)]^+^ (R_N_ = H; R_P_ = ^*t*^Bu).^66^ The large potential gap to the second reduction in a Co(0) species [*E*_p,c_(Co^I/0^) – *E*^0^(Co^II/I^) = −670 mV] is consistent with the need for the injection of a second electron into a high-lying antibonding d_*x*^2^–*y*^2^_ orbital. This second reduction is followed by an irreversible chemical step (EC), most probably the expulsion of Cl^–^ concomitant with MeCN association to generate a neutral [Co^0^**L**(MeCN)] complex. Calculations on similar [Co^0^(PNP)(MeCN)] complexes suggest a η^2^ π-bonded MeCN in a structure best described as a cobalt(II) cycloimine.^66^ We postulate that MeCN binding rather than Cl^–^ expulsion is rate-determining. The positive shift of the irreversible Co^I/0^ reduction (*pure kinetics* conditions, *KP*) under CO_2_ indicates that the associated chemical equilibrium is more shifted or accelerated forward with that substrate. The electrophilic nature of the C atom of CO_2_ likely makes the association faster compared to that of MeCN. Although formally metalloradicals, Co(0) species do not commonly exhibit single-electron-transfer reactivity but undergo two-electron oxidative chemistry.^67^ We thus favor formulation of the CO_2_ activation product as a d^7^ [Co^II^**L**(CO_2_)] complex. The potential at which this intermediate is accessed is low enough for further reduction into a 16-electron [Co^I^**L**(CO_2_)]^−^ complex, refilling the low-lying d_*z*^2^_ orbital (Figure 10A). This feature would provide a direct entry into a catalytic cycle for CO_2_ reduction, as supported by an increased magnitude of the current density at the Co^I/0^ wave. We note, however, that the current enhancement observed in the presence of CO_2_ may also result from a faradaic (and not catalytic) two-electron reduction from [Co^I^**L**Cl] to [Co^I^**L**(CO_2_)]^−^.

Complex **Ni**_**Cl**_ also displays a SBP coordination geometry and similar to Co interacts with CO_2_ only at the 0 oxidation state. **Ni**_**Cl**_ is reduced down to a Ni(0) species by two consecutive electron injections expected to fill the d_*x*^2^–*y*^2^_ orbital (Figure 10B), a fact corroborated by reduced states computed on a [Ni(PN_3_P)Br_2_] complex bearing a close PN_3_P ligand (R_N_ = H or Me; R_P_ = Ph).^68^ Compared to **Co**_**Cl**_, the potential gap between the +I and 0 oxidation states is tighter [*E*^0^(Ni^I/0^) – *E*^0^(Ni^II/I^) = −430 mV] (Figure 8C), consistent with electronic increment in the same high-lying d_*x*^2^–*y*^2^_ orbital. The complex is expected to shuttle through a [Ni^I^**L**Cl] intermediate, either in a seesaw or a SP geometry, as suggested by structures found for a similar set of Ni(I) PNP halide complexes.^69^ Upon reduction to the Ni(0) state, we assume Cl^–^ dissociation and MeCN association similar to that proposed for Co(0). However, the apparent rate of these follow-up chemical steps [*k*_f,app_(Ar) = 3.6 × 10^–1^ s^–1^] is decelerated compared to the Co(0) complex, as witnessed by reversibility of the Ni^I/0^ wave at an elevated scan rate. This point also agrees with the formation of an intermediate closed-shell 18-electron Ni(0) species, more stable than the 17-electron Co(0) congener. The binding of CO_2_ at this stage is, however, largely favored, with **Ni**_**Cl**_ displaying the largest cathodic shift Δ*E*_p,c_^Ar→CO_2_^(M^I/0^) of 130 mV. This shift translates into a follow-up chemical step 4 orders of magnitude faster upon CO_2_ addition [*k*_f,app_(CO_2_) = 4.2 × 10^3^ s^–1^], corresponding to the binding of the electrophilic CO_2_ substrate. CO_2_ activation at a Ni(0) center supported by a neutral PPP ligand was shown to build a pentacoordinated Ni(0) species displaying η^2^-CO_2_ binding,^70^ as crystallographically shown in the famous Aresta complex.^71^ Such species are better described as Ni(0) species with minor electron transfer to the bound CO_2_. We suggest that **Ni**_**Cl**_ results in a similar [Ni^0^**L**(CO_2_)] adduct. The reoxidation wave at *E*_p,a_ = −0.39 V_Fc_ observed at an elevated scan rate (ν = 50 V·s^–1^) could then relate to the oxidation of a subsequent intermediate, for instance, a [Ni^II^**L**(CO_2_H)]^+^ hydroxycarbonyl complex formed by the protonation of [Ni^0^**L**(CO_2_)].

### 3.4 Comparative Assessment

The groups of Lewis and Fujita have emphasized the relationship between the reduction potential of the metal center and CO_2_ coordination on a series of nickel(II) and cobalt(II) cyclam-like complexes.^23,72−75^ The CO_2_ association equilibrium [*K*_A_(CO_2_)] and forward rate [*k*_A,f_(CO_2_)] constants at the +I oxidation state were found to follow a general trend, increasing as the M^II/I^ standard potential becomes more negative.^74^ The present series of complexes **Fe**_**MeCN**_, **Co**_**Cl**_, and **Ni**_**Cl**_ exhibit reduction potentials sufficiently negative to afford CO_2_ coordination. While interaction with CO_2_ occurs at the +I oxidation state for **Fe**_**MeCN**_, both **Co**_**Cl**_ and **Ni**_**Cl**_ require reduction to a formal M(0) state to proceed to CO_2_ activation. This point is in sharp contrast to complexes of the same metals with redox-innocent ligands, where CO_2_ coordination already occurs at the formal M(I) state. In particular, [Ni(cyclam)]^+^, which has a Ni^II/I^ reduction potential [*E*^0^(Ni^II/I^) = −1.14 V_NHE_^75^ corrected to approximately −1.77 V_Fc_^76^] close to the Ni^I/0^ one of **Ni**_**Cl**_ [*E*^0^(Ni^I/0^) = −1.59 V_Fc_], exhibits a Ni^I^-CO_2_ association rate constant [*k*_A,f_(CO_2_) = 3.2 × 10^7^ M^–1^·s^–1^, in aqueous solution^73^] exceeding by several orders of magnitude that found for Ni^0^-CO_2_ with **Ni**_**Cl**_ [*k*_A,f_(CO_2_) = 1.5 × 10^4^ M^–1^·s^–1^, in an MeCN solution]. We note here that equilibrium and rate constants for CO_2_ association at 3d centers in their 0 oxidation state are scarcely reported (we only found reported an order of magnitude of 10^2^–10^3^ s^–1^ for the CO_2_ association rate constant at the electroreduced 0 state of a [Co^II^(PN_3_P)(MeCN)_2_]^2+^ complex).^66^ Nevertheless, this difference likely reflects the higher kinetic barrier for CO_2_ association at the closed-shell [Ni^0^**L**(MeCN)_*n*'_] complex compared to the open-shell 17-VE [Ni^I^(cyclam)]^+^, underlining the importance of the electronic configuration for binding and activating CO_2_.

In agreement with this consideration, the present results indicate that the 17-VE complex [Co^0^**L**(MeCN)_*n*'_] binds CO_2_ at faster rates than the 18-VE complex [Ni^0^**L**(MeCN)_*n*'_]. A larger degree of CO_2_ activation is also suggested by the increase in the Co^I/0^ cathodic peak current upon contact with the substrate. This interpretation is in line with findings for the series of cyclam-like complexes in which Co(I) transfers up to two electrons to the bound CO_2_, whereas Ni(I) does not substantially activate the substrate.^75^ The electrochemical CO_2_ activation by **Co**_**Cl**_ and **Ni**_**Cl**_ within the redox-innocent ligand structure appears to follow the same trend. The two-electron reduction of **Ni**_**Cl**_ at mildly negative potential generates a [Ni^0^**L**(η^2^-CO_2_)] complex that cannot be reduced further at the potential of the Ni^I/0^ wave, whereas [Co^II^**L**(η^2^-CO_2_)] is further reducible at the potential of the Co^I/0^ event and can lead to electrocatalytic turnover.

From an organometallic view, these points relate in the case of **Ni**_**Cl**_ to a coordination of CO_2_ at Ni(0) characterized by weak-to-moderate back-bonding according to the Dewar–Chatt–Duncanson model of π-bond coordination. The Ni center remains then in the formal oxidation state 0. By contrast, with **Co**_**Cl**_, the more pronounced electron transfer from the Co(0) metal to the antibonding orbitals of the C=O bond of CO_2_ results in a metallaoxirane-type structure. Consequently, the Co(0) center is oxidized to Co(II), and subsequent uptake of another electron can generate cobalt(I) metallacarboxylate as a potential intermediate for catalytic CO_2_ reduction.

**Figure 11:**
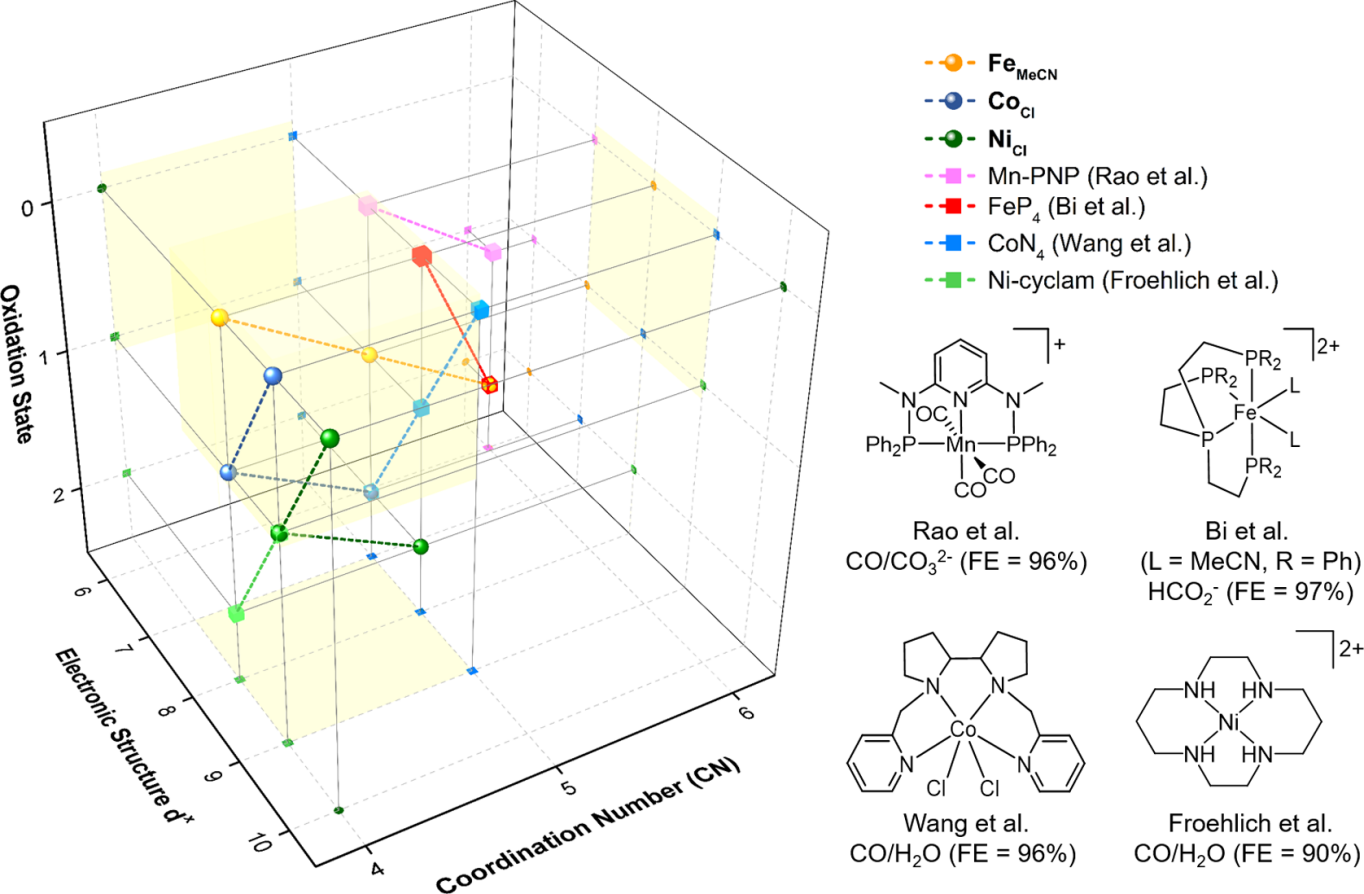
Electronic and geometric properties of **Fe**_**MeCN**_, **Co**_**Cl**_, **Ni**_**Cl**_, and selected eCO_2_R catalysts traversing the ET_M_ mechanism (except Bi et al.) and containing redox-innocent ligand systems.^57,77−79^ Metal-centered reduction processes are indicated by dashed lines. The yellow area indicates core electronic/structural properties favoring electrochemical CO_2_ activation.

In Figure 11, we graphically summarize the interplay between the electronic and structural properties of 3d metal complexes based on redox-innocent ligands with their electrochemical CO_2_ activation properties. The data comprise the complexes reported in this work (**Fe**_**MeCN**_, **Co**_**Cl**_, and **Ni**_**Cl**_) and literature reports of active CO_2_ reduction electrocatalysts. The metal-based redox steps to connect the starting complex and the active species are indicated by the dashed line. All complexes having activity toward CO_2_ activation under electrocatalytic conditions show common features within the yellow box indicated in the graph. They are coordinatively unsaturated with coordination number (CN) = 4 or 5, have an open-shell structure of metal electron configuration d^7^ to d^9^, and are reduced to low oxidation states +I or 0. Consequently, potential electrocatalysts for CO_2_ reduction should be designed to meet these three parameters simultaneously upon access of the active species. This combination appears to be a necessary prerequisite but is not sufficient to guarantee the electrocatalytic performance. Structural or electronic changes under reaction conditions, viz., by ligand dissociation/exchange or dimerization, must also be considered in the design of CO_2_ electroreduction catalysts engaging in the ET_M_ mechanism. The molecular tools of coordination and organometallic chemistry will be essential to address this challenge.

## 4 Summary and Conclusion

The series of 3d transition metals M = Mn, Fe, Co, Ni, Cu, and Zn form stable complexes with the redox-innocent pincer ligand **L**. Analysis of the structures of the new ligand and coordination compounds with each metal was achieved by XRD and spectroscopic investigation. We have then put into perspective the structures of the obtained complexes with the requirements for electrochemical CO_2_ activation in the ET_M_ pathway, namely, metal-centered reducibility and CO_2_ activation at the reduced states.

The reduction ability at the metal center was found to be primarily dependent on the 3d electron count of the starting complex. The redox innocence of the metal illustrates this point in full-shell d^10^ Cu(I) and Zn(II) species, whereas open-shell complexes **Fe**_**MeCN**_, **Co**_**Cl**_, and **Ni**_**Cl**_ afford metal-centered reduction. This factor alone is still not decisive because the d^6^ complex **Mn**_**Br**_ could not be reduced at mild potentials from the initial +I oxidation state likely because of the strong field splitting of the ancillary carbonyl ligands. We emphasize that this behavior strongly deviates from manganese(I) carbonyl complexes that are based on redox-active ligands and are highly competent in CO_2_ electroreduction.^80^

Complexes **Fe**_**MeCN**_, **Co**_**Cl**_, and **Ni**_**Cl**_ that reveal distinct metal-centered redox events from M(II) down to M(0) were found to bind CO_2_ in their reduced states, as inferred from the electrochemical data. The coordination of CO_2_ occurs already at an Fe(I) species in **Fe**_**MeCN**_ but happens at the M(0) state for **Co**_**Cl**_ and **Ni**_**Cl**_. The capability of activating CO_2_ by transferring electron(s) to the molecule further depends on the metal. Formed at mildly negative potential, the d^10^ Ni species leads to the association of CO_2_ to a putative Aresta-type Ni^0^-η^2^-CO_2_ complex, resulting in only moderate electron transfer to CO_2_ through π-back-bonding. This activation mode appears to be insufficient to enable electrocatalytic activity. The d^9^ Co(0) intermediate is evolved at 330 mV more negative potential and, after further electron uptake, can lead to a formal cobalt(I) metallacarboxylate complex, which is able to promote turnover.

Our findings, together with the few literature examples of molecular eCO_2_R catalysts based on redox-innocent ligands, single out that an unsaturated coordination sphere (CN = 4 or 5) and a d^7^-to-d^9^ configuration at the reduced state (+I or 0) are characteristic for 3d metal complexes enabling an ET_M_ mechanism. The design of complexes that purposely meet these three characteristics simultaneously provides a promising strategy for catalyst development. However, dynamic structural and electronic changes under electrochemical conditions must also be controlled to ensure the primary operation of the metal centers in the desired catalytic manifold. This challenge may be approached by optimizing the molecular framework of a privileged coordination environment through systematic ligand variation, as is well-established for numerous examples in organometallic catalysis. Along these lines, investigations are underway in our laboratory to benchmark the metal-centered properties and structural variations of the ligand lead structure against the electrocatalytic performance.

## 5 Experimental Section

### 5.1 General Considerations

#### 5.1.1 Synthesis and Structural Analysis

All synthetic manipulations were performed under an Ar atmosphere either in an MBraun UNILAB Plus glovebox or by use of standard Schlenk techniques in oven-dried glassware, ensuring rigorously inert conditions. Organic solvents were dried and degassed by passage over an MBraun SPS-7 solvent purification system, handled under an Ar atmosphere, and stored over molecular sieves. Commercially available chemicals were purchased from Merck, Carl Roth, TCI, or abcr and used without further purification if not otherwise stated. NMR solvents were degassed by three freeze–pump–thaw cycles and dried over molecular sieves. Aniline was dried over molecular sieves and degassed by purging with Ar. Tetrabutylammonium hexafluorophosphate was dried and degassed at 80 °C under vacuum for 12 h.

NMR spectra were recorded on a Bruker AVANCE NEO 400 MHz or a Bruker AVANCE III HD 500 MHz NMR spectrometer with a Bruker Prodigy probe at the indicated temperature. Chemical shifts (δ) are given in parts per million related to tetramethylsilane (TMS) and the coupling constants (*J*) in hertz. The solvent residual signal was used as a reference, and the chemical shift was converted to the TMS scale (CD_2_Cl_2_, δ_H_ = 5.32 ppm and δ_C_ = 53.84 ppm; CD_3_CN, δ_H_ = 1.94 ppm and δ_C_ = 1.32 ppm).^81^ First-order spin multiplicities are abbreviated as singlet (s), doublet (d), triplet (t), and quadruplet (q). Couplings of higher-order or overlapped signals are denoted as m (multiplet) and broadened signals as br. Indications of the positions of the H and C atoms in aromatic rings are given as ortho (o), meta (m), para (p), and quaternary (q).

HRMS was recorded on a Thermo Scientific Q Exactive Plus Hybrid Quadrupole-Orbitrap mass spectrometer. UV/vis measurements were conducted on an Agilent Technologies Cary 8454 UV/vis spectroscopy system.

Elemental analysis (C, H, and N) was performed on an Elementar UNICUBE fitted with a thermal conductivity detector.

Single crystals of compounds CCDC 2109420–2109432 were selected under a microscope in polarized light with an applied nitrogen cryostream at approximately −40 °C and covered with polyfluorinated polyether. The crystals were picked up with nylon loops and rapidly mounted in the nitrogen cold gas stream of the diffractometer at 100 K to prevent solvent loss. A Bruker D8 Venture diffractometer equipped with a IμS3 Diamond source, INCOATEC Helios mirror optics (Mo Kα radiation; λ = 0.71073 Å), and a Photon III detector were used for data collection. Data were processed using the Bruker *APEX 3* software suite. The final cell constants are based on refinement of the *XYZ* centroids of several thousand reflections above 20 σ(*I*). Structures were solved and refined using the embedded Bruker *SHELXTL* software package. All non-H atoms were anisotropically refined, and H atoms were placed at calculated positions and refined as riding atoms with isotropic displacement parameters.

The magnetic susceptibility data were measured with powder samples in the temperature range 2–270 K by using a SQUID susceptometer with a field of 1.0 T (MPMS-3, Quantum Design, calibrated with a standard palladium reference sample; error <2%). Multiple-field variable-temperature magnetization measurements were done at 1, 4, and 7 T in the range of 2–260 K with the magnetization equidistantly sampled on a 1/*T* temperature scale. Sample holders of quartz with O-ring sealing were used, and the SQUID response curves (raw data) have been corrected for holder and solvent contributions by subtracting the corresponding response curves obtained from separate measurements without sample material. The experimental magnetization data obtained from an independent simulation of the corrected SQUID response curves were corrected for underlying diamagnetism by use of tabulated Pascal’s constants, as well as for temperature-independent paramagnetism. Handling and simulation of the SQUID raw data as well as spin-Hamiltonian simulation of the susceptibility and magnetization data were done with our own package *julX.SL* for exchange-coupled systems (available from E.B. by emailing ebill@gwdg.de).

^57^Fe Mössbauer spectra were recorded with nonenriched powder samples on a conventional spectrometer with alternating constant acceleration of the γ source (^57^Co/Rh, 1.8 GBq). The source was kept at rt, and the sample temperature was maintained constant in an Oxford Instruments Variox cryostat. The raw data (512 channels) were folded to merge the two recorded mirror images of the spectra, which also eliminates the parabolic background. The minimum experimental linewidth was 0.24 mm·s^–1^ (full width at half-maximum). Isomer shifts are quoted relative to iron metal at 300 K because the spectrometer was calibrated by recording the Mössbauer spectrum of a 12-μm-thick foil of α-Fe at rt, with the center of the six-line pattern being taken as zero velocity. The *mf.SL* package (version 2.2 by E.B.) was used to simulate the spectra with Lorentzian doublets, or doublet Voigt profiles, with least-squares parameter optimization.

X-band EPR derivative spectra were recorded with frozen-solution samples (ca. 1 mM) on a Bruker ELEXSYS E500 spectrometer equipped with the Bruker dual-mode cavity (ER4116DM) and a helium-flow cryostat (Oxford Instruments ESR 910). The microwave unit was the Bruker high-sensitivity Super-X bridge (ER-049X) with an integrated microwave frequency counter. The magnetic field controller (ER032T) was externally calibrated with a Bruker NMR field probe (ER035M). The spectra were simulated with the program *esimX.SL* (by E.B.) for calculation of the powder spectra with effective *g* values and first-order hyperfine splitting and anisotropic linewidths (Gaussian line shapes were used).

#### 5.1.2 Electrochemical Analysis

Electrochemical investigations were performed on a BioLogic VSP-300 potentiostat equipped with an analogue ramp generator for high potential scan rate analysis in a standard three-electrode setup with a glassy carbon working electrode (WE), a platinum wire counter electrode (CE), and a Ag/AgNO_3_ (10 mM AgNO_3_ in a solution of 0.1 M ^*n*^Bu_4_NPF_6_ in the electrochemical solvent) reference electrode (RE). The WE was polished over a polishing pad using an alumina suspension before rinsing with ultrapure water from a Milli-Q Advantage A10 water purification system and then with ethanol. RE and CE were rinsed with ultrapure water and ethanol. Lastly, each electrode was dried under a stream of argon before insertion into the cell. The ohmic drop of the electrochemical cell was estimated and compensated for (85%) by the *iR* compensation loop embedded in the potentiostat.

In general, a 1 mM solution of the analyte in a dry and degassed solution of 0.1 M ^*n*^Bu_4_NPF_6_ in the electrochemical solvent was prepared for analysis. Prior to the addition of the analyte, the electrolyte solution was purged by bubbling a solvent-saturated argon flow through silicon tubing under vigorous stirring. The scanned potential window was adjusted according to the visible redox waves, and parameter variations are indicated in the respective measurements. Cyclic voltammograms were typically recorded at a scan rate of 0.1 V·s^–1^. For experiments under CO_2_, the electrolyte solution was sparged with a solvent-saturated CO_2_ flow for 20 min before measurements were performed.

At the end of a measurement row, ferrocene (1 mM) was added as the internal potential reference.

### 5.2 Synthesis

#### 5.2.1 Ligand

##### *N*^2^,*N*^6^-Diphenylpyridine-2,6-diamine

The synthesis was performed according to a modified procedure of Wagaw and Buchwald.^40^ 2,6-Dibromopyridine (0.4978 g, 2.00 mmol, 1.00 equiv), aniline (0.3725 g, 0.37 mL, 4.00 mmol, 2.00 equiv), tris(dibenzylideneacetone)dipalladium [Pd_2_(dba)_3_; 0.0366 g, 0.0400 mmol, 0.0200 equiv], 1,3-bis(diphenylphosphino)propane (dppp; 0.0330 g, 0.0800 mmol, 0.0400 equiv), and potassium *tert*-butoxide (0.6284 g, 5.60 mmol, 2.80 equiv) were placed in a Schlenk tube in the glovebox, and toluene was added (15 mL). The suspension was stirred at 100 °C for 18 h and cooled to rt before dichloromethane (DCM; 10 mL) was added. The organic phase was washed with brine (20 mL), the resulting aqueous phase was extracted with DCM (3 × 10 mL), and the combined organic layers were dried over anhydrous magnesium sulfate. After removal of the organic solvents *in vacuo*, the crude product was purified by column chromatography on silica using a mixture of pentane and ethyl acetate (85:15). The desired product was received as an orange solid (0.397 g, 1.52 mmol, 76%). The obtained analytical data are consistent with those previously reported in the literature.^40^

##### *N*^2^,*N*^6^-Bis(diphenylphosphanyl)-*N*^2^,*N*^6^-diphenylpyridine-2,6-diamine (**L**)

*n*-Butyllithium (2.5 M in hexanes, 4.0 mL, 10.0 mmol, 2.0 equiv) was added dropwise to an orange solution of *N*^2^,*N*^6^-diphenylpyridine-2,6-diamine (1.3067 g, 5.00 mmol, 1.00 equiv) in THF (35 mL) at −78 °C. The solution was allowed to warm up to rt and stirred for 1 h before cooling down again to 0 °C and dropwise addition of chlorodiphenylphosphine (1.84 mL, 10.0 mmol, 2.0 equiv). The solution was allowed to warm up to rt and stirred at rt for 1 h, then at 65 °C for 18 h. After cooling to rt, the solvents were removed *in vacuo*, and the brown residue was washed with MeOH (4 × 10 mL), diethyl ether (Et_2_O; 3 × 5 mL), and pentane (3 × 5 mL). Removal of the residual solvents *in vacuo* yielded the desired product **L** as a white solid (2.1 g, 3.3 mmol, 67%). Crystals suitable for XRD were obtained from a concentrated THF/pentane (3:1) solution at −35 °C. ^1^H NMR (500 MHz, CD_2_Cl_2_, 296 K): δ_H_ 7.28–7.16 (m, 12H, PPh*H*_m_ + PPh*H*_p_), 7.15–7.07 (m, 9H, PPh*H*_o_ + Py*H*_p_), 7.02–6.95 (m, 6H, NPh*H*_m_ + NPh*H*_p_), 6.82–6.76 (m, 4H, NPh*H*_o_), 5.81 (d, 2H, *J* = 8.0, Py*H*_m_). ^13^C{^1^H} NMR (126 MHz, CD_2_Cl_2_, 296 K): δ_C_ 160.3–159.7 (m, Py*C*_q_), 143.1–142.8 (m, NPh*C*_q_), 139.5–138.8 (m, PPh*C*_q_), 138.6 (Py*C*_p_), 133.9–133.3 (m, PPh*C*_m_), 130.9 (NPh*C*_o_), 129.1 (NPh*C*_m_), 128.8 (PPh*C*_p_), 128.0 (t, *J* = 2.9, PPh*C*_o_), 126.4 (NPh*C*_p_), 101.5 (Py*C*_m_). ^31^P{^1^H} NMR (202 MHz, CD_2_Cl_2_, 296 K): δ_P_ 52.8. HRMS (ESI^+^). Calcd for C_41_H_33_N_3_P_2_ + H^+^: *m*/*z* 630.22225. Found: *m*/*z* 630.22213. Elem anal. Calcd for C_41_H_33_N_3_P_2_: C, 78.21; H, 5.28; N, 6.67. Found: C, 78.36; H, 5.15; N, 6.64.

#### 5.2.2 Complexes

**General procedure**: **L** (0.0315 g, 0.0500 mmol, 1.00 equiv) and the respective metal precursor (0.0500 mmol, 1.00 equiv) were placed in a Schlenk tube in a glovebox, and the respective solvent (2 mL) was added. The resulting solution was stirred at the indicated temperature for 16 h before removal of the solvent *in vacuo*. Purification steps and deviations from the general procedure are explicated below for each complex.

[Mn**L**(CO)_2_Br] (**Mn_Br_**): Reaction conditions: Mn(CO)_5_Br, toluene, 110 °C. The desired product **Mn**_**Br**_ was obtained as a yellow solid after the residue was washed with DCM (2 mL) and pentane (3 × 2 mL) prior to removal of the solvent *in vacuo*. Yield: 30.8 mg, 0.0375 mmol, 75%. In concentrated solutions of DCM and under exposure to light, a change of color to brown, possibly upon CO loss and dimerization, was observed. Crystals suitable for XRD were obtained from the vapor diffusion of pentane into a concentrated DCM solution under exclusion of light. ^1^H NMR (500 MHz, CD_2_Cl_2_, 296 K): δ_H_ 7.77 (dd, 4H, *J* = 5.8 and 5.1), 7.47–7.36 (m, 10H), 7.36–7.27 (m, 8H), 7.26–7.13 (m, 7H, Py*H*_p_), 6.81 (d, 2H, *J* = 8.0, NPh*H*_o_), 5.79 (d, 2H, *J* = 8.2, Py*H*_m_). ^13^C{^1^H} NMR (126 MHz, CD_2_Cl_2_, 296 K): δ_C_ 178.0 (*C*O), 164.1 (t, *J* = 12.9, Py*C*_q_), 141.3 (NPh*C*_q_), 139.9 (t, *J* = 23.4, PPh*C*_q_), 139.1 (Py*C*_p_), 137.5 (t, *J* = 6.3), 132.5, 131.8, 131.7 (t, *J* = 5.7), 131.5, 130.6, 130.3, 130.2, 130.1, 128.8, 128.2 (t, *J* = 4.6), 127.4 (t, *J* = 5.0), 102.9 (Py*C*_m_). ^31^P{^1^H} NMR (202 MHz, CD_2_Cl_2_, 296 K): δ_P_ 138.9. HRMS (ESI^+^). Calcd for C_43_H_33_BrMnN_3_O_2_P_2_^+^: *m*/*z* 819.06064. Found: *m*/*z* 819.06076. Elem anal. Calcd for C_43_H_33_BrMnN_3_O_2_P_2_·0.1 C_7_H_8_: C, 63.26; H, 4.11; N, 5.06. Found: C, 63.66; H, 4.44; N, 4.99.

[Fe**L**Cl_2_] (**Fe_Cl_**): Reaction conditions: FeCl_2_, DCM, rt. The crude product was purified by precipitation from a DCM solution with pentane. **Fe**_**Cl**_ was obtained as a yellow solid after the precipitate was washed with pentane (3 × 2 mL) and the solvent was removed *in vacuo*. Yield: 30.8 mg, 0.0476 mmol, 95%. Crystals suitable for XRD were obtained from a concentrated DCM/pentane (3:1) solution at −35 °C. ^1^H NMR (500 MHz, CD_2_Cl_2_, 296 K): δ_H_ 57.14 (s br, 2H, Py*H*_m_), 14.38 (s br, 8H, PPh*H*_m_), 8.61 (s br, 4H, NPh*H*_m_), 3.52 (s br, 4H, NPh*H*_o_), 3.13 (s br, 2H, NPh*H*_p_), −4.92 (s br, 8H, PPh*H*_o_), – 5.45 (s br, 4H, PPh*H*_p_), −11.61 (s br, 1H, Py*H*_p_). ^13^C{^1^H} NMR (126 MHz, CD_2_Cl_2_, 296 K): δ_C_ 282.7, 258.5, 199.5, 155.0, 152.1, 116.9, 108.6. HRMS (ESI^+^). Calcd for C_41_H_33_ClFeN_3_P_2_^+^: *m*/*z* 720.11821. Found: *m*/*z* 720.11849. Elem anal. Calcd for C_41_H_33_Cl_2_FeN_3_P_2_: C, 65.10; H, 4.40; N, 5.56. Found: C, 65.31; H, 4.79; N, 5.28.

Crystals of **[FeL(MeCN)**_**3**_**](Cl**_**3**_**FeOFeCl**_**3**_**)** were obtained from a concentrated MeCN/Et_2_O (3:1) solution of **Fe**_**Cl**_ at −30 °C under air.

[Fe**L**(MeCN)_3_](OTf)_2_ (**Fe_MeCN_**): Reaction conditions: Fe(OTf)_2_, MeCN, rt. The desired product **Fe**_**MeCN**_ was obtained as an orange solid after washing with Et_2_O (2 mL) and pentane (3 × 2 mL) prior to the removal of the solvent *in vacuo*. Yield: 48.9 mg, 0.0442 mmol, 88%. Crystals suitable for XRD were obtained from a concentrated MeCN/Et_2_O (3:1) solution at −35 °C. ^1^H NMR (500 MHz, CD_2_Cl_2_, 298 K): δ_H_ 7.75–7.59 (m, 12H, PPh*H*_o_ + PPh*H*_p_), 7.60–7.50 (m, 8H, PPh*H*_m_), 7.48–7.37 (m, 6H, NPh*H*_m_ + NPh*H*_p_), 7.34 (t, 1H, *J* = 8.2, Py*H*_p_), 7.10 (d, 4H, *J* = 7.4, NPh*H*_o_), 5.99 (d, 2H, *J* = 8.2, Py*H*_m_), 2.43 (s, 3H, C*H*_3_CN_eq_), 1.73 (s, 6H, C*H*_3_CN_ax_). ^13^C{^1^H} NMR (126 MHz, CD_2_Cl_2_, 296 K): δ_C_ 166.0 (t, *J* = 11.1, Py*C*_q_), 141.8 (Py*C*_p_), 139.2 (t, *J* = 2.7, NPh*C*_q_), 138.7 (CH_3_*C*N_eq_), 138.1 (CH_3_*C*N_ax_), 134.1 (t, *J* = 6.3, PPh*C*_o_), 132.9 (PPh*C*_p_), 131.1 (NPh*C*_m_), 130.5 (NPh*C*_o_), 129.9 (NPh*C*_p_), 129.7 (t, *J* = 2.7, PPh*C*_m_), 129.5 (PPh*C*_q_), 129.3, 104.5 (t, *J* = 2.3, Py*C*_m_), 5.4 (*C*H_3_CN_eq_), 4.7 (*C*H_3_CN_ax_). ^31^P{^1^H} NMR (202 MHz, CD_2_Cl_2_, 296 K): δ_P_ 129.2. HRMS (ESI^+^). Calcd for C_43_H_36_FeN_4_P_2_^2+^: *m*/*z* 363.08768. Found: *m*/*z* 363.08752. Elem anal. Calcd for C_49_H_42_F_6_FeN_6_O_6_P_2_S_2_·0.25 C_4_H_10_O: C, 53.37; H, 3.99; N, 7.47. Found: C, 53.04; H, 4.33; N, 7.12.

[Co**L**Cl_2_] (**Co_Cl_**): Reaction conditions: CoCl_2_, THF, rt. The crude product was purified by precipitation with pentane from a THF solution. **Co**_**Cl**_ was obtained as a dark-red solid after the precipitate was washed with pentane (3 × 2 mL) and the solvent was removed *in vacuo*. Yield: 34.0 mg, 0.0448 mmol, 90%. Crystals suitable for XRD were obtained from the vapor diffusion of pentane into a concentrated THF solution or from a concentrated MeCN/Et_2_O (3:1) solution at −35 °C.

^1^H NMR (400 MHz, CD_2_Cl_2_, 296 K): δ_H_ 10.65 (br s, 1H), 9.40 (br s, 4H), 8.61 (br s, 4H), 8.34–7.07 (m, 16H), 5.97 (br s, 8H). HRMS (ESI^+^). Calcd for C_41_H_33_Cl_2_CoN_3_P_2_^+^: *m*/*z* 758.08533. Found: *m*/*z* 758.08561. Elem anal. Calcd for C_41_H_33_Cl_2_CoN_3_P_2_: C, 64.84; H, 4.38; N, 5.53. Found: C, 64.56; H, 4.27; N, 5.48.

[Ni**L**Cl_2_] (**Ni_Cl_**): Reaction conditions: NiCl_2_·DME, DCM, rt. The desired product **Ni**_**Cl**_ was obtained as a red solid after the crude product was precipitated from a solution of DCM with pentane, followed by washing of the residue with pentane (3 × 2 mL) and removal of the solvent *in vacuo*. Yield: 34.2 mg, 0.0450 mmol, 90%. Crystals suitable for XRD were obtained from a concentrated DCM/pentane (3:1) solution or a concentrated MeCN/Et_2_O (3:1) solution at −35 °C.

^1^H NMR (500 MHz, CD_2_Cl_2_, 296 K): δ_H_ 7.90 (d, 8H, *J* = 6.3, PPh*H*_o_), 7.63 (t, 4H, *J* = 7.4, PPh*H*_p_), 7.48 (t, 8H, *J* = 7.5, PPh*H*_m_), 7.41–7.31 (m, 3H, NPh*H*_p_ + Py*H*_p_), 7.27 (t, 4H, *J* = 7.5, NPh*H*_m_), 6.92 (d, 4H, *J* = 7.6, NPh*H*_o_), 5.73 (d, 2H, *J* = 8.2, Py*H*_m_). ^13^C{^1^H} NMR (126 MHz, CD_2_Cl_2_, 296 K): δ_C_ 163.8 (Py*C*_q_), 142.8 (Py*C*_p_), 137.9 (NPh*C*_q_), 134.7 (PPh*C*_o_), 133.1 (PPh*C*_p_), 130.6 (NPh*C*_o_), 130.1 (NPh*C*_m_), 129.6 (NPh*C*_p_), 129.2 (PPh*C*_m_), 126.2 (t, *J* = 23.7, PPh*C*_q_), 103.4 (Py*C*_m_). ^31^P{^1^H} NMR (202 MHz, CD_2_Cl_2_, 296 K): δ_P_ 85.0. HRMS (ESI^+^). Calcd for [C_41_H_33_Cl_2_N_3_NiP_2_ – Cl]^+^: *m*/*z* 722.11862. Found: *m*/*z* 722.11871. Elem anal. Calcd for C_41_H_33_Cl_2_N_3_NiP_2_·0.2 CH_2_Cl_2_: C, 63.75; H, 4.34; N, 5.41. Found: C, 63.55; H, 4.57; N, 5.43.

[Cu**L**Cl] (**Cu_Cl_**): Reaction conditions: CuCl, THF, rt. The desired product **Cu**_**Cl**_ was obtained as a light-yellow solid after precipitation from a solution in DCM with pentane and washing of the residue with pentane (3 × 2 mL) before removal of the residual solvent *in vacuo*. Yield: 23.9 mg, 0.0327 mmol, 66%. Crystals suitable for XRD were obtained from a concentrated DCM/pentane (3:1) solution at −35 °C.

^1^H NMR (500 MHz, CD_2_Cl_2_, 296 K): δ_H_ 7.50 (dd, 8H, *J* = 5.8 and 5.3, PPh*H*_o_), 7.31 (t, 4H, *J* = 7.4, PPh*H*_p_), 7.24 (t, 1H, *J* = 8.0, Py*H*_p_), 7.17 (t, 8H, *J* = 7.6, PPh*H*_m_), 7.15–7.08 (m, 6H, NPh*H*_m_ + NPh*H*_p_), 6.93–6.83 (m, 4H, NPh*H*_o_), 5.68 (d, 2H, *J* = 8.0, Py*H*_m_). ^13^C{^1^H} NMR (126 MHz, CD_2_Cl_2_, 296 K): δ_C_ 157.8 (t, *J* = 9.4, Py*C*_q_), 141.6 (Py*C*_p_), 140.1 (t, *J* = 3.0, NPh*C*_q_), 133.6 (PPh*C*_o_), 132.9 (t, *J* = 11.4, PPh*C*_q_), 130.9 (NPh*C*_o_), 130.4 (PPh*C*_p_), 129.7 (NPh*C*_m_), 128.5 (t, *J* = 4.4, PPh*C*_m_), 127.9 (NPh*C*_p_), 101.1 (Py*C*_m_). ^31^P{^1^H} NMR (202 MHz, CD_2_Cl_2_, 296 K): δ_P_ 39.9. HRMS (ESI^+^). Calcd for C_41_H_33_ClCuN_3_P_2_^+^: *m*/*z* 727.11288. Found: *m*/*z* 727.11272. Elem anal. Calcd for C_41_H_33_ClCuN_3_P_2_·0.05 CH_2_Cl_2_: C, 67.27; H, 4.55; N, 5.73. Found: C, 67.02; H, 4.75; N, 5.48.

[Cu**L**I] (**Cu_I_**): Reaction conditions: CuI, THF, 65 °C. The desired product **Cu**_**I**_ was obtained as a white solid by precipitating the crude product from a DCM solution with pentane, washing the residue with pentane (3 × 2 mL), and removing the solvent *in vacuo*. Yield: 21.3 mg, 0.0260 mmol, 52%. Crystals suitable for XRD were obtained from a concentrated THF solution at −35 °C.

^1^H NMR (500 MHz, CD_2_Cl_2_, 296 K): δ_H_ 7.62–7.41 (m, 8H, PPh*H*_o_), 7.36–7.23 (m, 5H, PPh*H*_p_ + Py*H*_p_), 7.21–7.06 (m, 14H, PPh*H*_m_ + NPh*H*_m_ + NPh*H*_p_), 7.01–6.89 (m, 4H, NPh*H*_o_), 5.67 (d, 2H, *J* = 8.0, Py*H*_m_). ^13^C{^1^H} NMR (126 MHz, CD_2_Cl_2_, 296 K): δ_C_ 157.7 (t, *J* = 9.2, Py*C*_q_), 141.6 (Py*C*_p_), 140.0 (t, *J* = 2.7, NPh*C*_q_), 133.7 (PPh*C*_o_), 132.6 (t, *J* = 11.7, PPh*C*_q_), 130.9 (NPh*C*_o_), 130.4 (PPh*C*_p_), 129.7 (NPh*C*_m_), 128.4 (t, *J* = 4.4, PPh*C*_m_), 127.9 (NPh*C*_p_), 101.2 (Py*C*_m_). ^31^P{^1^H} NMR (202 MHz, CD_2_Cl_2_, 296 K): δ_P_ 40.0. HRMS (ESI^+^). Calcd for C_41_H_33_CuIN_3_P_2_^+^: *m*/*z* 819.04849. Found: *m*/*z* 819.04742. Elem anal. Calcd for C_41_H_33_CuIN_3_P_2_: C, 60.05; H, 4.06; N, 5.12. Found: C, 59.82; H, 4.18; N, 5.06.

[Zn**L**Cl_2_] (**Zn_Cl_**): Reaction conditions: ZnCl_2_, THF, rt. The desired product **Zn**_**Cl**_ was obtained as a white solid by precipitating the crude product from a solution of DCM with pentane, followed by washing of the residue with pentane (3 × 2 mL) and removal of the solvent *in vacuo*. Yield: 33.3 mg, 0.0434 mmol, 87%. Crystals suitable for XRD were obtained from a concentrated THF/pentane (3:1) solution at −35 °C.

^1^H NMR (500 MHz, CD_2_Cl_2_, 296 K): δ_H_ 7.46–7.39 (m, 8H, PPh*H*_o_), 7.34–7.28 (m, 5H, PPh*H*_p_ + Py*H*_p_), 7.24–7.08 (m, 14H, PPh*H*_m_ + NPh*H*_m_ + NPh*H*_p_), 7.03–6.90 (m, 4H, NPh*H*_o_), 5.85 (d, 2H, *J* = 8.1, Py*H*_m_). ^13^C{^1^H} NMR (126 MHz, CD_2_Cl_2_, 296 K): δ_C_ 157.4 (t, *J* = 7.3, Py*C*_q_), 141.4 (Py*C*_p_), 138.7 (NPh*C*_q_), 133.8 (PPh*C*_o_), 131.6 (PPh*C*_p_), 131.2 (NPh*C*_o_), 129.9 (NPh*C*_m_), 128.9 (t, *J* = 4.9, PPh*C*_m_), 128.4 (NPh*C*_p_), 101.8 (Py*C*_m_). ^31^P{^1^H} NMR (202 MHz, CD_2_Cl_2_, 296 K): δ_P_ 30.9. HRMS (ESI^+^). Calcd for [C_41_H_33_N_3_P_2_ZnCl_2_ – Cl]^+^: *m*/*z* 728.11242. Found: *m*/*z* 728.11180. Elem anal. Calcd for C_41_H_33_Cl_2_N_3_P_2_Zn·0.25 CH_2_Cl_2_: C, 62.94; H, 4.29; N, 5.34. Found: C, 62.67; H, 4.30; N, 5.33.

[Zn**L**(OTf)_2_] (**Zn_OTf_**): Reaction conditions: Zn(OTf)_2_, MeCN, 80 °C. The desired product **Zn**_**OTf**_ was obtained as a white solid by precipitating the crude product from a solution of DCM with pentane, washing the residue with pentane (3 × 2 mL), and removing the solvent *in vacuo*. Yield: 47.4 mg, 0.0477 mmol, 95%. Crystals suitable for XRD were obtained from a concentrated THF solution at −35 °C.

^1^H NMR (500 MHz, CD_2_Cl_2_, 296 K): δ_H_ 7.45–7.36 (m, 13H, PPh*H*_o_ + PPh*H*_p_ + Py*H*_p_), 7.25 (t, 8H, *J* = 7.7, PPh*H*_m_), 7.22–7.14 (m, 6H, NPh*H*_m_ + NPh*H*_p_), 7.03 (s br, 4H, NPh*H*_o_), 5.94 (d, 2H, *J* = 8.2, Py*H*_m_). ^13^C{^1^H} NMR (126 MHz, CD_2_Cl_2_, 296 K): δ_C_ 157.5 (t, *J* = 6.8, Py*C*_q_), 143.2 (Py*C*_p_), 138.0 (t, *J* = 2.4, NPh*C*_q_), 133.9 (br, PPh*C*_q_), 132.5 (PPh*C*_o_), 130.7 (br, NPh*C*_o_), 130.3 (NPh*C*_m_), 129.2 (t, *J* = 5.2, PPh*C*_m_), 129.0 (PPh*C*_p_), 126.0 (NPh*C*_p_), 120.1 (q, *C*F_3_), 102.9 (Py*C*_m_). ^31^P{^1^H} NMR (202 MHz, CD_2_Cl_2_, 296 K): δ_P_ 28.8. HRMS (ESI^+^). Calcd for [C_43_H_33_F_6_N_3_O_6_P_2_S_2_Zn – CF_3_SO_3_]^+^: *m*/*z* 842.09559. Found: *m*/*z* 842.09613. Elem anal. Calcd for C_43_H_33_F_6_N_3_O_6_P_2_S_2_Zn: C, 52.00; H, 3.35; N, 4.23. Found: C, 52.26; H, 3.68; N, 4.32.

## Supporting Information Available

^1^H, ^13^C and ^31^P NMR, UV/vis, EPR, and Mössbauer spectra, magnetic susceptibility measurements, additional XRD molecular structures and XRD data, and additional CV and electrochemical analysis (PDF)

## Accession Codes

CCDC 2109420–2109432 contain the supplementary crystallographic data for this paper. These data can be obtained free of charge via www.ccdc.cam.ac.uk/data_request/cif, or by emailing data_request@ccdc.cam.ac.uk, or by contacting The Cambridge Crystallographic Data Centre, 12 Union Road, Cambridge CB2 1EZ, UK; fax: +44 1223 336033.
