# Supplementary material for: Systematic Variation of 3d Metal Centers in a Redox-Innocent Ligand Environment: Structures, Electrochemical Properties, and Carbon Dioxide Activation
Source: Inorg Chem. 2021 Dec 1;60(24):19062–78. doi: 10.1021/acs.inorgchem.1c02909 (PMC8693193; doi:10.1021/acs.inorgchem.1c02909)
Supplement: Supplementary file 1 — ic1c02909_si_001.pdf [file ic1c02909_si_001.pdf]

Supporting Information: Systematic Variation of 3d Metal Centers in a Redox-Innocent Ligand Environment: Structures, Electrochemical Properties and CO_2 Activation

Niklas W. Kinzel,[a, b] Derya Demirbas,[c] Eckhard Bill,[a] Thomas Weyhermüller,[a] Christophe Werlé,[a, d] Nicolas Kaeffer,[a] and Walter Leitner*[a, b]*

[a] Max Planck Institute for Chemical Energy Conversion, Stiftstr. 34–36, 45470 Mülheim an der Ruhr, Germany. Emails: nicolas.kaeffer@cec.mpg.de and walter.leitner@cec.mpg.de

[b] Institut für Technische und Makromolekulare Chemie (ITMC), RWTH Aachen University, Worringer Weg 2, 52074 Aachen, Germany.

[c] Max-Planck-Institut für Kohlenforschung, Kaiser-Wilhelm-Platz 1, 45470 Mülheim an der Ruhr, Germany.

[d] Ruhr University Bochum, Universitätsstr. 150, 44801 Bochum, Germany.

Table of Content

1. Structural Characterization 3
1.1. Nuclear Magnetic Resonance (NMR) 3
1.1.1. L 3
1.1.2. $[MnL(CO)_2Br]$ 5
1.1.3. $[FeLCl_2]$ 6
1.1.4. $[FeL(MeCN)_3](OTf)_2$ 9
1.1.5. $[CoLCl_2]$ 11
1.1.6. $[NiLCl_2]$ 12
1.1.7. [CuLCl] 14
1.1.8. [CuLI] 16
1.1.9. $[ZnLCl_2]$ 18
1.1.10. $[ZnL(OTf)_2]$ 20
1.1.11. Coordination Chemical Shift 21
1.2. UV/VIS 22
1.3. Mössbauer 23
1.4. Electron Paramagnetic Resonance (EPR) 25
1.5. Magnetic Susceptibility Measurements 26
1.5.1. $[FeLCl_2]$ 26
1.5.2. $[CoLCl_2]$ 27
1.6. X-ray Diffraction 27
1.6.1. Structures 27
1.6.2. Data and Refinements 29
1.6.3. Calculation of Geometry Indices 42
2. Electrochemical Analysis 43
2.1. Cyclic Voltammograms 43
2.1.1. Ligand and Complexes in Extended Potential Windows 43
2.1.2. $[FeL(MeCN)_3](OTf)_2$ 46
2.1.3. $[CoLCl_2]$ 48
2.1.4. $[NiLCl_2]$ 49
2.2. Calculations of Rate Constants for Follow-Up Reactions 51

1. Structural Characterization

1.1. Nuclear Magnetic Resonance (NMR)

1.1.1. L

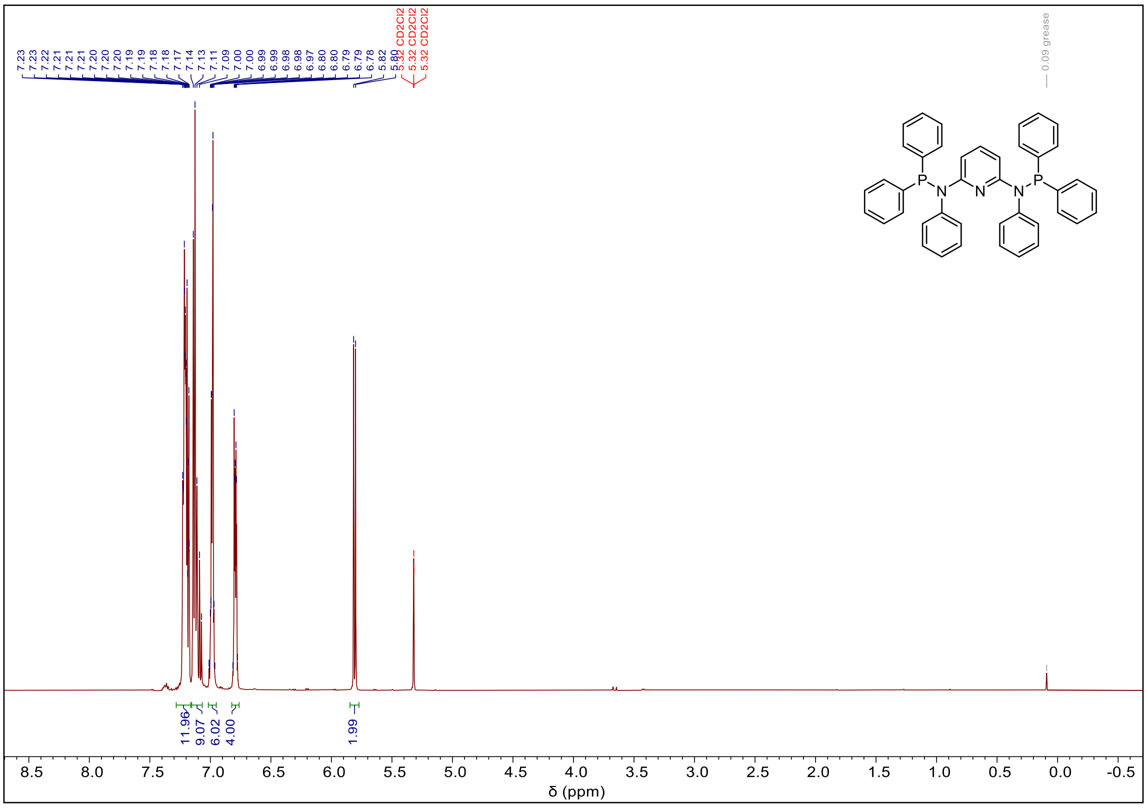

Figure S1. ^{1}H NMR spectrum of **L** in CD_2Cl_2 at 500 MHz and 296 K.

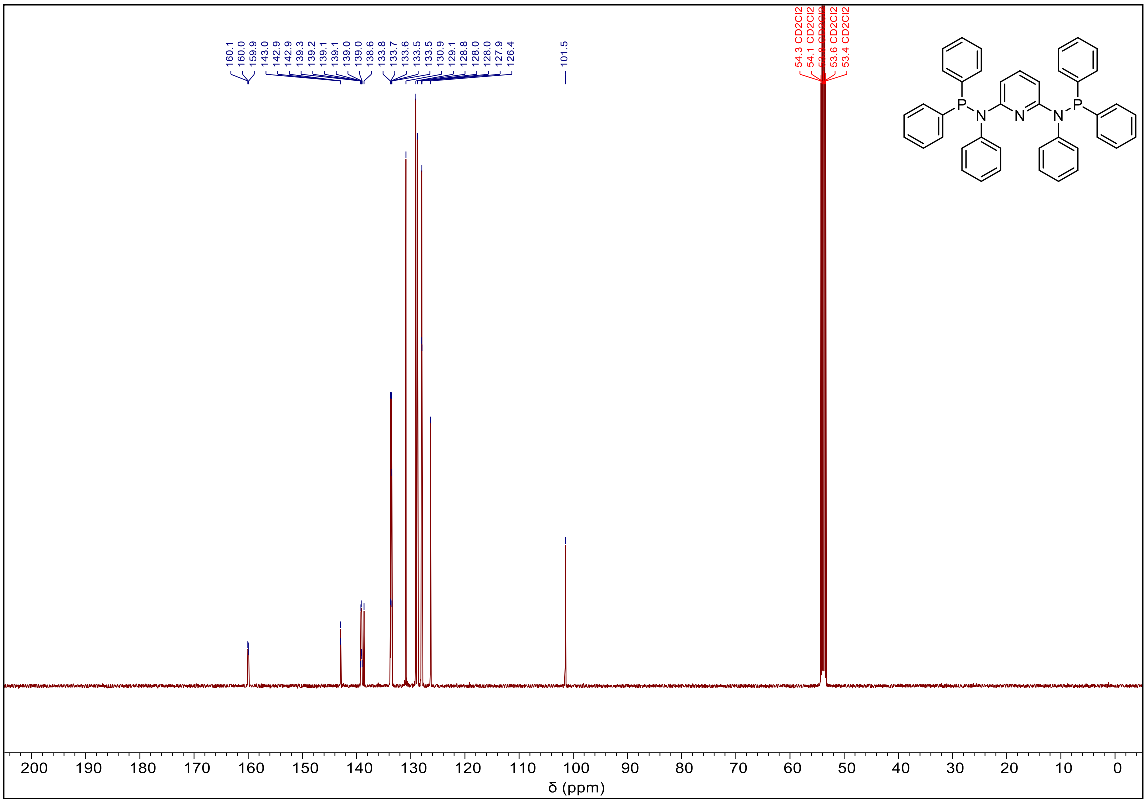

Figure S2. $^{13}C\{^{1}H\}$ NMR spectrum of **L** in CD_2Cl_2 at 126 MHz and 296 K.

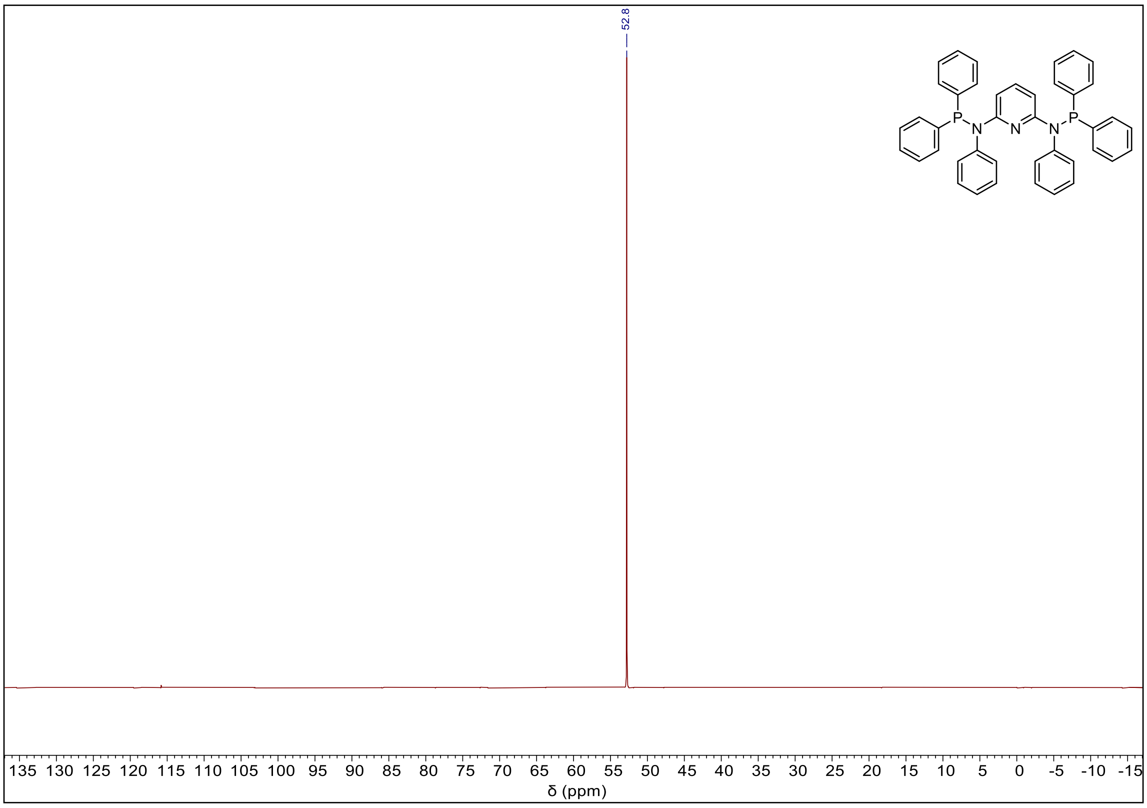

Figure S3. $^{31}P\{^{1}H\}$ NMR spectrum of **L** in CD_2Cl_2 at 202 MHz and 296 K.

1.1.2. [MnL(CO)$_2$Br]

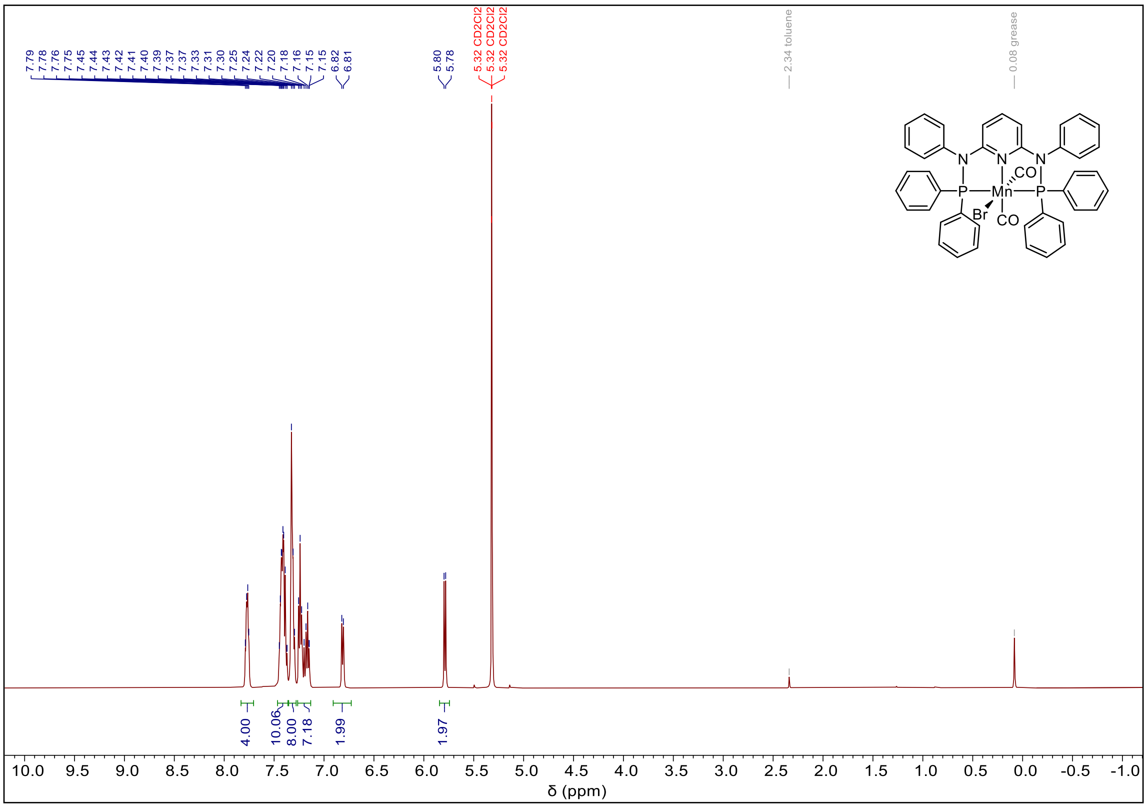

Figure S4. ^{1}H NMR spectrum of **Mn$_{Br}$** in CD_2Cl_2 at 500 MHz and 296 K.

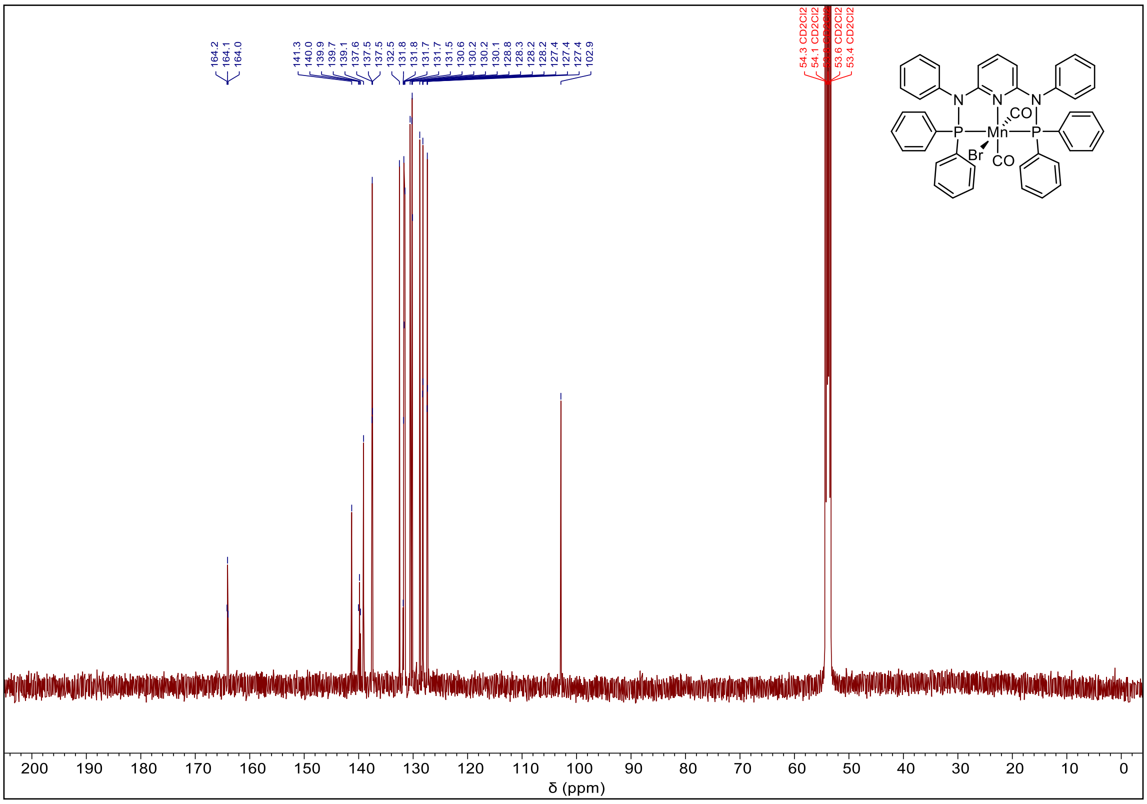

Figure S5. ^{13}C{^{1}H} NMR spectrum of **Mn$_{Br}$** in CD_2Cl_2 at 126 MHz and 296 K.

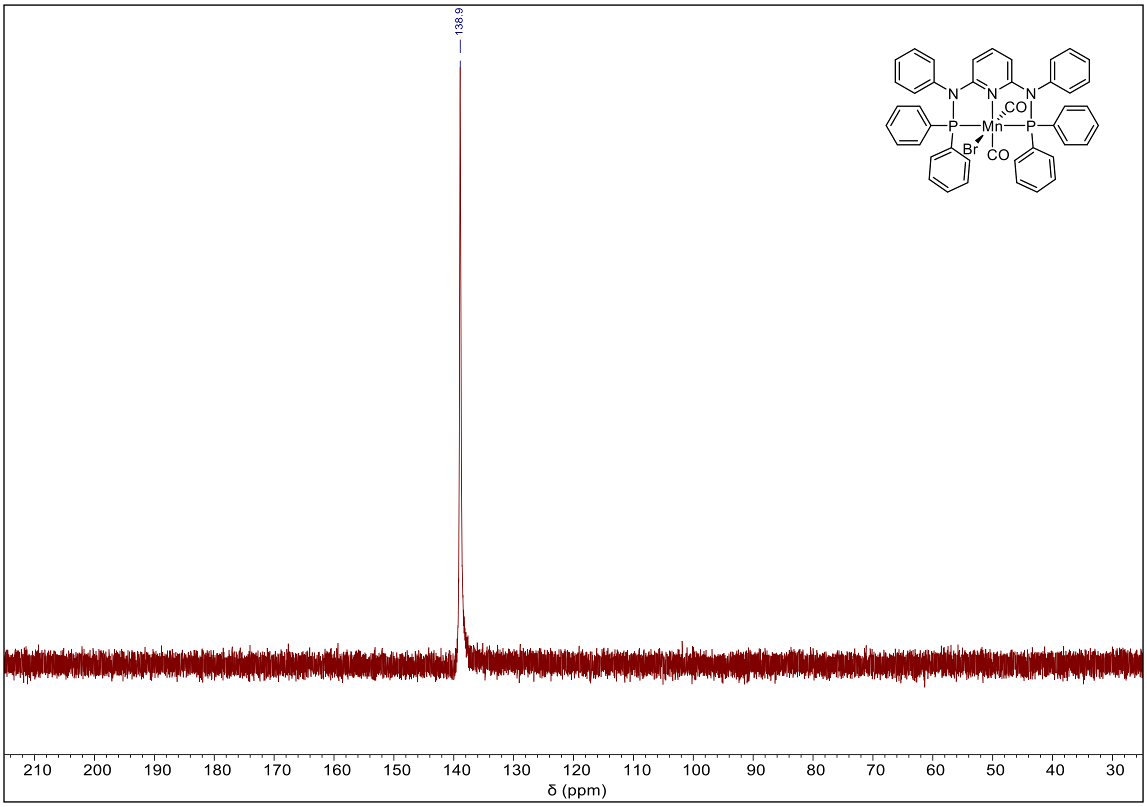

Figure S6. $^{31}P\{^{1}H\}$ NMR spectrum of $\mathbf{Mn_{Br}}$ in CD_2Cl_2 at 202 MHz and 296 K.

1.1.3. $[FeLCl_2]$

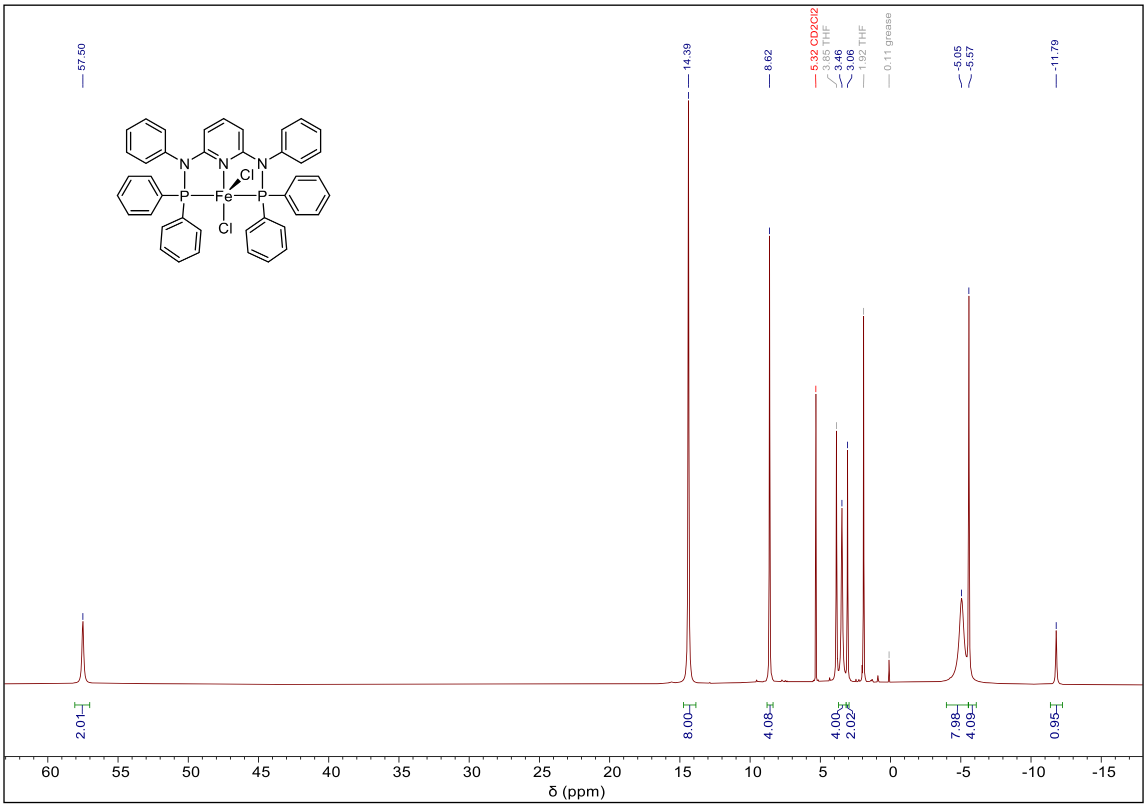

Figure S7. ^{1}H NMR spectrum of $\mathbf{Fe_{Cl}}$ in CD_2Cl_2 at 500 MHz and 296 K.

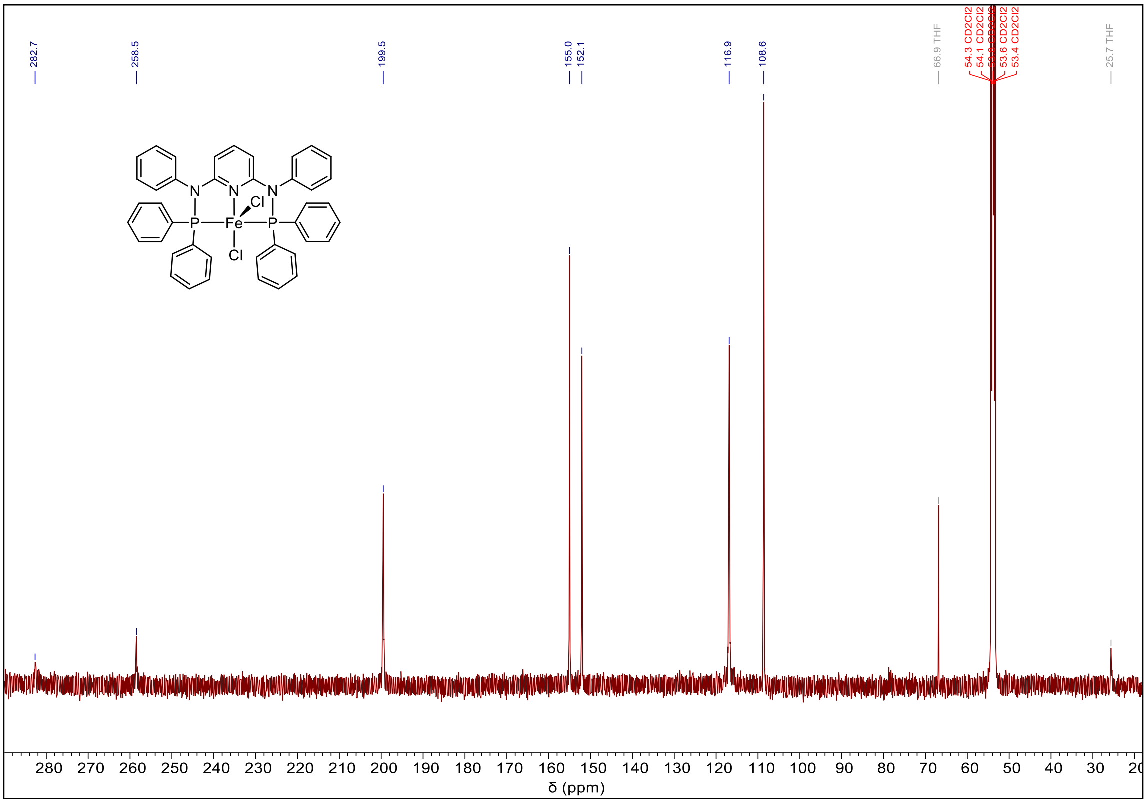

Figure S8. $^{13}C\{^{1}H\}$ NMR spectrum of **Fe$_{Cl}$** in CD_2Cl_2 at 126 MHz and 296 K.

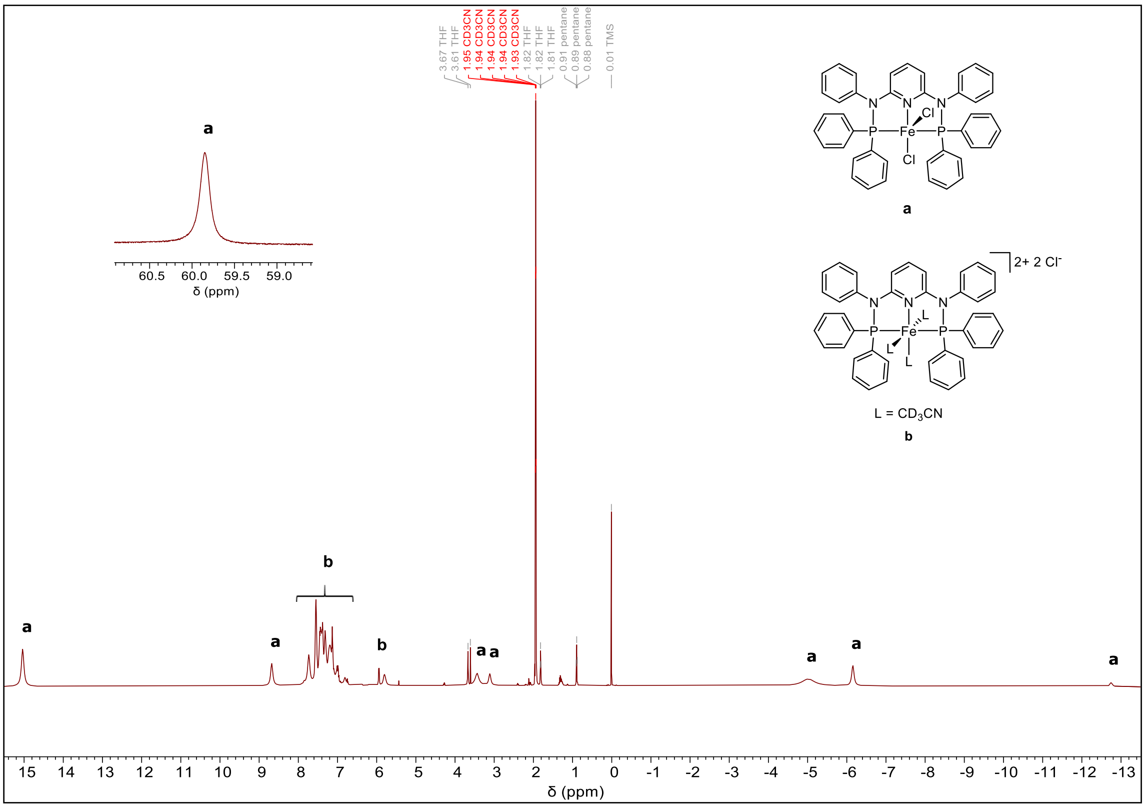

Figure S9. ^{1}H NMR spectrum of **Fe$_{Cl}$** in CD_3CN at 500 MHz and 296 K.

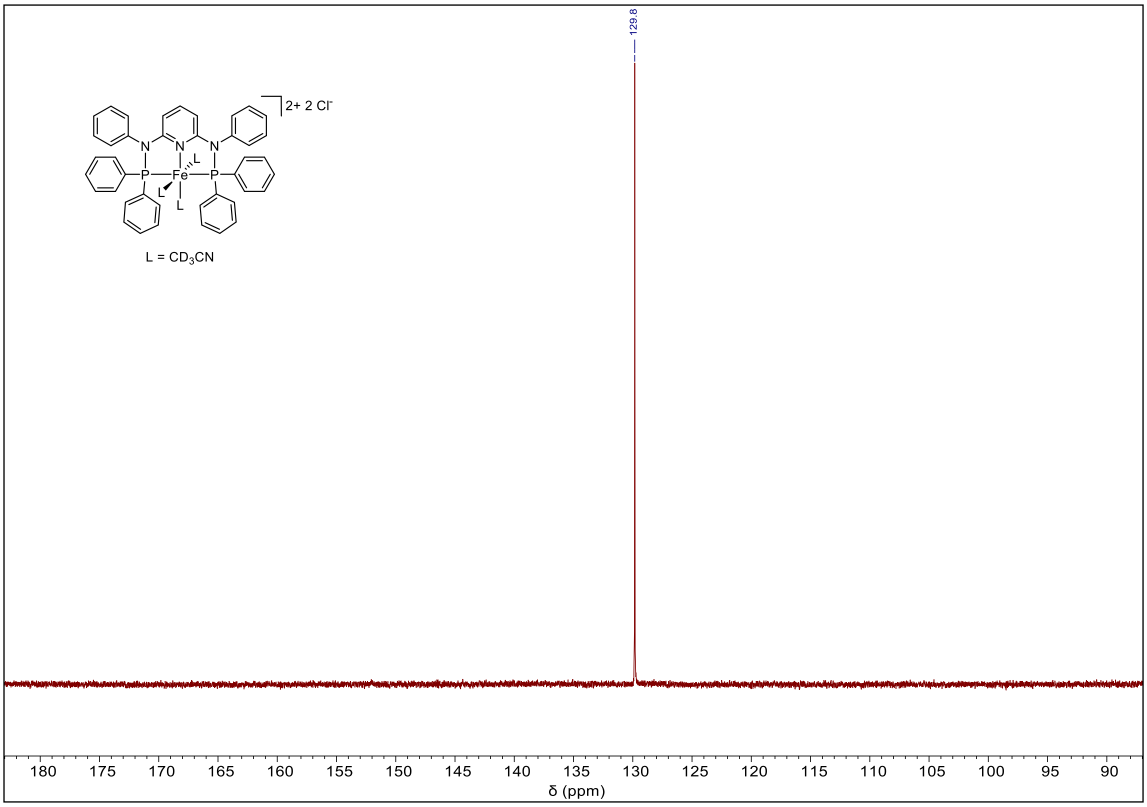

Figure S10. $^{31}P\{^1H\}$ NMR spectrum of **Fe$_{Cl}$** in CD_3CN at 202 MHz and 296 K.

1.1.4. $[FeL(MeCN)_3](OTf)_2$

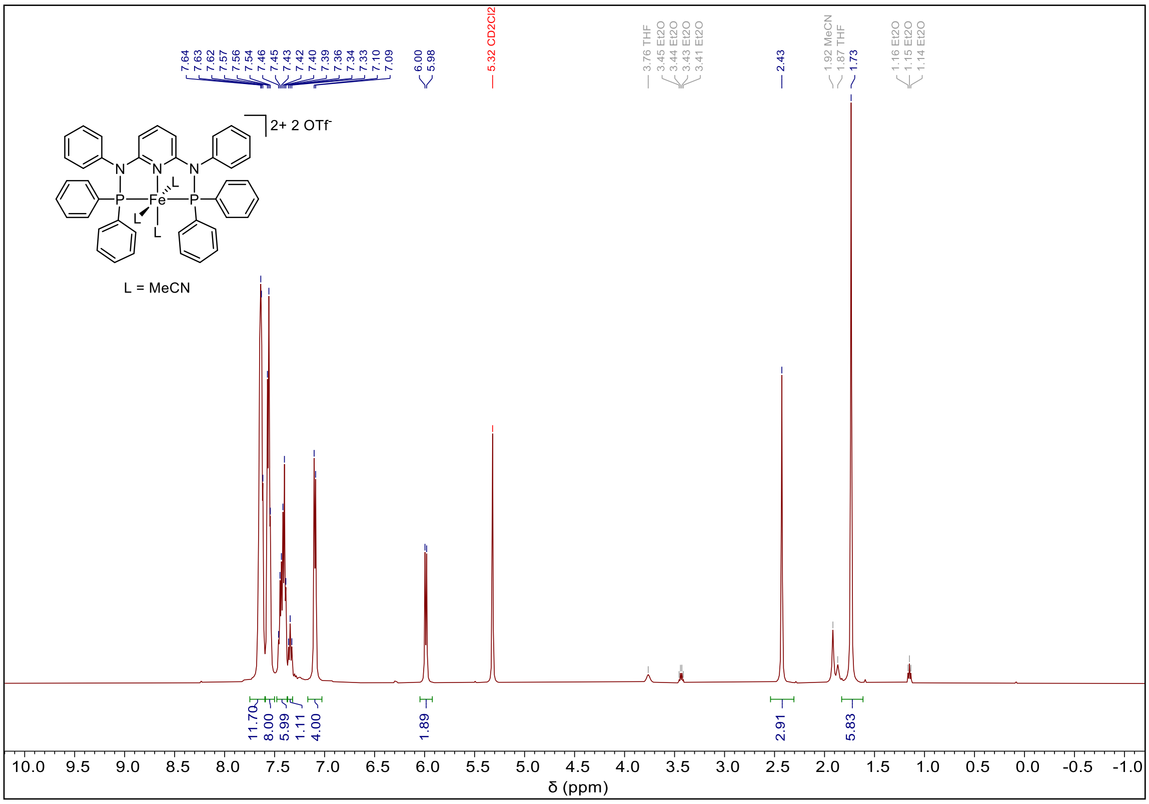

Figure S11. ^{1}H NMR spectrum of **Fe_{MeCN}** in CD_2Cl_2 at 500 MHz and 296 K.

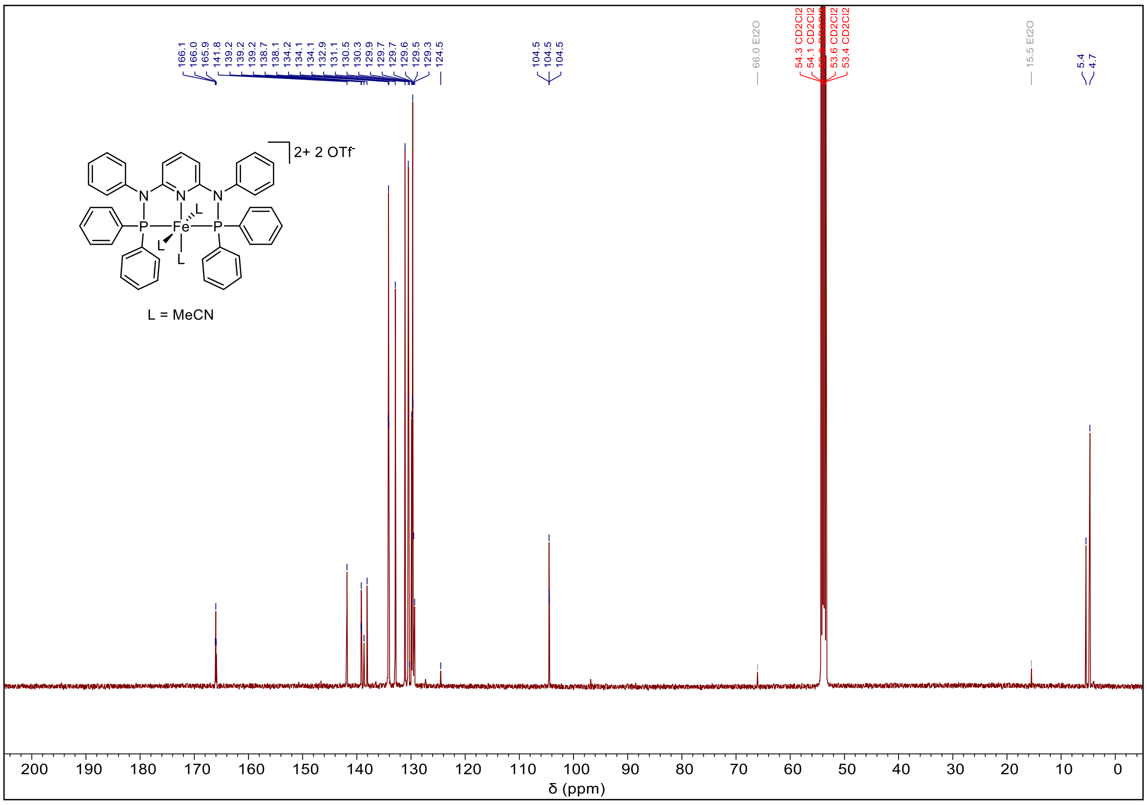

Figure S12. $^{13}C\{^{1}H\}$ NMR spectrum of **Fe_{MeCN}** in CD_2Cl_2 at 126 MHz and 296 K.

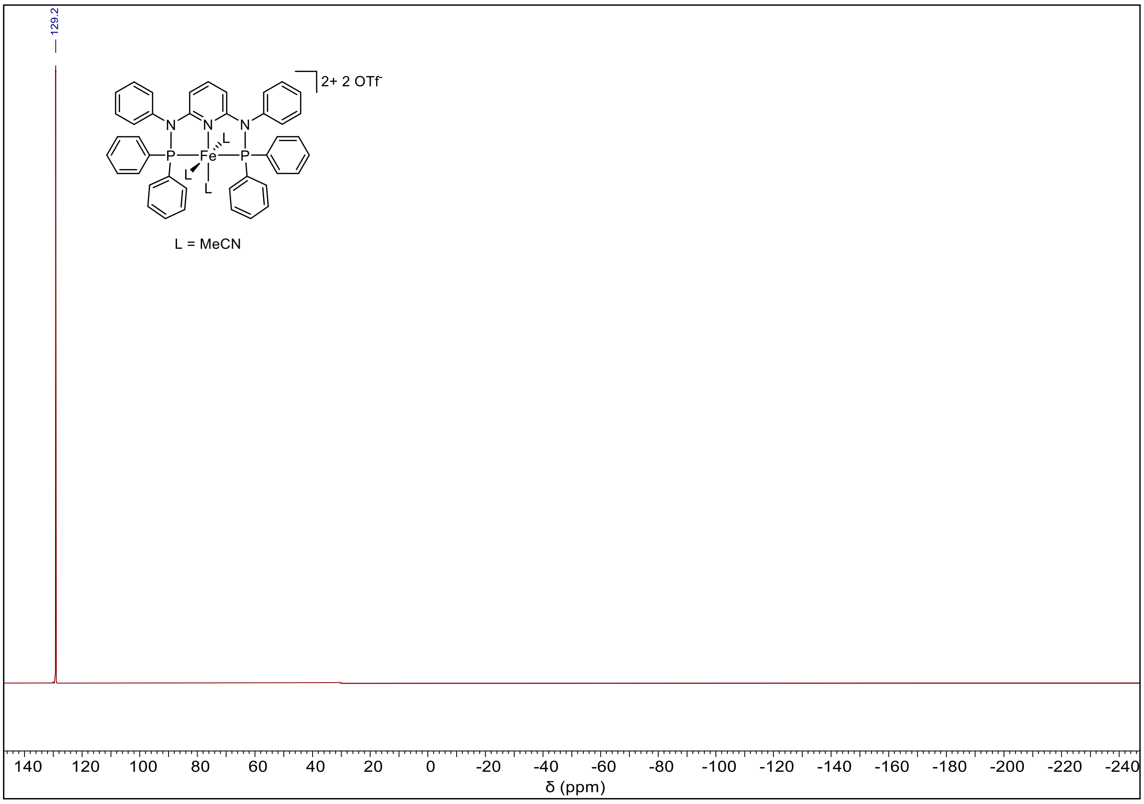

Figure S13. $^{31}P\{^{1}H\}$ NMR spectrum of **Fe$_{MeCN}$** in CD_2Cl_2 at 202 MHz and 296 K.

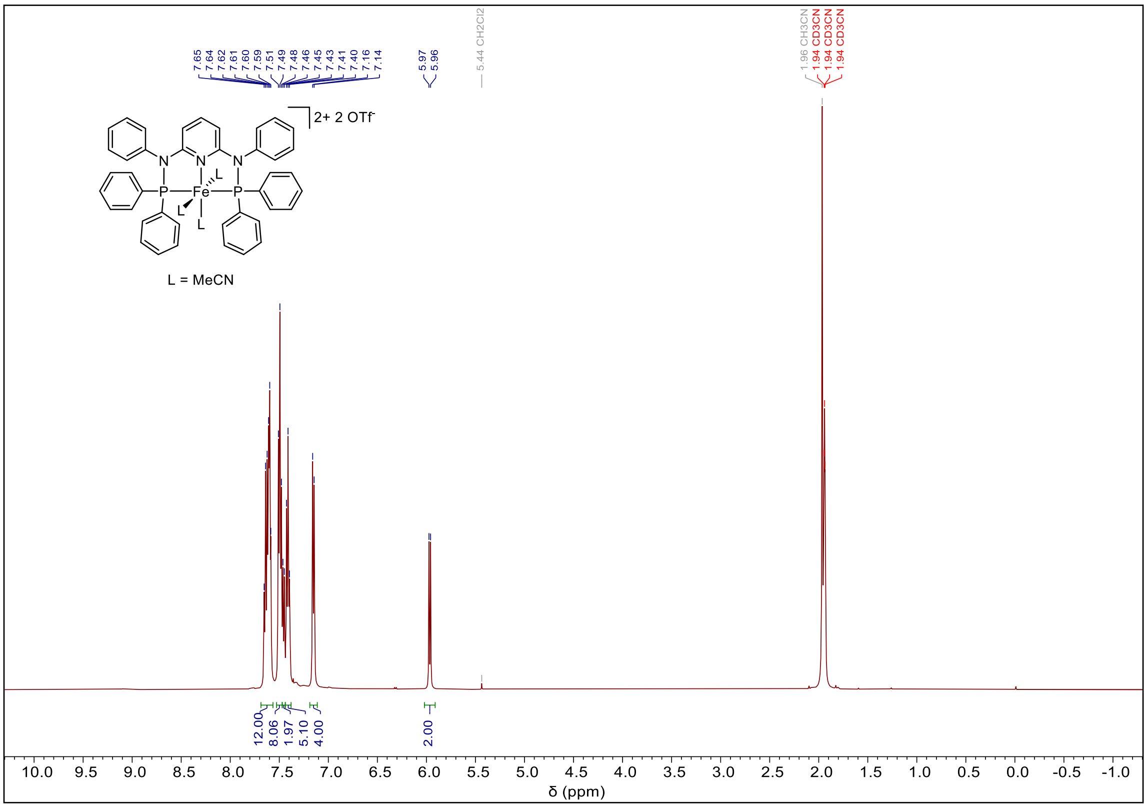

Figure S14. ^{1}H NMR spectrum of **Fe$_{MeCN}$** in CD_3CN at 500 MHz and 296 K.

1.1.5. [CoLCl$_2$]

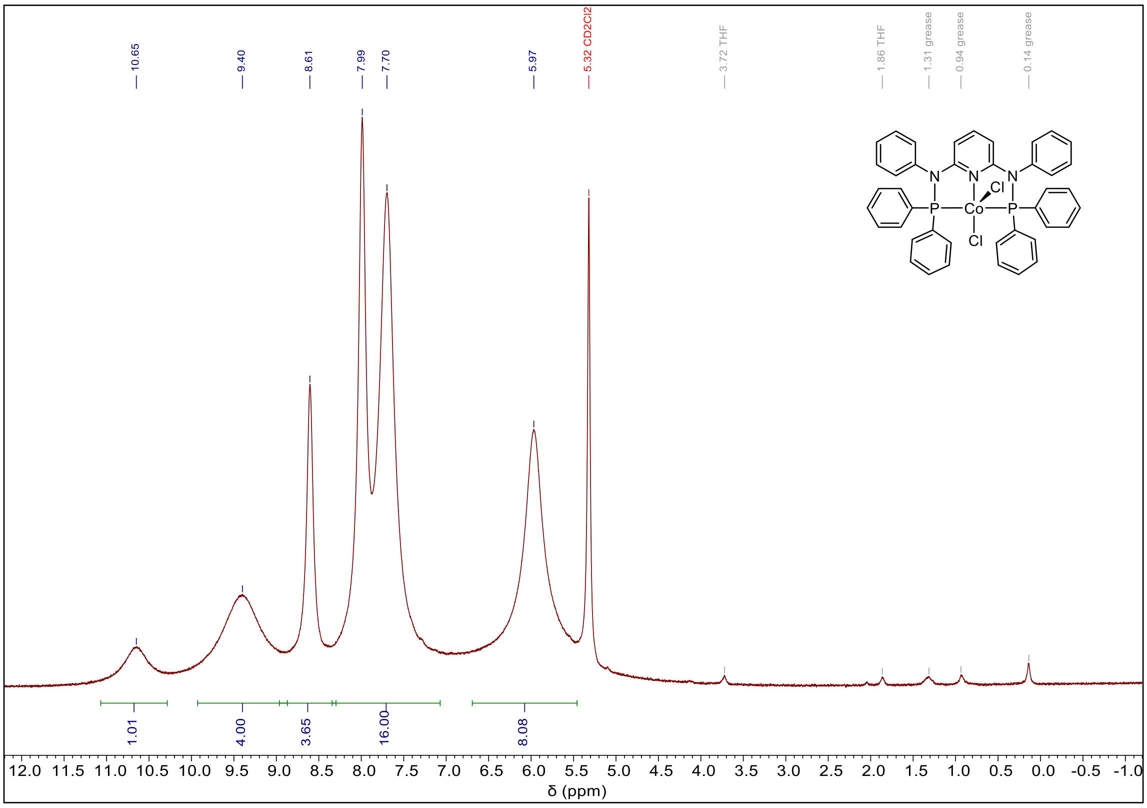

Figure S15. ^{1}H NMR spectrum of **Co$_{Cl}$** in CD_2Cl_2 at 400 MHz and 296 K.

1.1.6. [NiLCl$_2$]

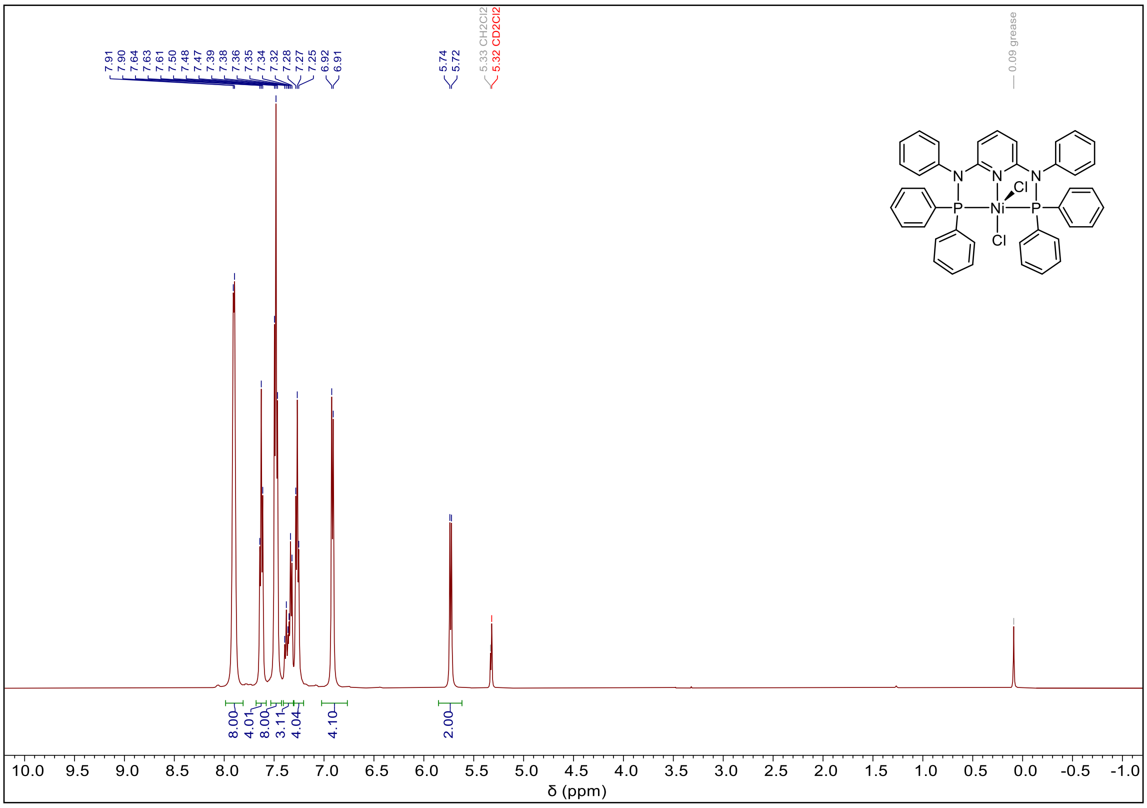

Figure S16. ^{1}H NMR spectrum of **Ni$_{Cl}$** in CD_2Cl_2 at 500 MHz and 296 K.

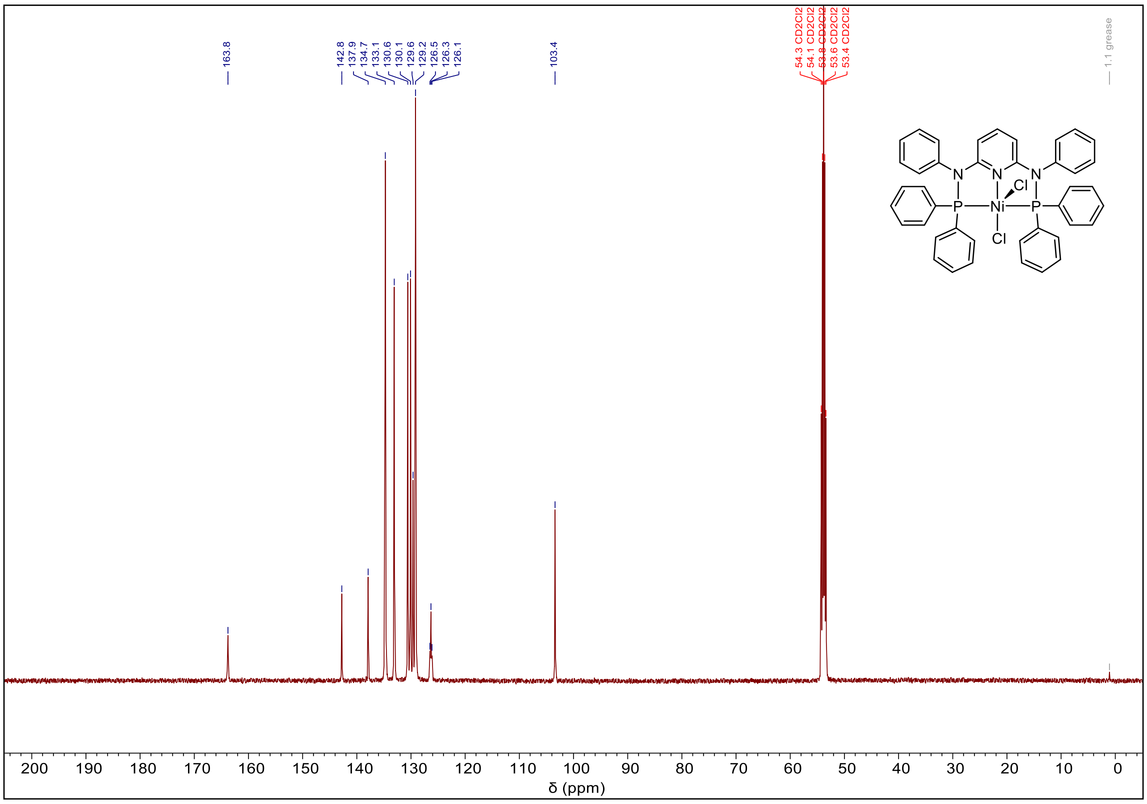

Figure S17. ^{13}C{^{1}H} NMR spectrum of **Ni$_{Cl}$** in CD_2Cl_2 at 126 MHz and 296 K.

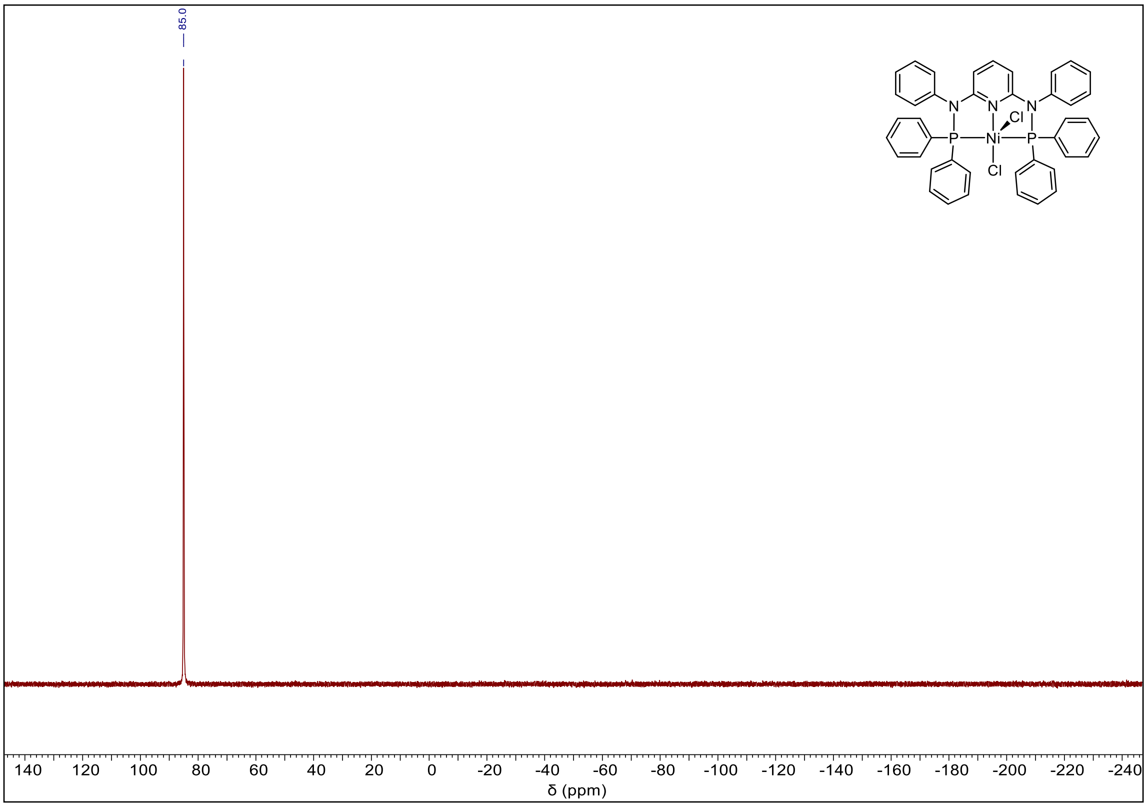

Figure S18. $^{31}P\{^{1}H\}$ NMR spectrum of $\mathbf{Ni_{Cl}}$ in CD_2Cl_2 at 202 MHz and 296 K.

1.1.7. [CuLCl]

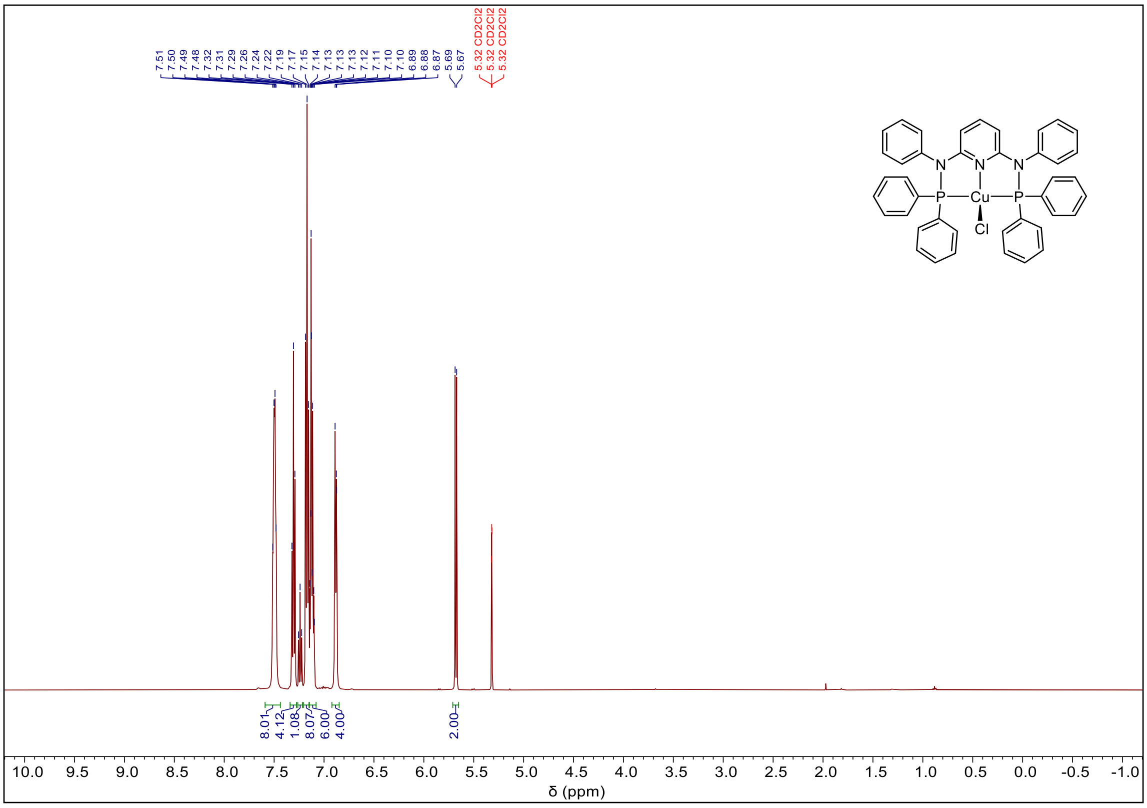

Figure S19. ^{1}H NMR spectrum of **Cu$_{Cl}$** in CD_2Cl_2 at 500 MHz and 296 K.

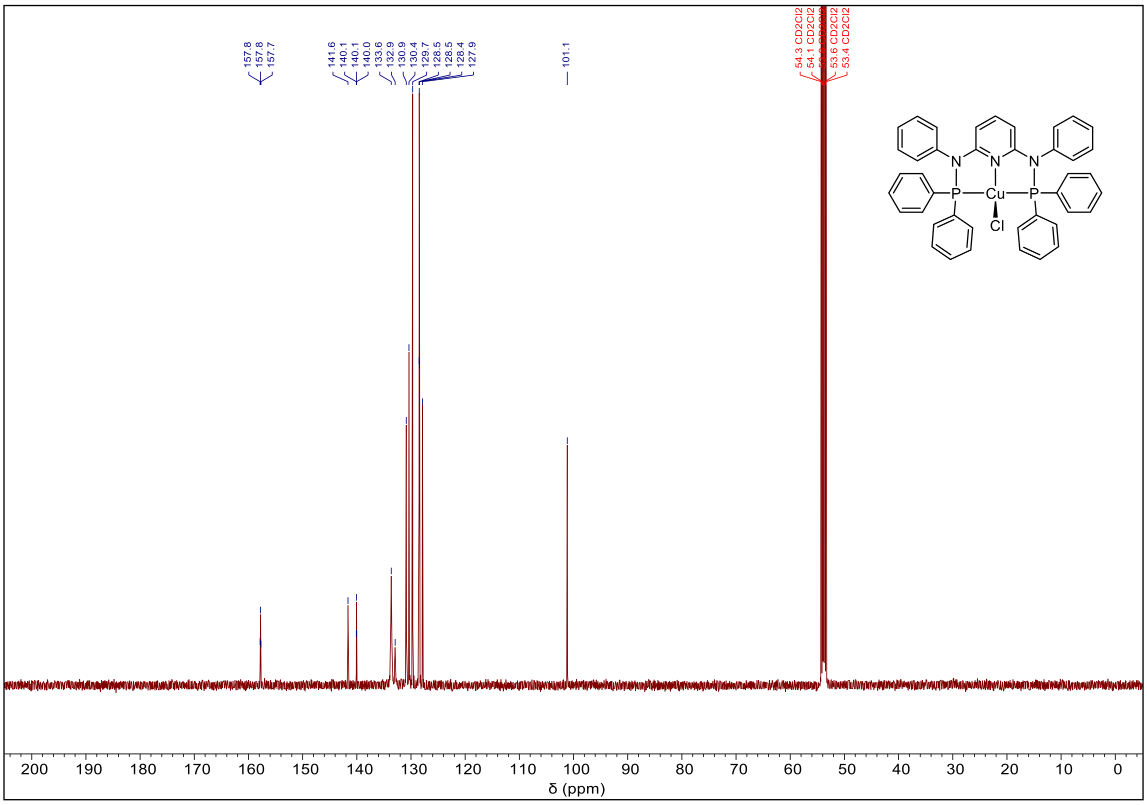

Figure S20. $^{13}C\{^{1}H\}$ NMR spectrum of **Cu$_{Cl}$** in CD_2Cl_2 at 126 MHz and 296 K.

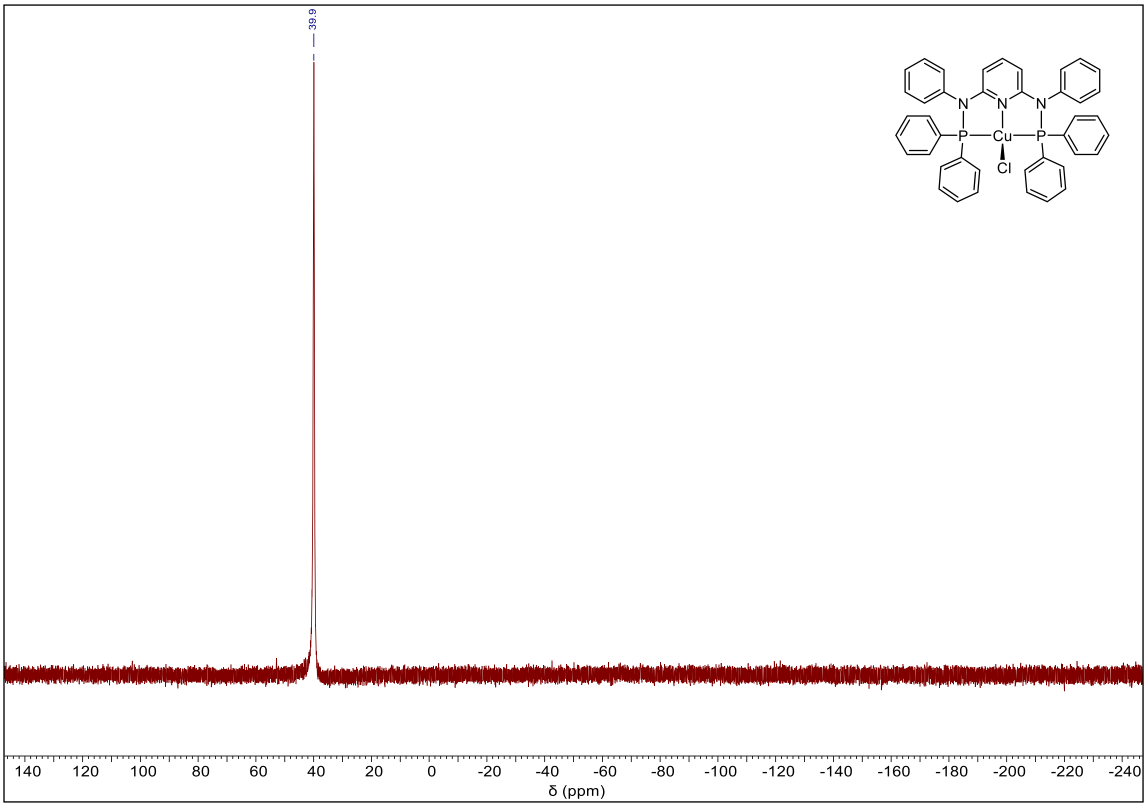

Figure S21. $^{31}P\{^{1}H\}$ NMR spectrum of **Cu**$_{\mathbf{Cl}}$ in CD_2Cl_2 at 202 MHz and 296 K.

1.1.8. [CuLI]

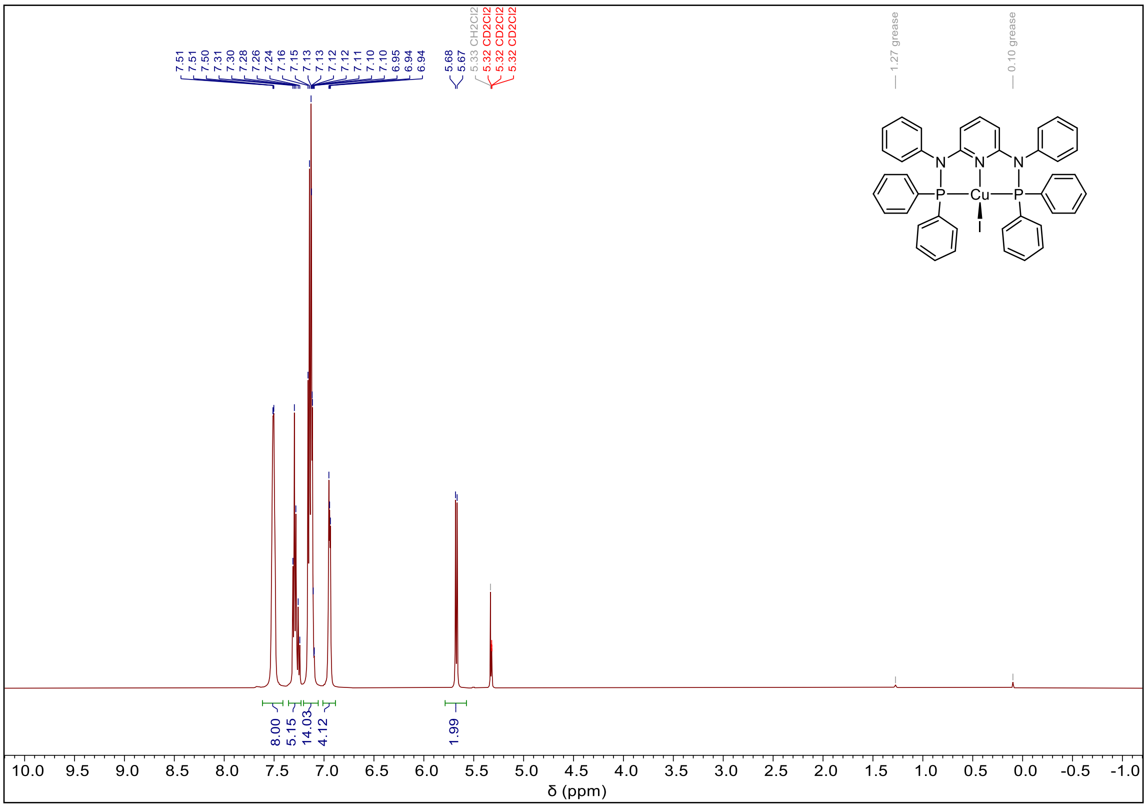

Figure S22. ^{1}H NMR spectrum of **Cu$_I$** in CD_2Cl_2 at 500 MHz and 296 K.

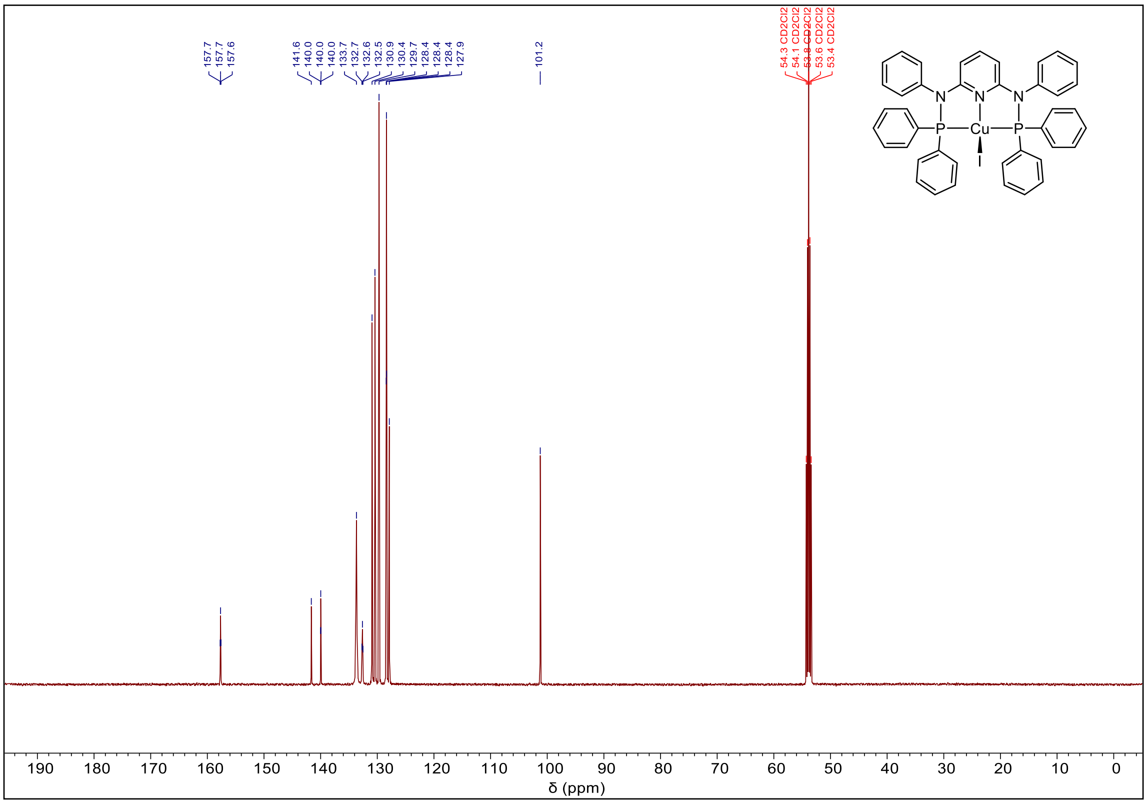

Figure S23. $^{13}C\{^{1}H\}$ NMR spectrum of **Cu$_I$** in CD_2Cl_2 at 126 MHz and 296 K.

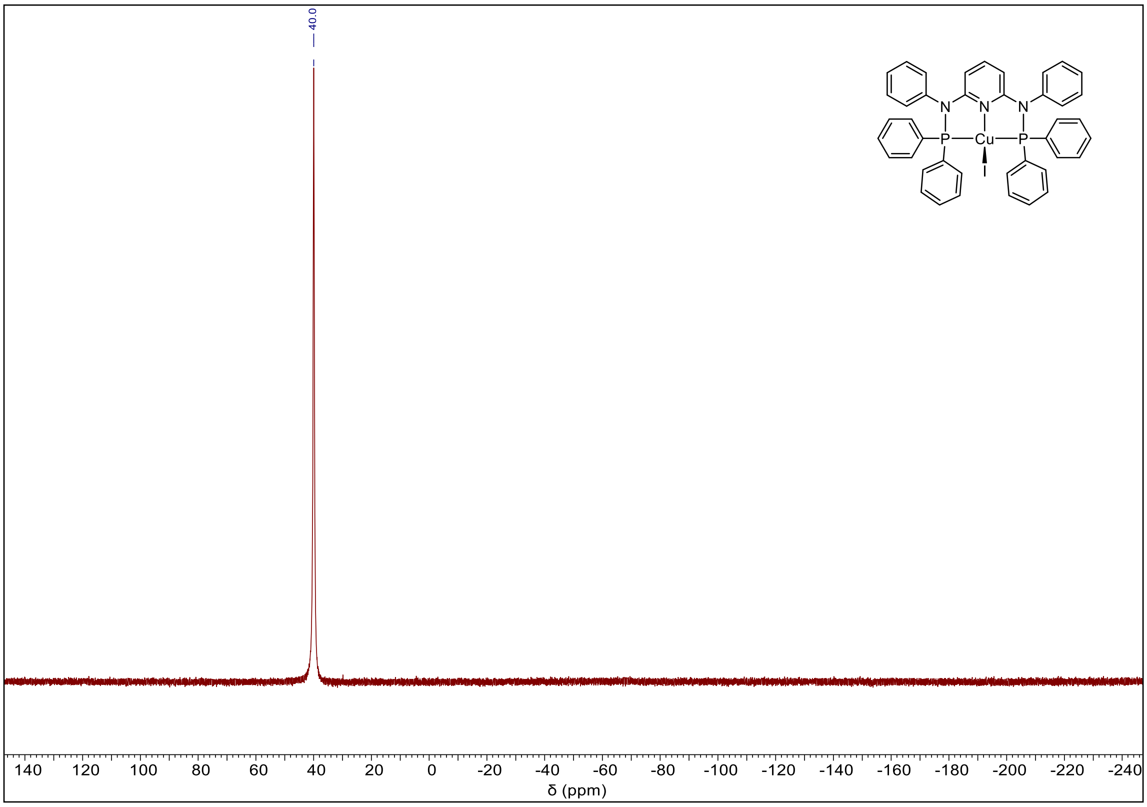

Figure S24. $^{31}P\{^{1}H\}$ NMR spectrum of **CuI** in CD_2Cl_2 at 202 MHz and 296 K.

1.1.9. [ZnLCl₂]

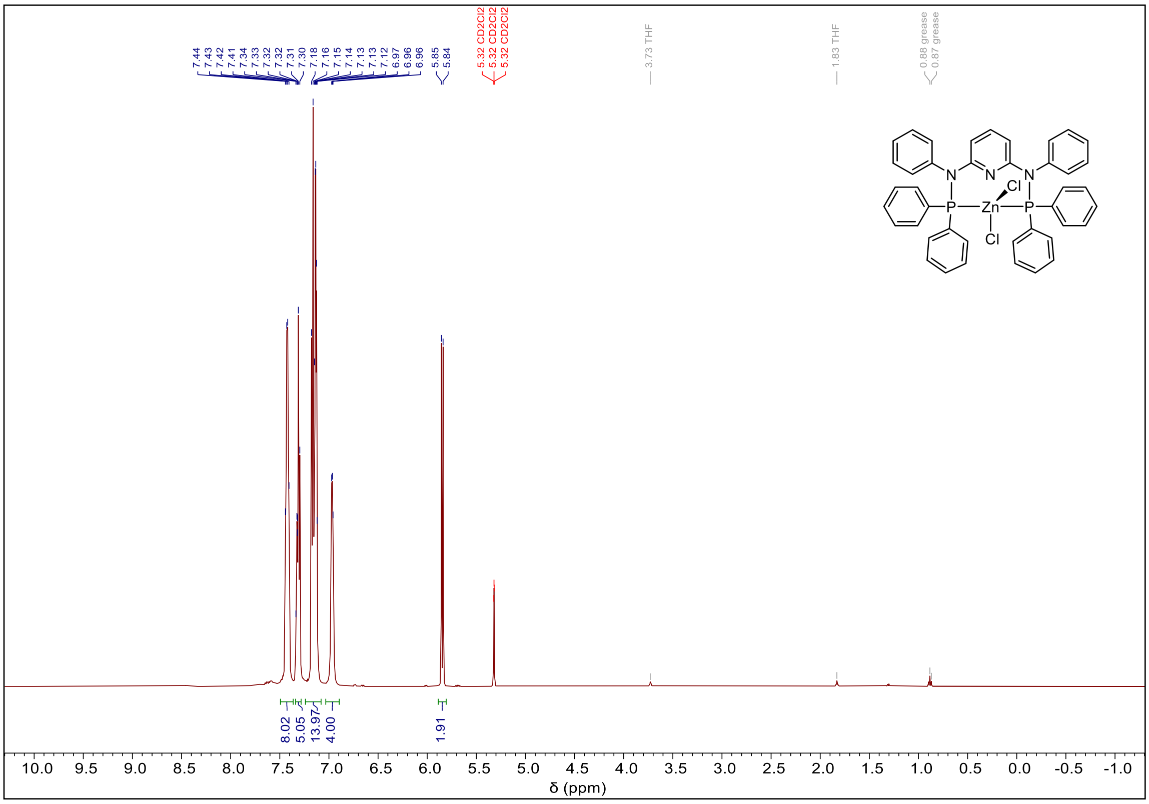

Figure S25. ^{1}H NMR spectrum of **Zn_{Cl}** in CD_2Cl_2 at 500 MHz and 296 K.

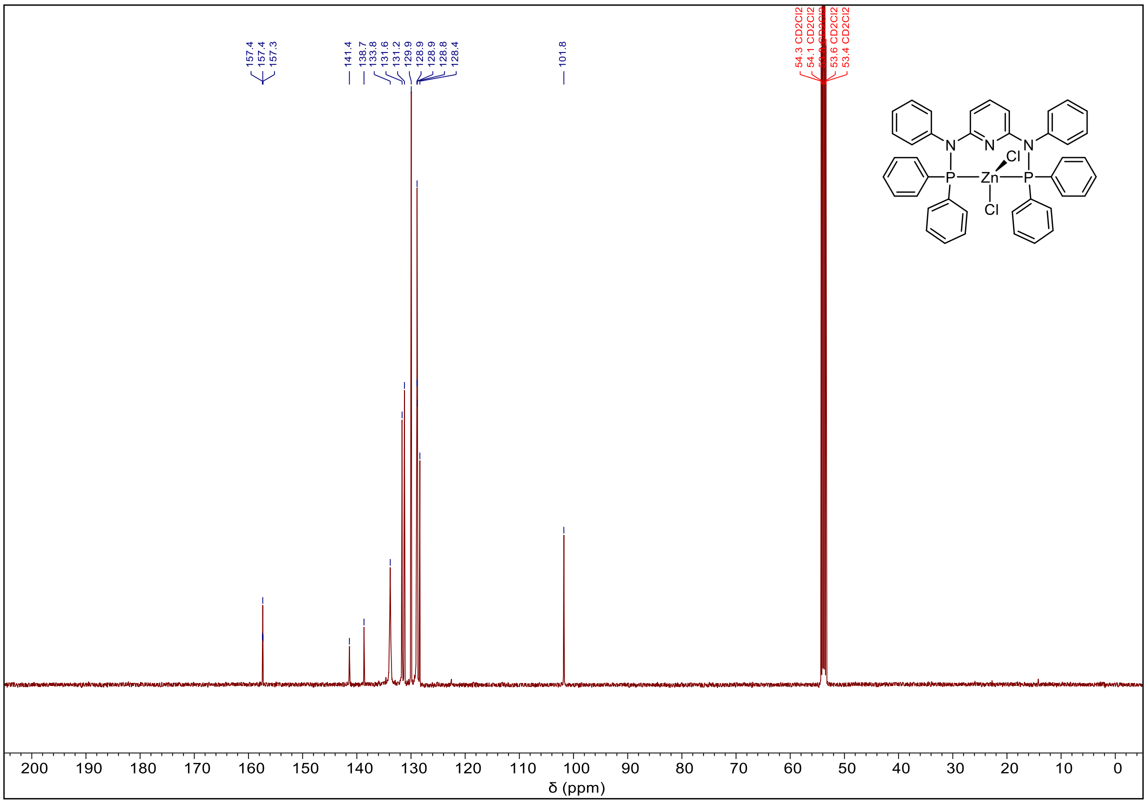

Figure S26. $^{13}C\{^1H\}$ NMR spectrum of **Zn**$_{Cl}$ in CD_2Cl_2 at 126 MHz and 296 K.

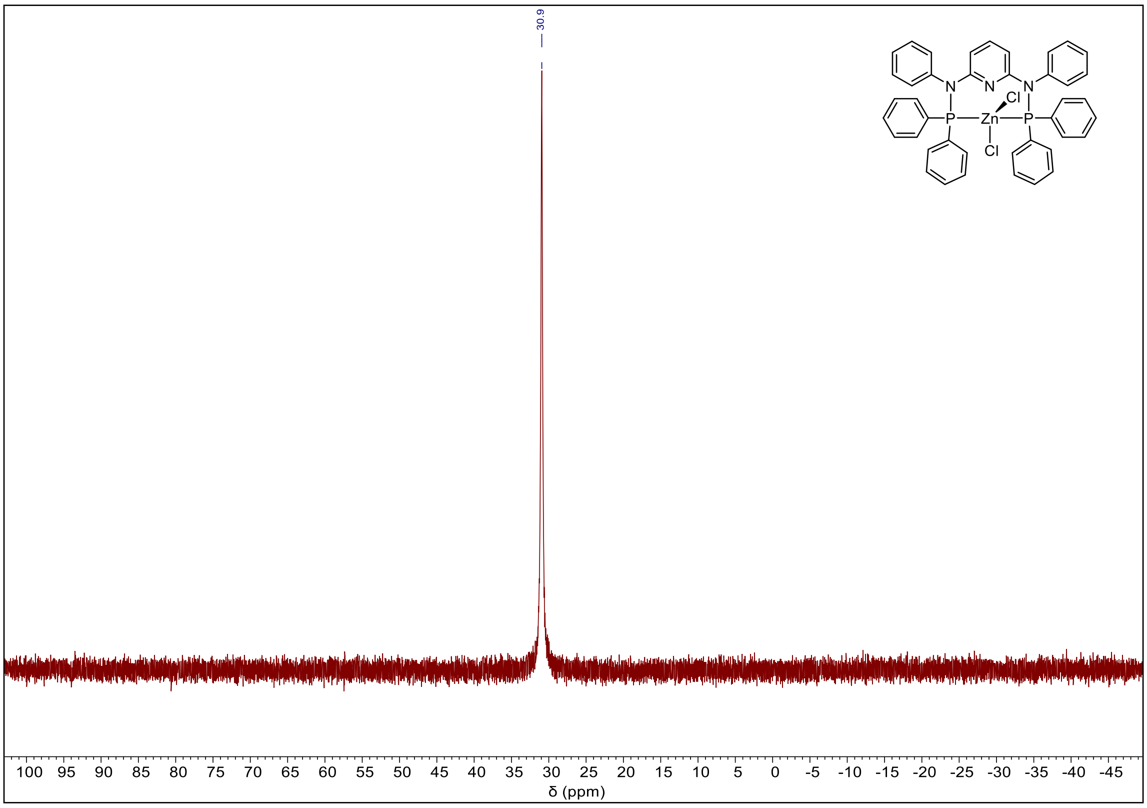

Figure S27. $^{31}P\{^1H\}$ NMR spectrum of **Zn**$_{Cl}$ in CD_2Cl_2 at 202 MHz and 296 K.

1.1.10. [ZnL(OTf)$_2$]

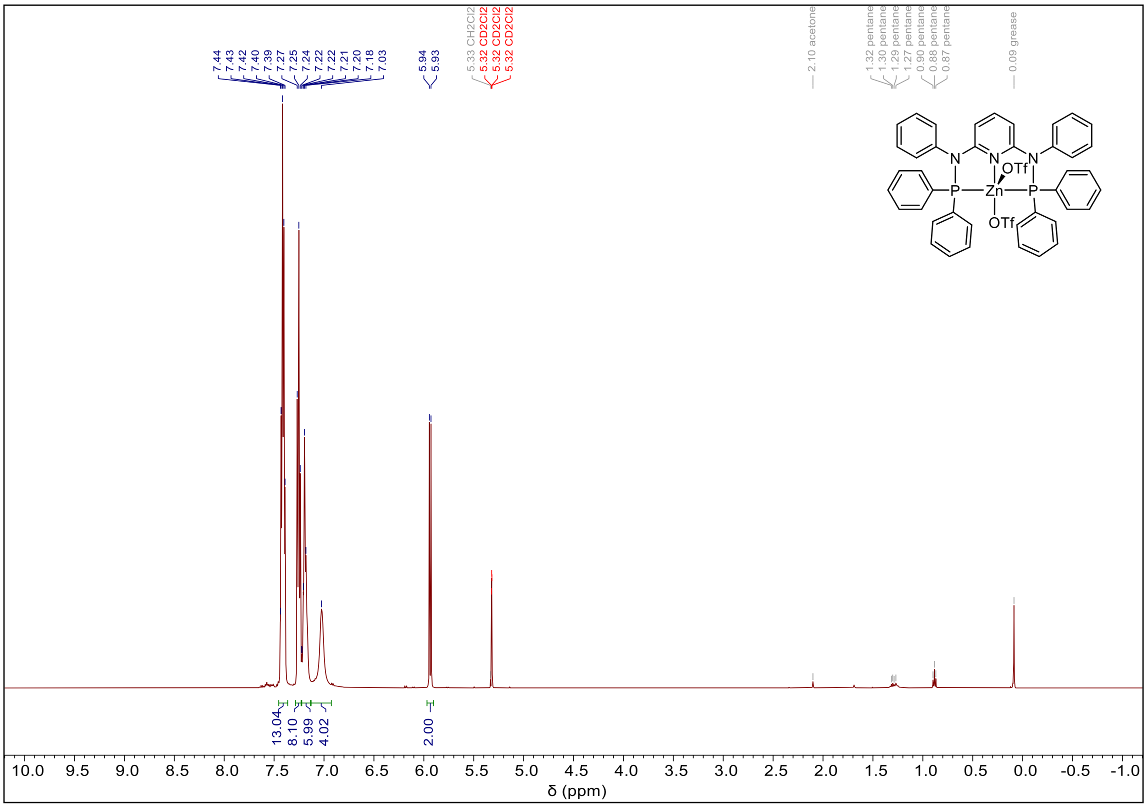

Figure S28. ^{1}H NMR spectrum of **Zn$_{OTf}$** in CD_2Cl_2 at 500 MHz and 296 K.

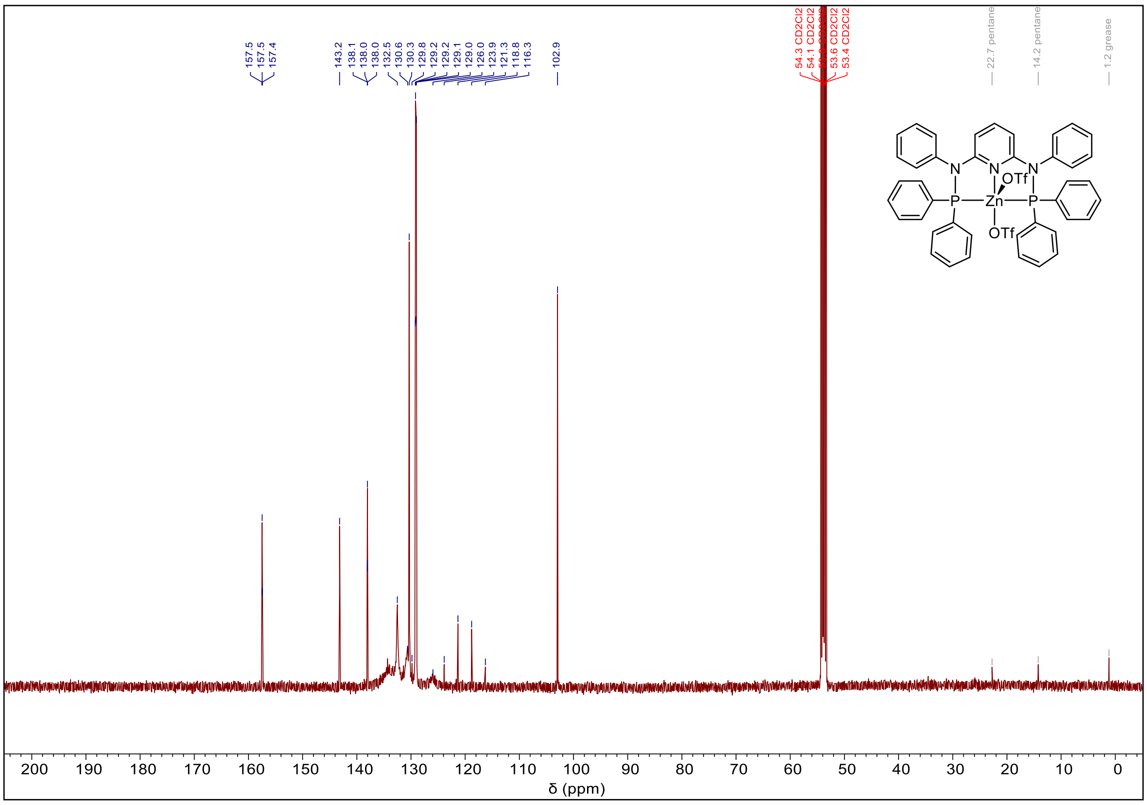

Figure S29. $^{13}C\{^{1}H\}$ NMR spectrum of **Zn$_{OTf}$** in CD_2Cl_2 at 126 MHz and 296 K.

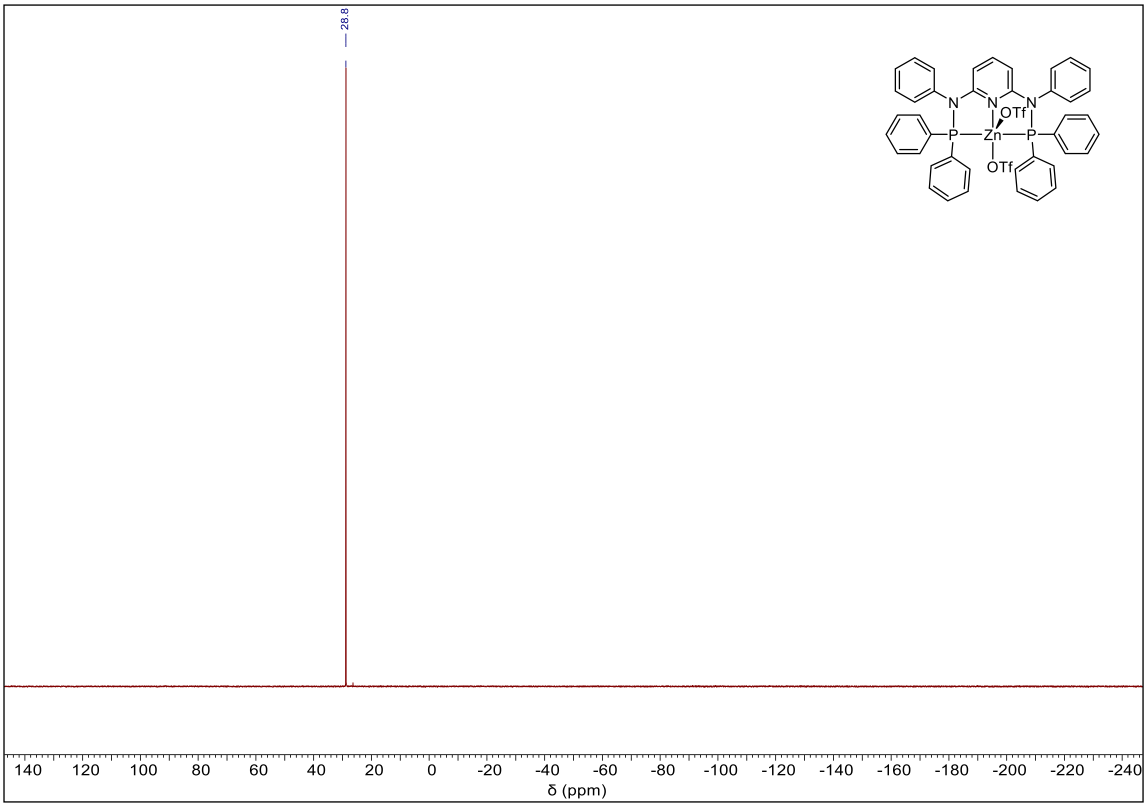

Figure S30. $^{31}P\{^{1}H\}$ NMR spectrum of **Zn$_{OTf}$** in CD_2Cl_2 at 202 MHz and 296 K.

1.1.11. Coordination Chemical Shift

The ^{31}P coordination NMR chemical shift $\Delta\delta$ can be calculated from the difference of the ^{31}P NMR chemical shifts of the phosphine in the **M$_X$** complexes (δ_P(**M$_X$**)) and the free ligand **L** (δ_P(**L**)) according to eq.(1).[1]

$$\Delta\delta = \delta_P(\mathbf{M_X}) - \delta_P(\mathbf{L}) \qquad (1)$$

1.2. UV/VIS

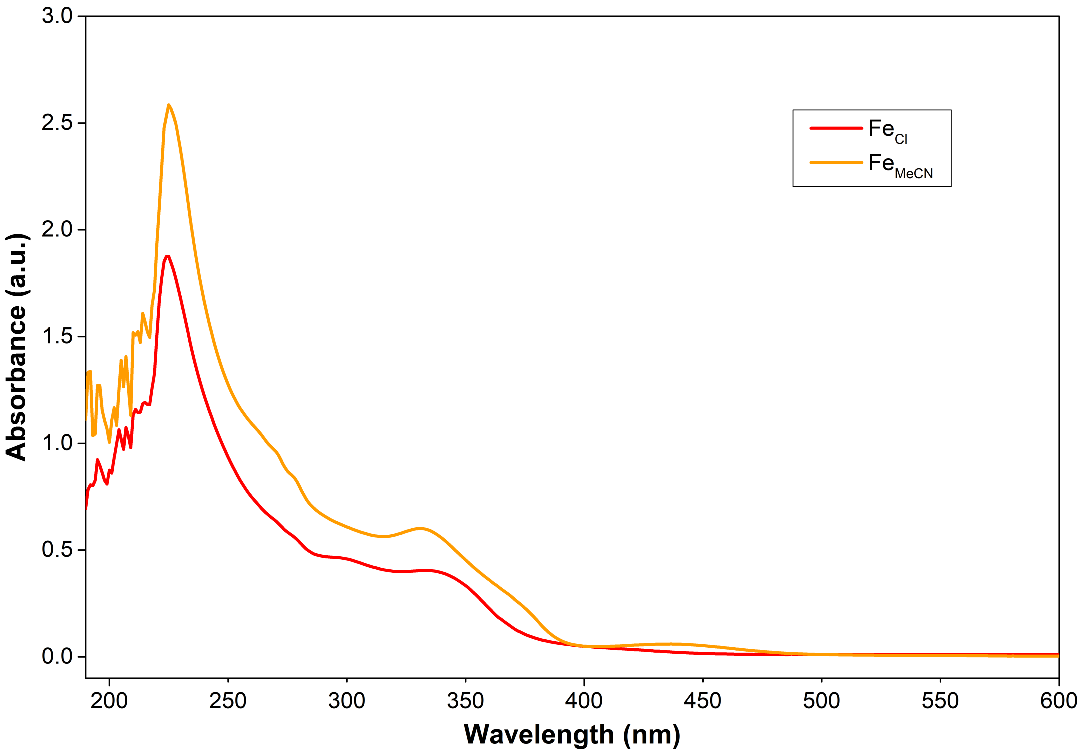

Figure S31. UV/VIS spectra of 0.25 mM solutions of **Fe$_{Cl}$** and **Fe$_{MeCN}$** in CH_2Cl_2.

The absorption bands at 225 nm in the UV/VIS spectra of **Fe$_{Cl}$** and **Fe$_{MeCN}$** point to a transition centered on the aromatic ligand backbone. The band at 335 nm for **Fe$_{Cl}$** shows a slight hypsochromic shift to 331 nm for **Fe$_{MeCN}$**. For the latter complex, an additional broad band is detected at 440 nm.

1.3. Mössbauer

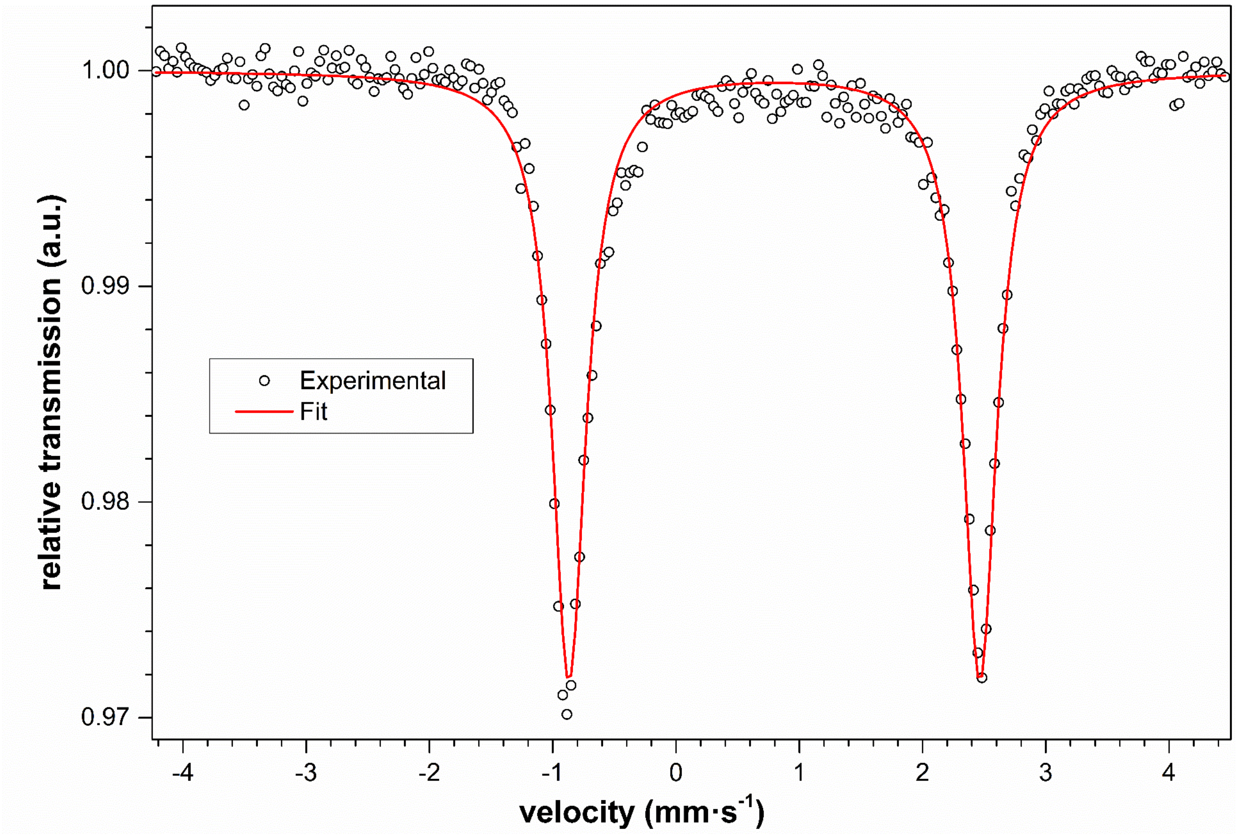

Figure S32. Zero-field ^{57}Fe-Mössbauer spectrum of solid **Fe$_{Cl}$** recorded at 80.00 K. The red line represents a fit with a Lorentzian doublet with isomer shift δ = 0.80 mm·s^{-1}, quadrupole splitting ΔE_Q = 3.33 mm·s^{-1}, and line width (full width at half maximum) Γ = 0.33 mm·s^{-1} at 100% relative intensity.

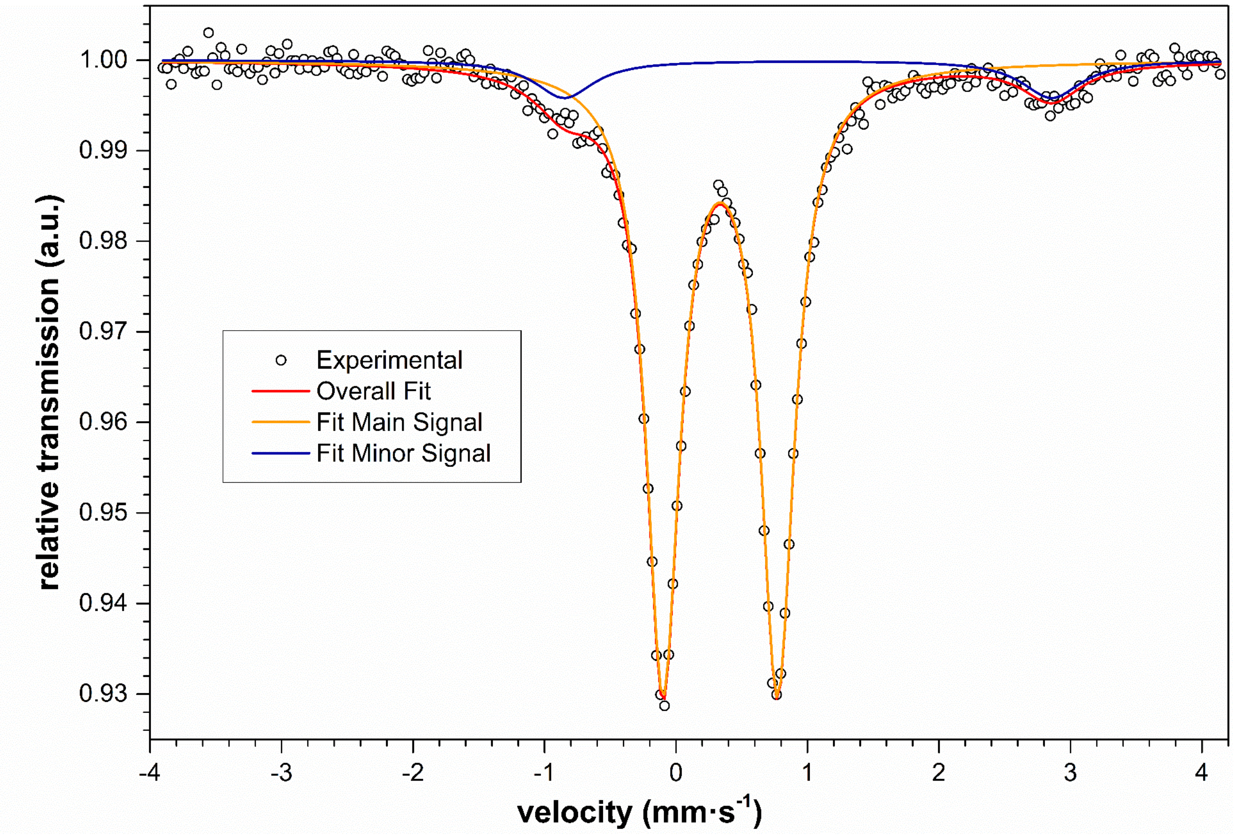

Figure S33. Zero-field ^{57}Fe-Mössbauer spectrum of solid $\mathbf{Fe_{MeCN}}$ recorded at 80.00 K. The red line represents a fit with two Lorentzian doublet with isomer shift. Main component (orange): δ = 0.34 mm·s^{-1}, ΔE_Q = 0.87 mm·s^{-1}, Γ = 0.31 mm·s^{-1}, 91% relative intensity. Minor component (blue): δ = 1.00 mm·s^{-1}, ΔE_Q = 3.71 mm·s^{-1}, Γ = 0.55 mm·s^{-1}, 9% relative intensity.

1.4. Electron Paramagnetic Resonance (EPR)

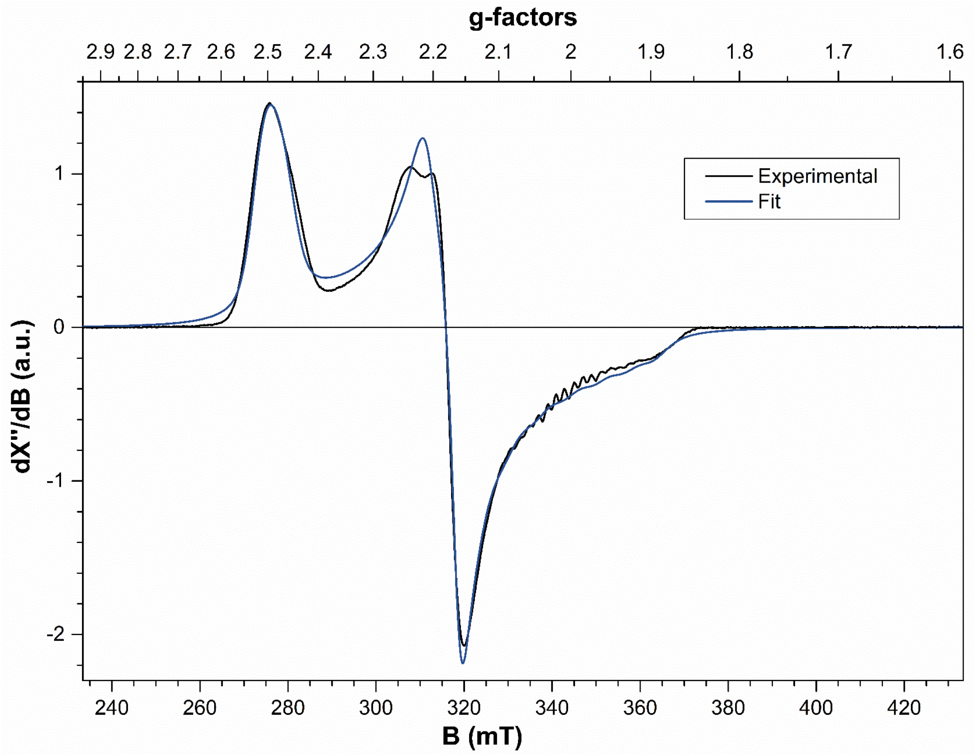

Figure S34. X-band EPR spectrum of ca. 1 mM **Co$_{Cl}$** in 2-MTHF recorded at 10 K; microwave frequency 9.63715 GHz, power 0.2 mW, modulation 0.8 mT/100 kHz. The blue line is a simulation with $g_{z,y,x}$ = (2.485, 2.165, 2.030) and first-order ^{59}Co hyperfine splitting with $A_{x,y,z}$ = (63.44, 2.36, 11.89) 10^{-4} cm^{-1}.

1.5. Magnetic Susceptibility Measurements

1.5.1. [$FeLCl_2$]

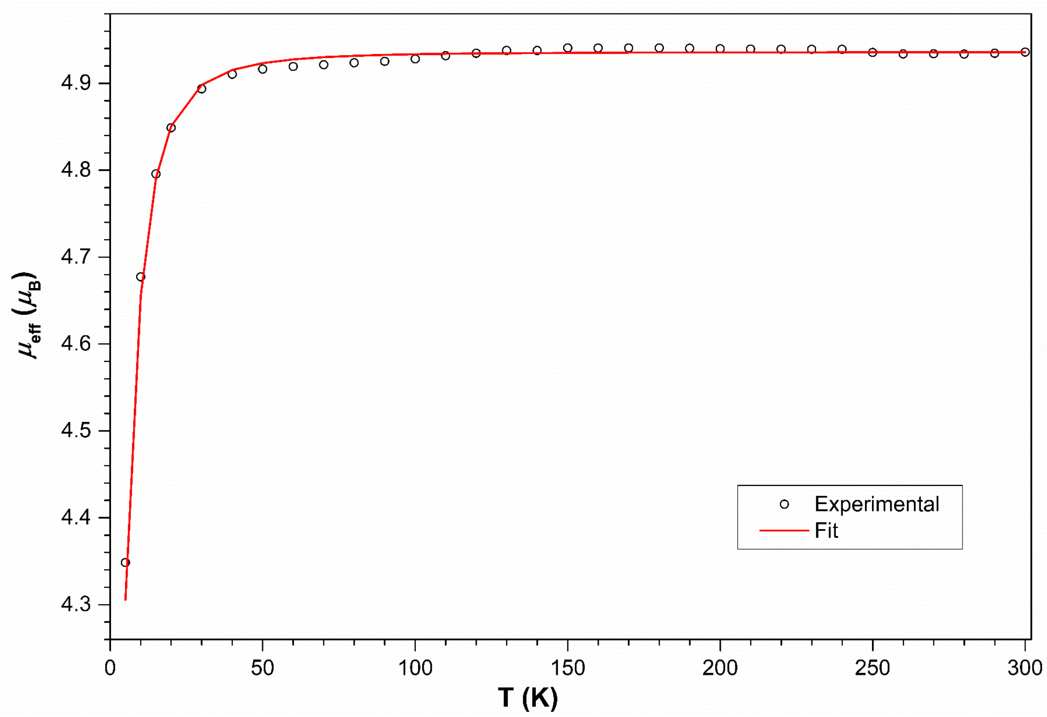

Figure S35. Effective magnetic moment of a solid powder sample of $\mathbf{Fe_{Cl}}$, μ_{eff} vs. temperature T recorded with B = 1 T field. The data were corrected for diamagnetic contribution of χ_{dia} = –375·10^{-6} emu. The solid line is a spin Hamiltonian simulation with S = 2, D = 4.27 cm^{-1}, $g_{average}$ = 2.021.

1.5.2. [CoLCl2]

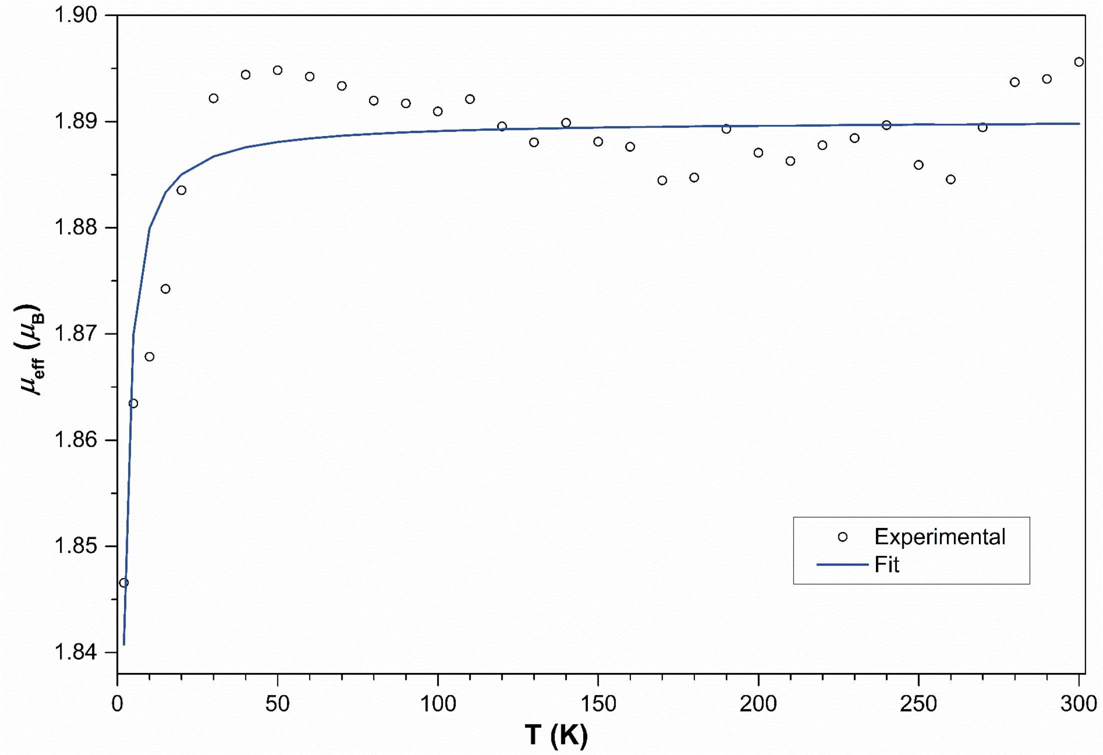

Figure S36. Effective magnetic moment of a solid powder sample of **Fe_{Cl}**, μ_{eff} vs. temperature T recorded with B = 1 T field. The data were corrected for diamagnetic contribution of χ_{dia} = –360·10^{-6} emu. The solid line is a spin Hamiltonian simulation with S = ½, $g_{average}$ = 2.183, intermolecular coupling (2zJ)= –4.3 cm^{-1}.

1.6. X-ray Diffraction

1.6.1. Structures

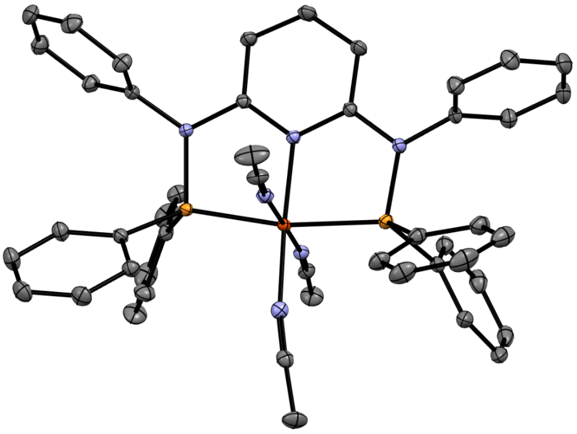

Figure S37. Molecular structure of **$[FeL(MeCN)_3](Cl_3FeOFeCl_3)$** received from crystals obtained from a concentrated MeCN/Et_2O (3:1) solution at –30 °C under air. H-atoms and outer-sphere ligands were omitted for clarity, thermal ellipsoids are shown at 50% probability level.

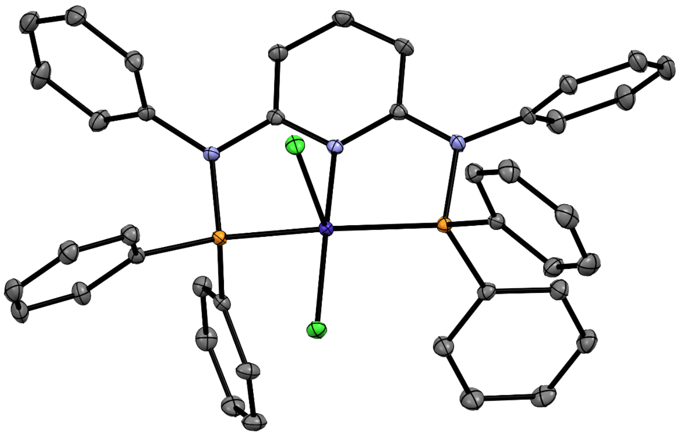

Figure S38. Molecular structure of **Co_{Cl}** received from crystals obtained from a concentrated MeCN/Et_2O (3:1) solution at –35 °C. H-atoms and solvent molecules were omitted for clarity, thermal ellipsoids are shown at 50% probability level.

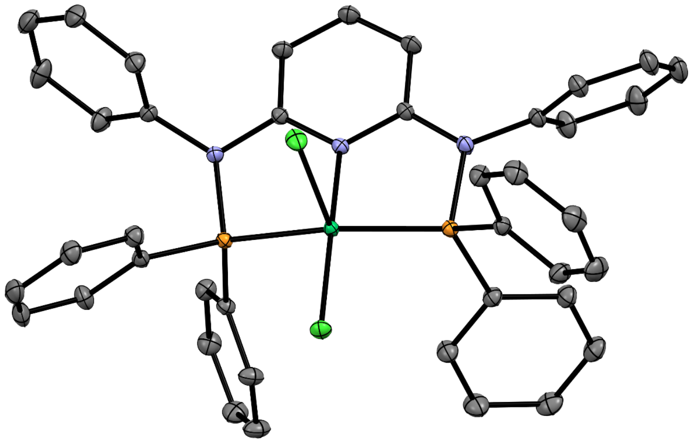

Figure S39. Molecular structure of **Ni_{Cl}** received from crystals obtained from a concentrated MeCN/Et_2O (3:1) solution at –35 °C. H-atoms and solvent molecules were omitted for clarity, thermal ellipsoids are shown at 50% probability level.

1.6.2. Data and Refinements

Table S1. Crystal data and structure refinement for **L**.

CCDC No.	2109424	
Identification code	CEC0014_1	
Empirical formula	$C_{41}H_{33}N_3P_2$	
Formula weight	629.64	
Temperature	100(2) K	
Wavelength	0.71073 Å	
Crystal system	Orthorhombic	
Space group	Pbcn; No 60	
Unit cell dimensions	a = 34.0981(10) Å	α = 90°.
	b = 8.7711(2) Å	β = 90°.
	c = 23.9656(8) Å	γ = 90°.
Volume	7167.6(4) $Å^3$	
Z	8	
Density (calculated)	1.167 Mg/m^3	
Absorption coefficient	0.153 mm^{-1}	
F(000)	2640	
Crystal size	0.370 x 0.150 x 0.136 mm^3	
Theta range for data collection	1.801 to 32.499°.	
Index ranges	-51<=h<=51, -13<=k<=13, -36<=l<=36	
Reflections collected	137542	
Independent reflections	12975 [R(int) = 0.0426]	
Completeness to theta = 25.242°	100.0 %	
Absorption correction	Gaussian	
Max. and min. transmission	0.838 and 0.789	
Refinement method	Full-matrix least-squares on F^2	
Data / restraints / parameters	12975 / 72 / 415	
Goodness-of-fit on F^2	1.173	
Final R indices [I>2sigma(I)]	R_1 = 0.0604, wR_2 = 0.1376	
R indices (all data)	R_1 = 0.0660, wR_2 = 0.1407	
Extinction coefficient	n/a	
Largest diff. peak and hole	0.544 and -0.436 e.$Å^{-3}$	

Table S2. Crystal data and structure refinement for **Mn_{Br}**.

CCDC No.	2109428	
Identification code	CEC0038	
Empirical formula	$C_{43}H_{33}BrMnN_3O_2P_2$	
Formula weight	820.51	
Temperature	100(2) K	
Wavelength	0.71073 Å	
Crystal system	Monoclinic	
Space group	$P2_1/c$; No. 14	
Unit cell dimensions	a = 11.6847(9) Å	α = 90°.
	b = 17.1633(13) Å	β = 104.993(3)°.
	c = 18.5524(14) Å	γ = 90°.
Volume	3594.0(5) $Å^3$	
Z	4	
Density (calculated)	1.516 Mg/m^3	
Absorption coefficient	1.611 mm^{-1}	
F(000)	1672	
Crystal size	0.20 x 0.20 x 0.15 mm^3	
Theta range for data collection	2.160 to 40.274°.	
Index ranges	-21<=h<=21, -31<=k<=31, -32<=l<=33	
Reflections collected	165160	
Independent reflections	22584 [R(int) = 0.0866]	
Completeness to theta = 25.242°	99.9 %	
Absorption correction	Semi-empirical from equivalents	
Max. and min. transmission	0.7915 and 0.6979	
Refinement method	Full-matrix least-squares on F^2	
Data / restraints / parameters	22584 / 1 / 472	
Goodness-of-fit on F^2	1.205	
Final R indices [I>2sigma(I)]	R_1 = 0.0568, wR_2 = 0.1224	
R indices (all data)	R_1 = 0.0732, wR_2 = 0.1267	
Extinction coefficient	n/a	
Largest diff. peak and hole	1.473 and -1.618 e.$Å^{-3}$	

Table S3. Crystal data and structure refinement for **Fe_{Cl}**.

CCDC No.	2109421	
Identification code	CEC0020	
Empirical formula	$C_{42}H_{35}Cl_4FeN_3P_2$	
Formula weight	841.32	
Temperature	100(2) K	
Wavelength	0.71073 Å	
Crystal system	Monoclinic	
Space group	$P2_1/n$; No. 14	
Unit cell dimensions	a = 14.2736(6) Å	α = 90°.
	b = 19.0907(9) Å	β = 112.610(2)°.
	c = 15.2486(7) Å	γ = 90°.
Volume	3835.8(3) $Å^3$	
Z	4	
Density (calculated)	1.457 Mg/m^3	
Absorption coefficient	0.791 mm^{-1}	
F(000)	1728	
Crystal size	0.300 x 0.150 x 0.120 mm^3	
Theta range for data collection	1.797 to 34.999°.	
Index ranges	-23<=h<=20,-30<=k<=30,-24<=l<=21	
Reflections collected	65790	
Independent reflections	16859 [R(int) = 0.0836]	
Completeness to theta = 25.242°	99.9 %	
Absorption correction	Semi-empirical from equivalents	
Max. and min. transmission	0.91 and 0.83	
Refinement method	Full-matrix least-squares on F^2	
Data / restraints / parameters	16859 / 0 / 469	
Goodness-of-fit on F^2	1.059	
Final R indices [I>2sigma(I)]	R_1 = 0.0371, wR_2 = 0.0865	
R indices (all data)	R_1 = 0.0616, wR_2 = 0.0919	
Extinction coefficient	n/a	
Largest diff. peak and hole	0.749 and -0.521 e.$Å^{-3}$	

Table S4. Crystal data and structure refinement for **[Fe(PN$_3$P)(MeCN)$_3$](Cl$_3$FeOFeCl$_3$)**.

CCDC No.	2109425	
Identification code	CEC0018	
Chemical formula	$C_{47}H_{42}Cl_6Fe_3N_6OP_2$	
Formula weight	1149.05 g/mol	
Temperature	100(2) K	
Wavelength	0.71073 Å	
Crystal system	triclinic	
Space group	P -1	
Unit cell dimensions	a = 9.1344(5) Å	α = 85.506(2)°
	b = 12.1482(7) Å	β = 78.908(2)°
	c = 23.4429(14) Å	γ = 81.505(2)°
Volume	2521.4(3) Å^3	
Z	2	
Density (calculated)	1.513 g/cm^3	
Absorption coefficient	1.275 mm^{-1}	
F(000)	1168	
Theta range for data collection	1.87 to 32.50°	
Index ranges	-13<=h<=13, -18<=k<=18, -35<=l<=35	
Reflections collected	174213	
Independent reflections	18260 [R(int) = 0.0576]	
Absorption correction	Multi-Scan	
Max. and min. transmission	0.9050 and 0.7490	
Refinement method	Full-matrix least-squares on F^2	
Data / restraints / parameters	18260 / 0 / 589	
Goodness-of-fit on F^2	1.154	
Final R indices [I>2sigma(I)]	R_1 = 0.0451, wR_2 = 0.0968	
R indices (all data)	R_1 = 0.0536, wR_2 = 0.1000	
Largest diff. peak and hole	0.775 and -0.778 e.Å^{-3}	

Table S5. Crystal data and structure refinement for **Fe_{MeCN}**.

CCDC No.	2109422	
Identification code	CEC0024	
Chemical formula	$C_{49}H_{42}F_6FeN_6O_6P_2S_2$	
Formula weight	1106.79 g/mol	
Temperature	100(2) K	
Wavelength	0.71073 Å	
Crystal system	monoclinic	
Space group	C1c1	
Unit cell dimensions	a = 12.1381(4) Å	α = 90°
	b = 21.3273(7) Å	β = 104.6460(10)°
	c = 20.1399(7) Å	γ = 90°
Volume	5044.3(3) $Å^3$	
Z	4	
Density (calculated)	1.457 g/cm^3	
Absorption coefficient	0.522 mm^{-1}	
F(000)	2272	
Crystal size	0.106 x 0.237 x 0.258 mm	
Theta range for data collection	1.91 to 37.50°	
Index ranges	-20<=h<=20, -36<=k<=36, -34<=l<=34	
Reflections collected	162253	
Independent reflections	26480 [R(int) = 0.0482]	
Coverage of independent reflections	100.0%	
Absorption correction	Multi-Scan	
Max. and min. transmission	0.9470 and 0.8770	
Refinement method	Full-matrix least-squares on F^2	
Data / restraints / parameters	26480 / 2 / 652	
Goodness-of-fit on F^2	1.047	
Final R indices	R_1 = 0.0255, wR_2 = 0.0621	
R indices (all data)	R_1 = 0.0269, wR_2 = 0.0626	
Largest diff. peak and hole	0.518 and -0.357 e.$Å^{-3}$	

Table S6. Crystal data and structure refinement for **Co_{Cl}**.

CCDC No.	2109429	
Identification code	CEC0039	
Empirical formula	$C_{49}H_{49}Cl_2CoN_3O_2P_2$	
Formula weight	903.68	
Temperature	100(2) K	
Wavelength	0.71073 Å	
Crystal system	Monoclinic	
Space group	$P2_1/c$; No. 14	
Unit cell dimensions	a = 8.8971(4) Å	α = 90°.
	b = 27.2678(13) Å	β = 90.045(2)°.
	c = 17.7781(8) Å	γ = 90°.
Volume	4313.0(3) $Å^3$	
Z	4	
Density (calculated)	1.392 Mg/m^3	
Absorption coefficient	0.640 mm^{-1}	
F(000)	1884	
Crystal size	0.212 x 0.121 x 0.013 mm^3	
Theta range for data collection	1.882 to 34.999°.	
Index ranges	-14<=h<=14, -44<=k<=44, -28<=l<=27	
Reflections collected	179352	
Independent reflections	18731 [R(int) = 0.0576]	
Completeness to theta = 25.242°	99.8 %	
Absorption correction	Semi-empirical from equivalents	
Max. and min. transmission	0.9282 and 0.8728	
Refinement method	Full-matrix least-squares on F^2	
Data / restraints / parameters	18731 / 60 / 548	
Goodness-of-fit on F^2	1.109	
Final R indices [I>2sigma(I)]	R_1 = 0.0439, wR_2 = 0.0972	
R indices (all data)	R_1 = 0.0545, wR_2 = 0.1014	
Extinction coefficient	n/a	
Largest diff. peak and hole	1.006 and -0.531 e.$Å^{-3}$	

Table S7. Crystal data and structure refinement for **Co$_{Cl}$** obtained from a MeCN/Et_2O solution.

CCDC No.	2109427	
Identification code	CEC0042	
Empirical formula	$C_{43}H_{36}Cl_2CoN_4P_2$	
Formula weight	800.53	
Temperature	100(2) K	
Wavelength	0.71073 Å	
Crystal system	Monoclinic	
Space group	P 2_1/n ; No. 14	
Unit cell dimensions	a = 14.3364(4) Å	α = 90°.
	b = 18.6529(5) Å	β = 108.6880(10)°.
	c = 14.7075(4) Å	γ = 90°.
Volume	3725.65(18) $Å^3$	
Z	4	
Density (calculated)	1.427 Mg/m^3	
Absorption coefficient	0.728 mm^{-1}	
F(000)	1652	
Crystal size	0.080 x 0.060 x 0.035 mm^3	
Theta range for data collection	1.82 to 32.97°.	
Index ranges	-21<=h<=21, -28<=k<=28, -22<=l<=22	
Reflections collected	141027	
Independent reflections	13977 [R(int) = 0.0824]	
Completeness to theta = 25.24°	99.8 %	
Absorption correction	Semi-empirical from equivalents	
Max. and min. transmission	0.97 and 0.82	
Refinement method	Full-matrix least-squares on F^2	
Data / restraints / parameters	13977 / 0 / 470	
Goodness-of-fit on F^2	1.079	
Final R indices [I>2sigma(I)]	R_1 = 0.0408, wR_2 = 0.0835	
R indices (all data)	R_1 = 0.0531, wR_2 = 0.0880	
Largest diff. peak and hole	0.571 and -0.511 e.$Å^{-3}$	

Table S8. Crystal data and structure refinement for **Ni$_{Cl}$**.

CCDC No.	2109423	
Identification code	CEC0032	
Empirical formula	$C_{42}H_{35}Cl_4N_3NiP_2$	
Formula weight	844.18	
Temperature	100(2) K	
Wavelength	0.71073 Å	
Crystal system	Monoclinic	
Space group	$P2_1/c$; No. 14	
Unit cell dimensions	a = 21.9733(9) Å	α = 90°.
	b = 9.2323(3) Å	β = 112.0040(10)°.
	c = 20.2815(8) Å	γ = 90°.
Volume	3814.7(3) Å^3	
Z	4	
Density (calculated)	1.470 Mg/m^3	
Absorption coefficient	0.909 mm^{-1}	
F(000)	1736	
Crystal size	0.11 x 0.07 x 0.05 mm^3	
Theta range for data collection	1.999 to 36.368°.	
Index ranges	-36<=h<=36, -15<=k<=15, -33<=l<=33	
Reflections collected	197776	
Independent reflections	18536 [R(int) = 0.0733]	
Completeness to theta = 25.242°	100.0 %	
Absorption correction	Semi-empirical from equivalents	
Max. and min. transmission	0.8625 and 0.8073	
Refinement method	Full-matrix least-squares on F^2	
Data / restraints / parameters	18536 / 0 / 469	
Goodness-of-fit on F^2	1.093	
Final R indices [I>2sigma(I)]	R_1 = 0.0458, wR_2 = 0.0936	
R indices (all data)	R_1 = 0.0578, wR_2 = 0.0980	
Extinction coefficient	n/a	
Largest diff. peak and hole	0.826 and -0.832 e.Å^{-3}	

Table S9. Crystal data and structure refinement for **Ni_{Cl}** obtained from a MeCN/Et_2O solution.

CCDC No.	2109430	
Identification code	CEC0043	
Empirical formula	$C_{43}H_{36}Cl_2N_4NiP_2$	
Formula weight	800.31	
Temperature	100(2) K	
Wavelength	0.71073 Å	
Crystal system	Monoclinic	
Space group	$P2_1/n$; No.14	
Unit cell dimensions	a = 14.2542(7) Å	α = 90°.
	b = 18.8775(9) Å	β = 108.888(2)°.
	c = 14.6589(7) Å	γ = 90°.
Volume	3732.1(3) $Å^3$	
Z	4	
Density (calculated)	1.424 Mg/m^3	
Absorption coefficient	0.787 mm^{-1}	
F(000)	1656	
Crystal size	0.228 x 0.106 x 0.058 mm^3	
Theta range for data collection	1.86 to 36.33°.	
Index ranges	-23<=h<=23, -31<=k<=31, -24<=l<=24	
Reflections collected	203192	
Independent reflections	18091 [R(int) = 0.0582]	
Completeness to theta = 25.24°	99.9 %	
Absorption correction	Semi-empirical from equivalents	
Max. and min. transmission	0.96 and 0.89	
Refinement method	Full-matrix least-squares on F^2	
Data / restraints / parameters	18091 / 0 / 470	
Goodness-of-fit on F^2	1.154	
Final R indices [I>2sigma(I)]	R_1 = 0.0421, wR_2 = 0.0892	
R indices (all data)	R_1 = 0.0509, wR_2 = 0.0924	
Largest diff. peak and hole	0.662 and -0.422 e.$Å^{-3}$	

Table S10. Crystal data and structure refinement for **Cu_{Cl}**.

CCDC No.	2109432	
Identification code	CEC0050	
Empirical formula	$C_{42}H_{35}Cl_3CuN_3P_2$	
Formula weight	813.56	
Temperature	100(2) K	
Wavelength	0.71073 Å	
Crystal system	Monoclinic	
Space group	$P2_1/c$; No.14	
Unit cell dimensions	a = 15.0181(6) Å	α = 90°.
	b = 13.7846(6) Å	β = 93.208(2)°.
	c = 18.3831(8) Å	γ = 90°.
Volume	3799.7(3) $Å^3$	
Z	4	
Density (calculated)	1.422 Mg/m^3	
Absorption coefficient	0.905 mm^{-1}	
F(000)	1672	
Crystal size	0.130 x 0.060 x 0.050 mm^3	
Theta range for data collection	1.848 to 37.500°.	
Index ranges	-25<=h<=25, -23<=k<=23, -31<=l<=31	
Reflections collected	310452	
Independent reflections	19998 [R(int) = 0.0517]	
Completeness to theta = 25.242°	100.0 %	
Absorption correction	Semi-empirical from equivalents	
Max. and min. transmission	0.96 and 0.92	
Refinement method	Full-matrix least-squares on F^2	
Data / restraints / parameters	19998 / 0 / 460	
Goodness-of-fit on F^2	1.085	
Final R indices [I>2sigma(I)]	R_1 = 0.0403, wR_2 = 0.0903	
R indices (all data)	R_1 = 0.0470, wR_2 = 0.0933	
Extinction coefficient	n/a	
Largest diff. peak and hole	1.009 and -0.935 e.$Å^{-3}$	

Table S11. Crystal data and structure refinement for **Cu_I**.

CCDC No.	2109431	
Identification code	CEC0034	
Empirical formula	$C_{45}H_{41}CuIN_3OP_2$	
Formula weight	892.19	
Temperature	100(2) K	
Wavelength	0.71073 Å	
Crystal system	Monoclinic	
Space group	P21/n; No. 14	
Unit cell dimensions	a = 11.4338(6) Å	α = 90°.
	b = 22.8575(12) Å	β = 95.401(2)°.
	c = 15.3445(8) Å	γ = 90°.
Volume	3992.5(4) $Å^3$	
Z	4	
Density (calculated)	1.484 Mg/m^3	
Absorption coefficient	1.439 mm^{-1}	
F(000)	1808	
Crystal size	0.200 x 0.120 x 0.060 mm^3	
Theta range for data collection	2.00 to 40.00°.	
Index ranges	-20<=h<=20, -41<=k<=41, -27<=l<=27	
Reflections collected	468847	
Independent reflections	24736 [R(int) = 0.0452]	
Completeness to theta = 25.24°	100.0 %	
Absorption correction	Semi-empirical from equivalents	
Max. and min. transmission	0.92 and 0.82	
Refinement method	Full-matrix least-squares on F^2	
Data / restraints / parameters	24736 / 0 / 478	
Goodness-of-fit on F^2	1.133	
Final R indices [I>2sigma(I)]	R_1 = 0.0267, wR_2 = 0.0600	
R indices (all data)	R_1 = 0.0299, wR_2 = 0.0612	
Largest diff. peak and hole	1.695 and -0.901 e.$Å^{-3}$	

Table S12. Crystal data and structure refinement for **Zn_{Cl}**.

CCDC No.	2109426	
Identification code	CEC0037	
Empirical formula	$C_{45}H_{41}Cl_2N_3OP_2Zn$	
Formula weight	838.02	
Temperature	100(2) K	
Wavelength	0.71073 Å	
Crystal system	Triclinic	
Space group	P-1	
Unit cell dimensions	a = 10.9843(7) Å	α = 86.877(2)°.
	b = 12.8901(8) Å	β = 73.228(2)°.
	c = 15.0226(10) Å	γ = 84.511(2)°.
Volume	2026.4(2) $Å^3$	
Z	2	
Density (calculated)	1.373 Mg/m^3	
Absorption coefficient	0.856 mm^{-1}	
F(000)	868	
Crystal size	0.242 x 0.201 x 0.086 mm^3	
Theta range for data collection	1.94 to 33.16°.	
Index ranges	-16<=h<=16, -19<=k<=19, -23<=l<=23	
Reflections collected	141741	
Independent reflections	15448 [R(int) = 0.0381]	
Completeness to theta = 25.24°	99.8 %	
Absorption correction	Semi-empirical from equivalents	
Max. and min. transmission	0.93 and 0.85	
Refinement method	Full-matrix least-squares on F^2	
Data / restraints / parameters	15448 / 0 / 487	
Goodness-of-fit on F^2	1.054	
Final R indices [I>2sigma(I)]	R_1 = 0.0301, wR_2 = 0.0787	
R indices (all data)	R_1 = 0.0355, wR_2 = 0.0829	
Largest diff. peak and hole	0.802 and -0.410 e.$Å^{-3}$	

Table S13. Crystal data and structure refinement for **Zn_{OTf}**.

CCDC No.	2109420	
Identification code	CEC0006_2	
Empirical formula	$C_{51}H_{49}F_6N_3O_8P_2S_2Zn$	
Formula weight	1137.36	
Temperature	100(2) K	
Wavelength	0.71073 Å	
Crystal system	Monoclinic	
Space group	$P2_1$, No. 4	
Unit cell dimensions	a = 9.7544(6) Å	α = 90°.
	b = 13.6249(8) Å	β = 96.592(2)°.
	c = 19.3929(11) Å	γ = 90°.
Volume	2560.3(3) $Å^3$	
Z	2	
Density (calculated)	1.475 Mg/m^3	
Absorption coefficient	0.702 mm^{-1}	
F(000)	1172	
Crystal size	0.220 x 0.130 x 0.100 mm^3	
Theta range for data collection	2.114 to 33.658°.	
Index ranges	-12<=h<=15, -21<=k<=21, -30<=l<=30	
Reflections collected	114914	
Independent reflections	20199 [R(int) = 0.0450]	
Completeness to theta = 33.658°	99.6 %	
Absorption correction	Semi-empirical from equivalents	
Max. and min. transmission	0.93 and 0.85	
Refinement method	Full-matrix least-squares on F^2	
Data / restraints / parameters	20199 / 61 / 659	
Goodness-of-fit on F^2	1.141	
Final R indices [I>2sigma(I)]	R_1 = 0.0457, wR_2 = 0.0982	
R indices (all data)	R_1 = 0.0509, wR_2 = 0.0999	
Absolute structure parameter	0.050(8)	
Extinction coefficient	n/a	
Largest diff. peak and hole	1.166 and -0.771 e.$Å^{-3}$	

1.6.3. Calculation of Geometry Indices

Table S14. Angles $\boldsymbol{\theta_{apical}}$ between the apical chlorido ligand Cl_{ap} and the ligands in the basal plane of $\mathbf{M_{Cl}}$ complexes (M = Fe, Co, Ni).

	θ_{apical}(Cl_{ap}-M-Cl_2) (°)	θ_{apical}(Cl_{ap}-M-N) (°)	θ_{apical}(Cl_{ap}-M-P_1) (°)	θ_{apical}(Cl_{ap}-M-P_2) (°)
**Fe_{Cl}**	108	98	117	103
**Co_{Cl}**	105	91	96	93
**Ni_{Cl}**	102	88	95	108

Table S15. Two greatest valence angles ($\boldsymbol{\beta} > \boldsymbol{\alpha}$) of the coordination center for tetra- and penta-coordinated complexes investigated in this study.

	α (°)	β (°)
**Fe_{Cl}**	130.41(2)	153.00(3)
**Co_{Cl}**	164.14(4)	166.28(2)
**Ni_{Cl}**	155.51(2)	169.44(4)
**Cu_{Cl}**	114.37(2)	135.98(2)
**Cu_{I}**	118.90(2)	133.22(2)
**Zn_{Cl}**	112.60(1)	113.82(2)
**Zn_{OTf}**	129.09(3)	178.10(9)

The geometry indices τ_4 of tetra-coordinated compounds **Cu_{Cl}**, **Cu_{I}** and **Zn_{Cl}** were calculated using equation (2) as reported by the Houser group[2]

$$\tau_4 = \frac{360° - (\alpha + \beta)}{360° - 2\theta} \tag{2}$$

α and β being the two greatest valence angles ($\beta > \alpha$) of the coordination center (Table S15) and $\theta \approx 109.5°$.

The geometry indices τ_5 of pentacoordinated compounds **Fe_{Cl}**, **Co_{Cl}**, **Ni_{Cl}** and **Zn_{OTf}** were calculated using equation (3) as reported by Addison et al.[3]

$$\tau_5 = \frac{\beta - \alpha}{60°} \tag{3}$$

2. Electrochemical Analysis

2.1. Cyclic Voltammograms

2.1.1. Ligand and Complexes in Extended Potential Windows

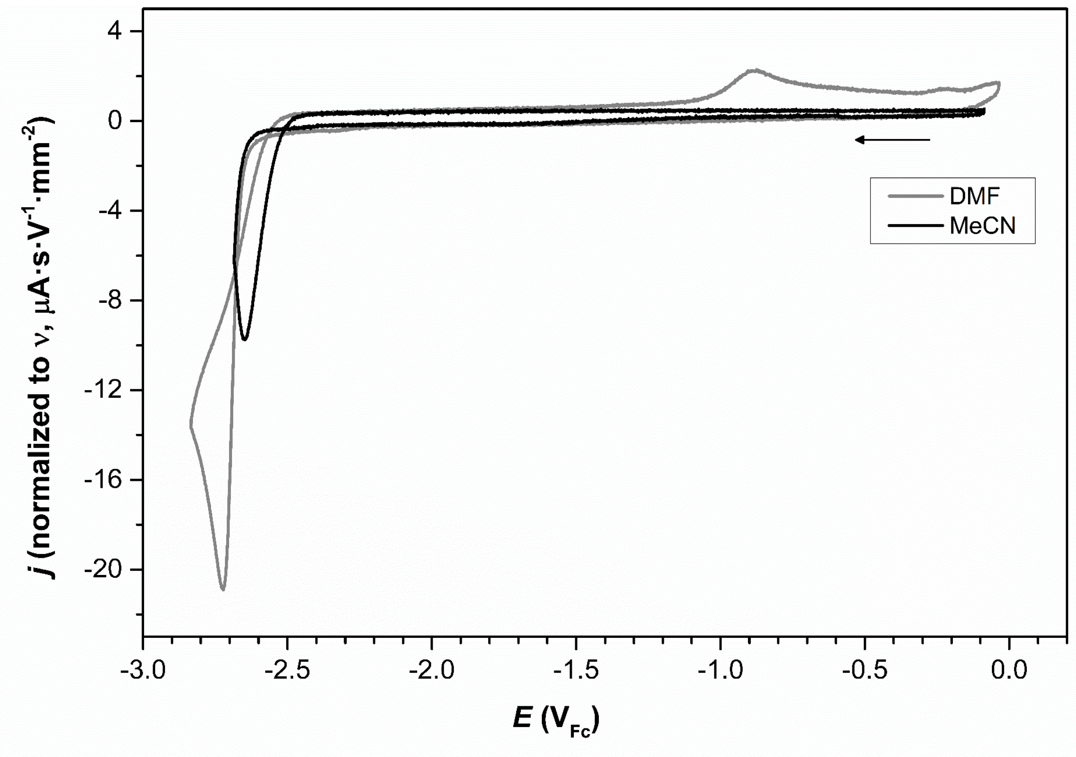

Figure S40. Cyclic voltammograms of **L** under argon atmosphere in MeCN and DMF ([**L**] = 1 mM, 0.1 M $^{n}Bu_4NPF_6$, glassy carbon working electrode, ν = 100 mV·s^{-1}).

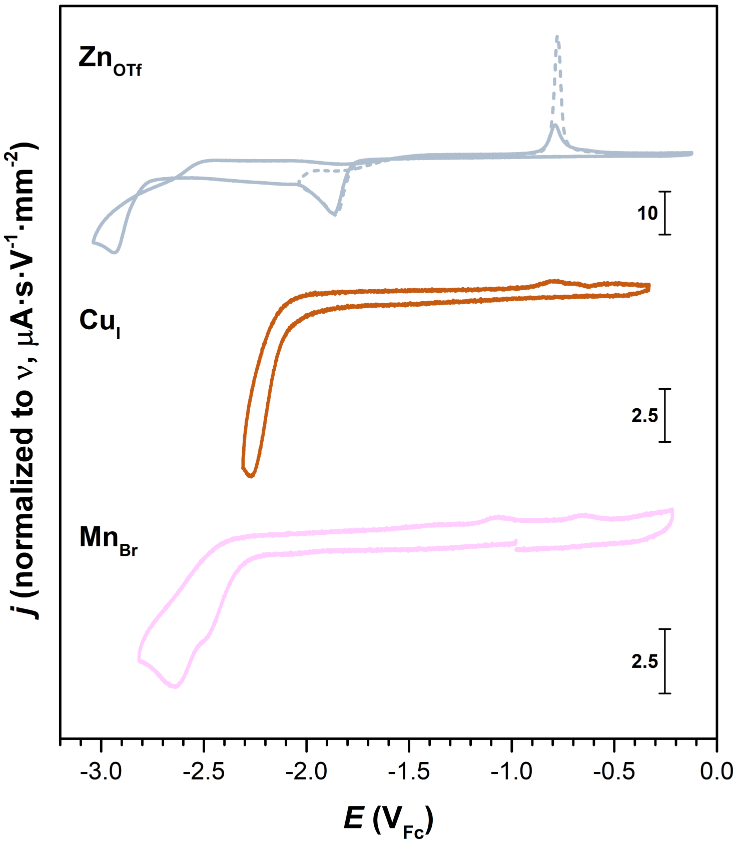

Figure S41. Cyclic voltammograms of **Mn**$_{Br}$, **Cu**$_{I}$ and **Zn**$_{OTf}$ under argon atmosphere ([**M**$_{X}$] = 1 mM, MeCN (Cu, Zn)/DMF (Mn), 0.1 M ^{n}Bu$_4$NPF$_6$, glassy carbon working electrode, ν = 100 mV·s^{-1}).

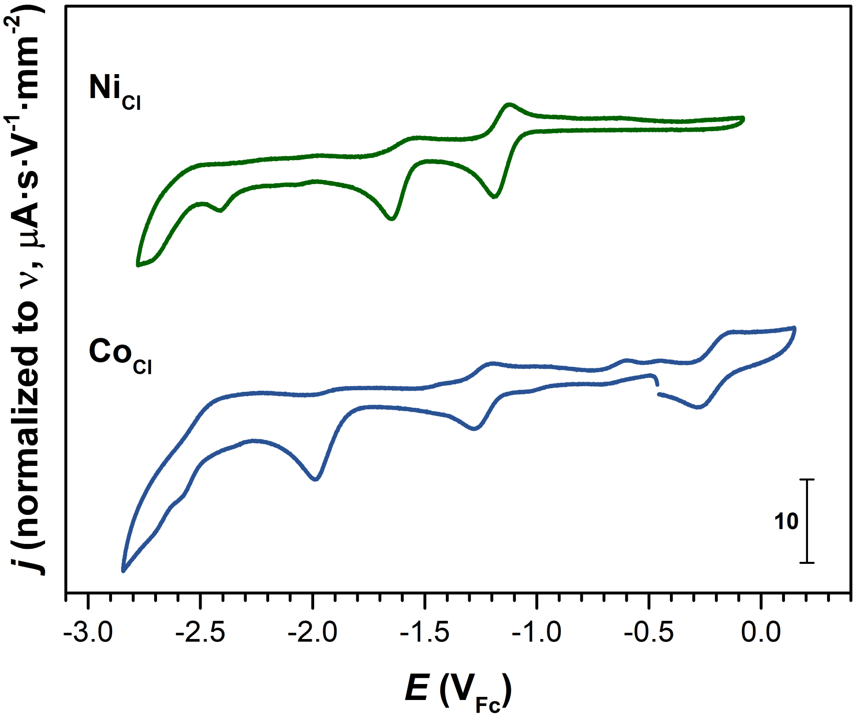

Figure S42. Cyclic voltammograms of **M**$_{Cl}$ (M = Co, Ni) under argon atmosphere ([**M**$_{X}$] = 1 mM, MeCN, 0.1 M ^{n}Bu$_4$NPF$_6$, glassy carbon working electrode, ν = 100 mV·s^{-1}).

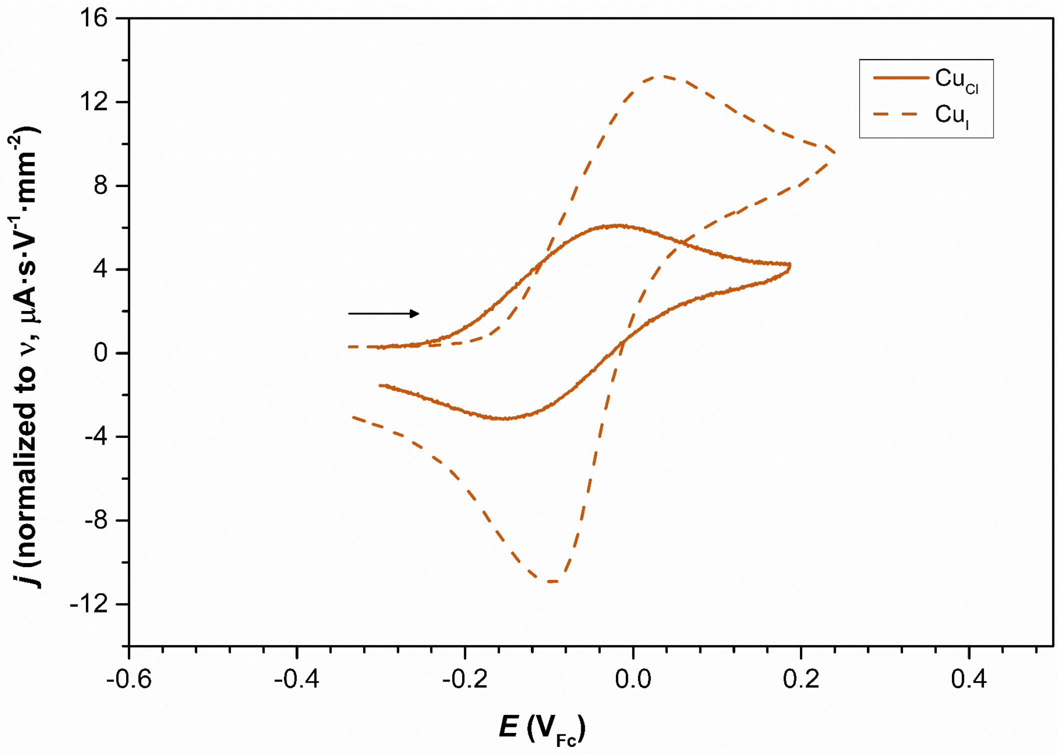

Figure S43. $Cu^{II/I}$ wave in cyclic voltammograms of **Cu**X (X = Cl, I) under argon atmosphere ([**Cu**X] = 1 mM, MeCN, 0.1 M $^{n}Bu_4NPF_6$, glassy carbon working electrode, ν = 100 mV·s^{-1}).

2.1.2. [FeL(MeCN)$_3$](OTf)$_2$

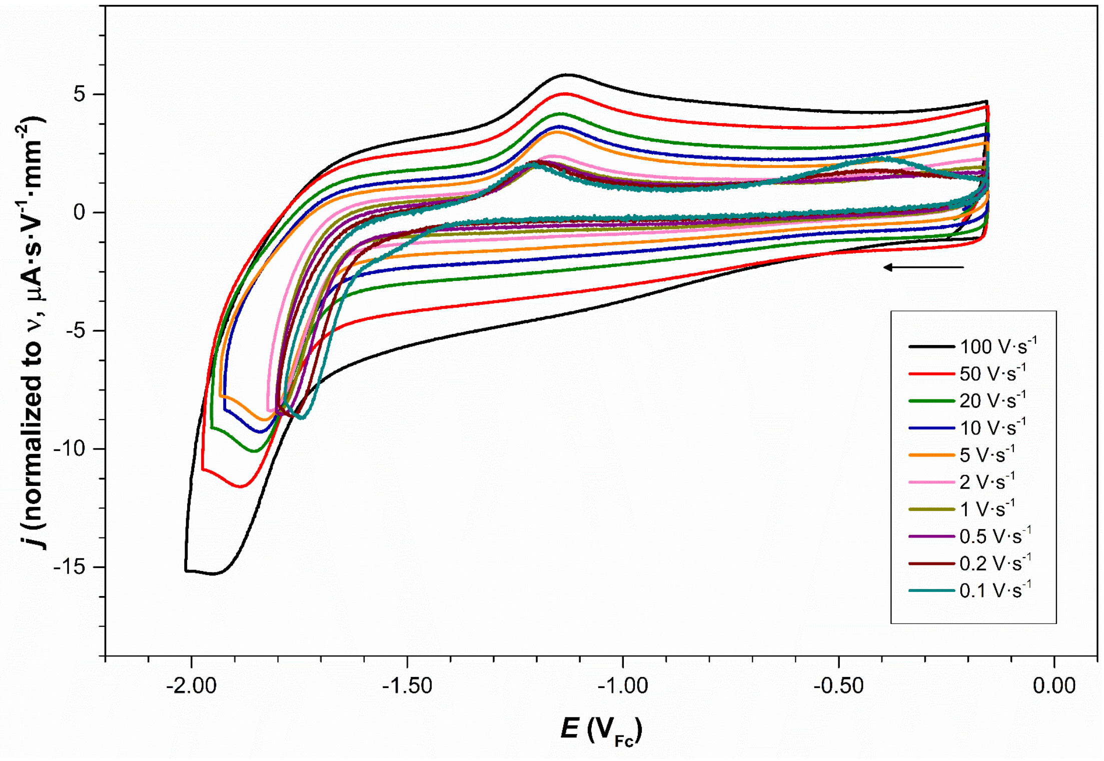

Figure S44. Cyclic voltammograms of the M$^{II/I}$ wave of **Fe$_{MeCN}$** under argon atmosphere at varying scan rates ([**Fe$_{MeCN}$**] = 1 mM, MeCN, 0.1 M ^{n}Bu$_4$NPF$_6$, glassy carbon working electrode).

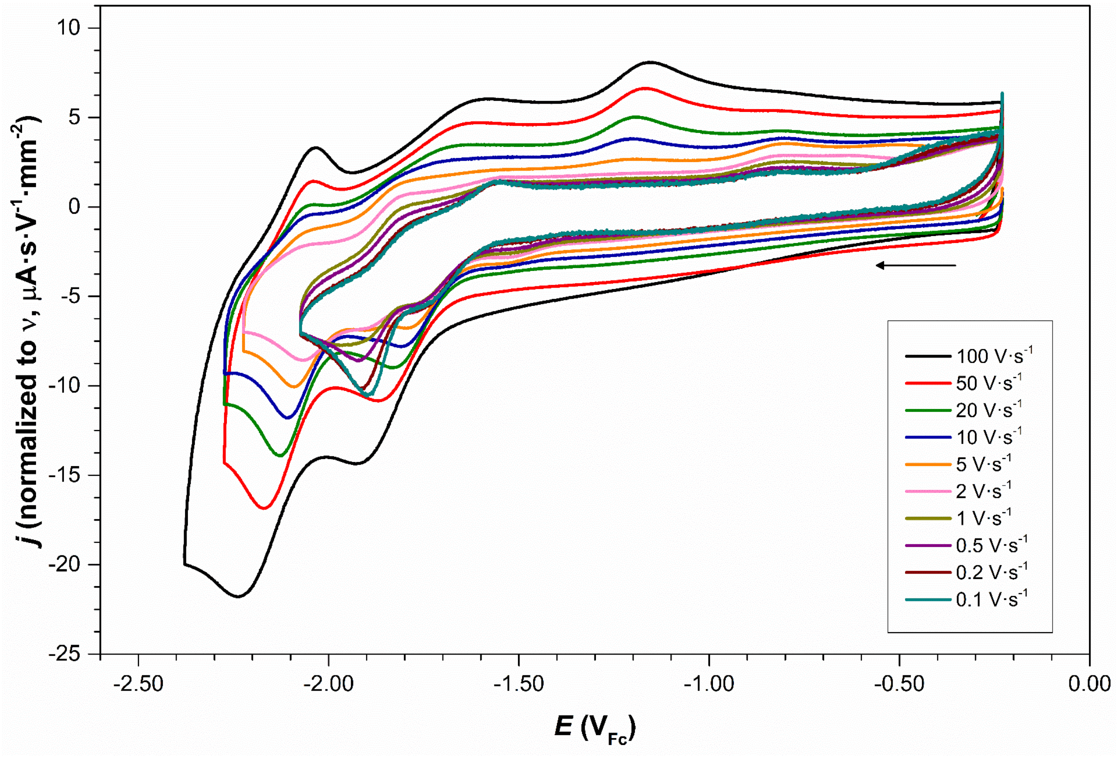

Figure S45. Cyclic voltammograms of **Fe$_{MeCN}$** under argon atmosphere at varying scan rates ([**Fe$_{MeCN}$**] = 1 mM, MeCN, 0.1 M ^{n}Bu$_4$NPF$_6$, glassy carbon working electrode).

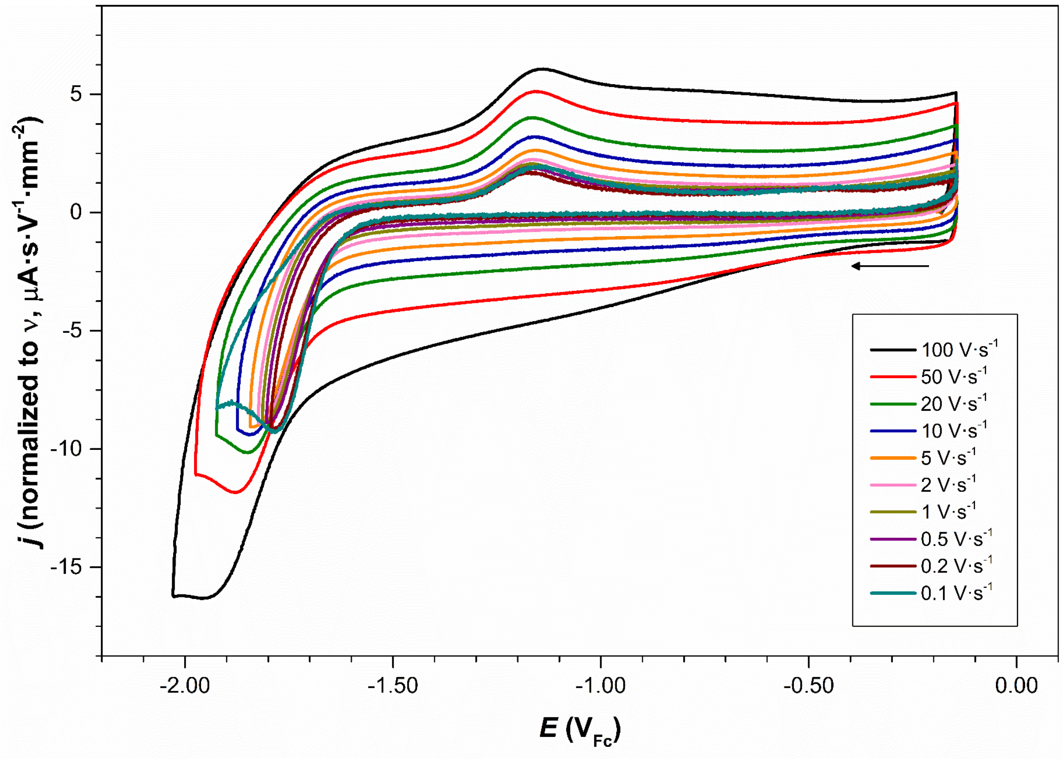

Figure S46. Cyclic voltammograms of the $M^{II/I}$ wave of **Fe$_{MeCN}$** under CO_2 atmosphere at varying scan rates ([**Fe$_{MeCN}$**] = 1 mM, MeCN, 0.1 M ^{n}Bu$_4$NPF$_6$, glassy carbon working electrode).

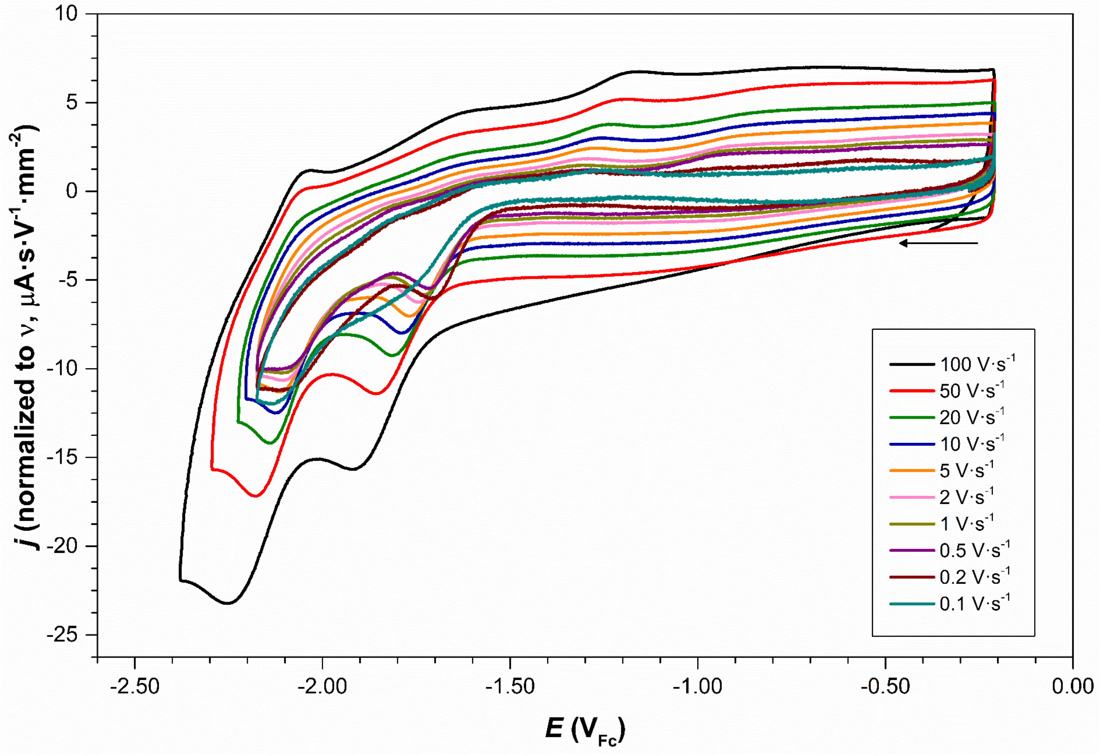

Figure S47. Cyclic voltammograms of **Fe$_{MeCN}$** under CO_2 atmosphere at varying scan rates ([**Fe$_{MeCN}$**] = 1 mM, MeCN, 0.1 M ^{n}Bu$_4$NPF$_6$, glassy carbon working electrode).

2.1.3. [CoLCl$_2$]

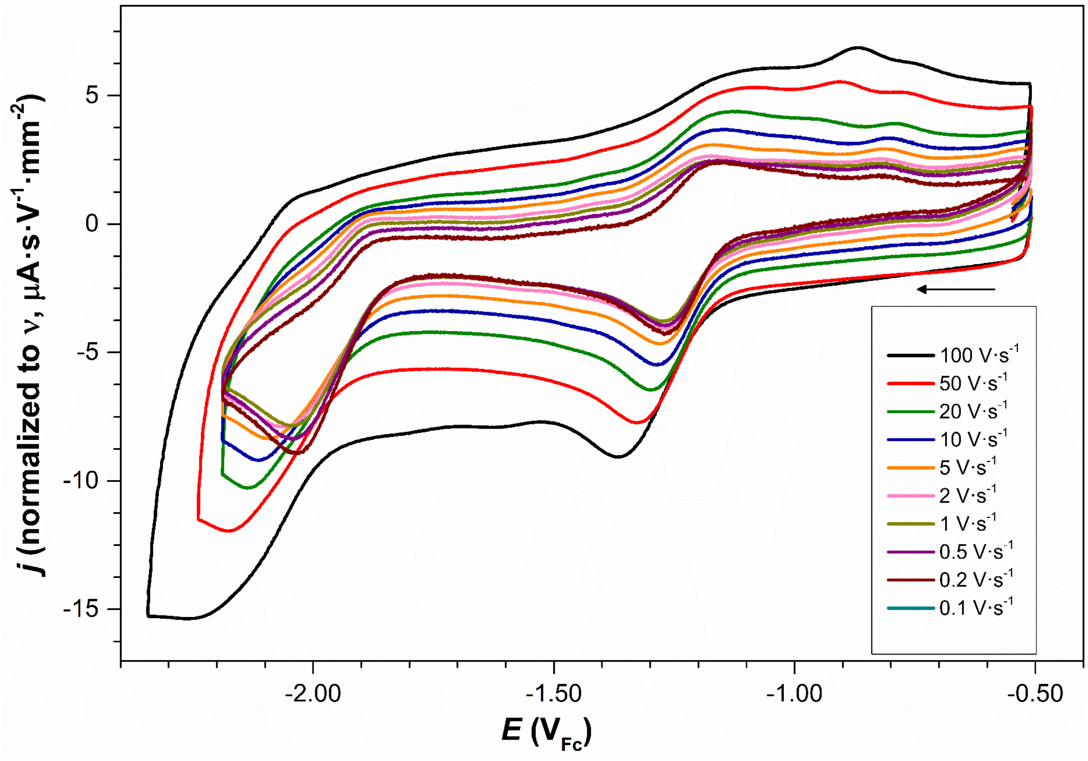

Figure S48. Cyclic voltammograms of **Co$_{Cl}$** under Ar atmosphere at varying scan rates ([**Co$_{Cl}$**] = 1 mM, MeCN, 0.1 M ^{n}Bu$_4$NPF$_6$, glassy carbon working electrode).

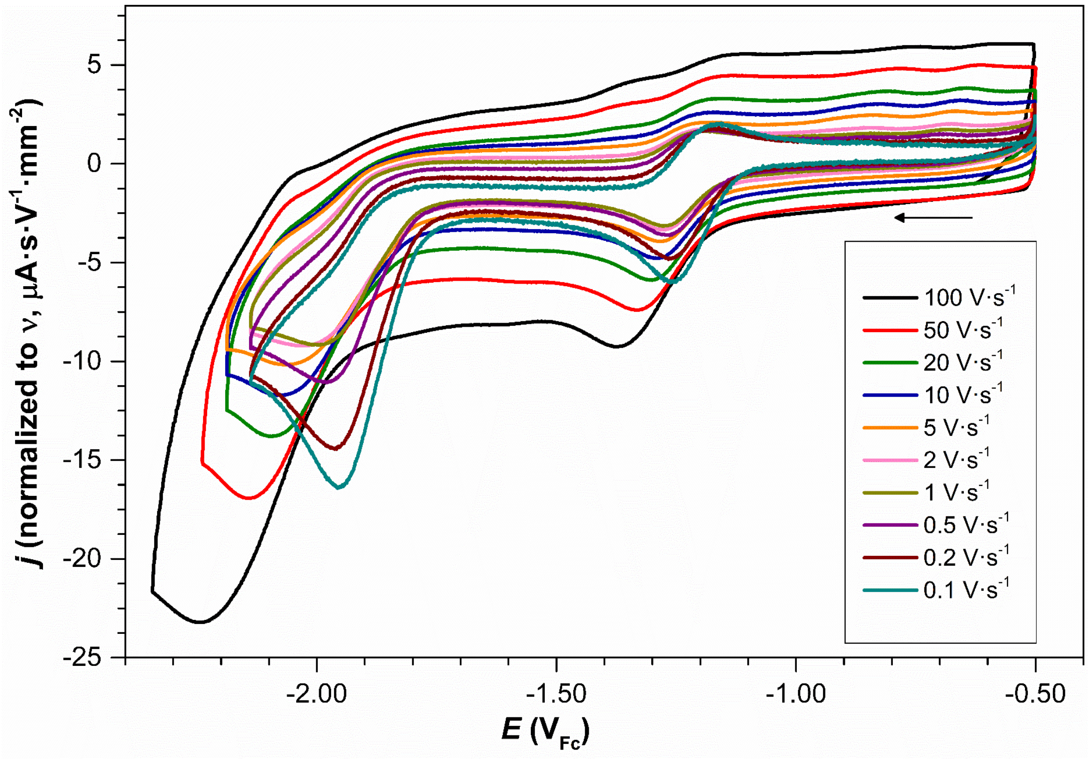

Figure S49. Cyclic voltammograms of **Co$_{Cl}$** under CO_2 atmosphere at varying scan rates ([**Co$_{Cl}$**] = 1 mM, MeCN, 0.1 M ^{n}Bu$_4$NPF$_6$, glassy carbon working electrode).

2.1.4. [$NiLCl_2$]

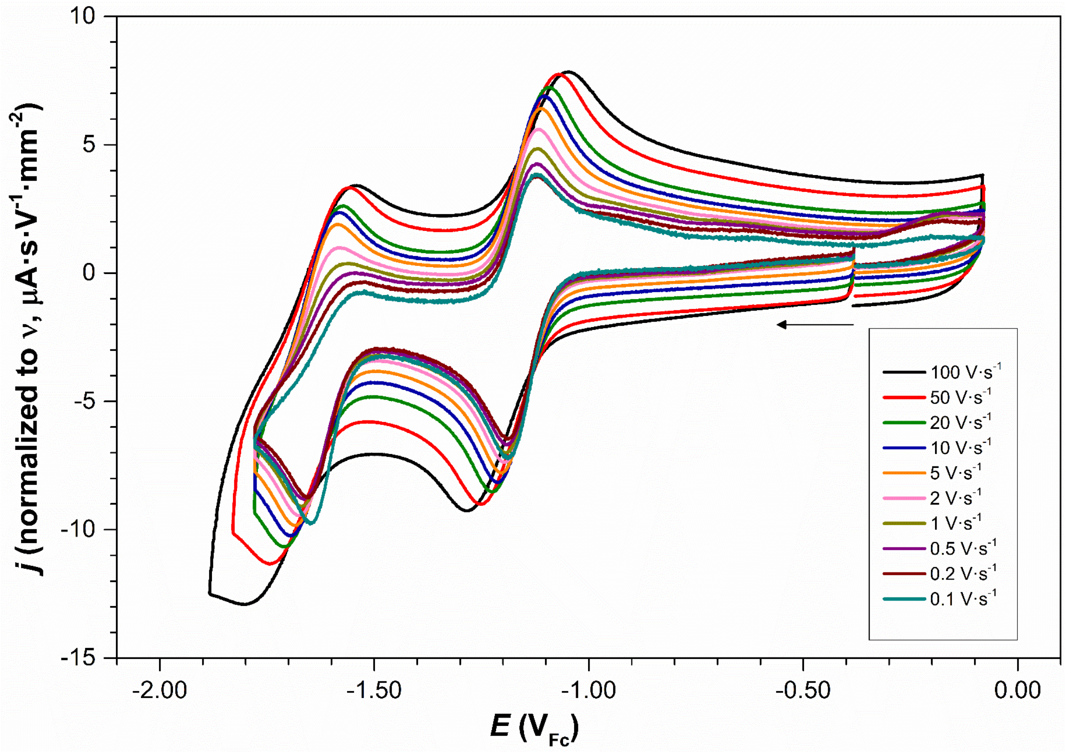

Figure S50. Cyclic voltammograms of **Ni**$_{\mathbf{Cl}}$ under Ar atmosphere at varying scan rates ([**Ni**$_{\mathbf{Cl}}$] = 1 mM, MeCN, 0.1 M $^{n}Bu_4NPF_6$, glassy carbon working electrode).

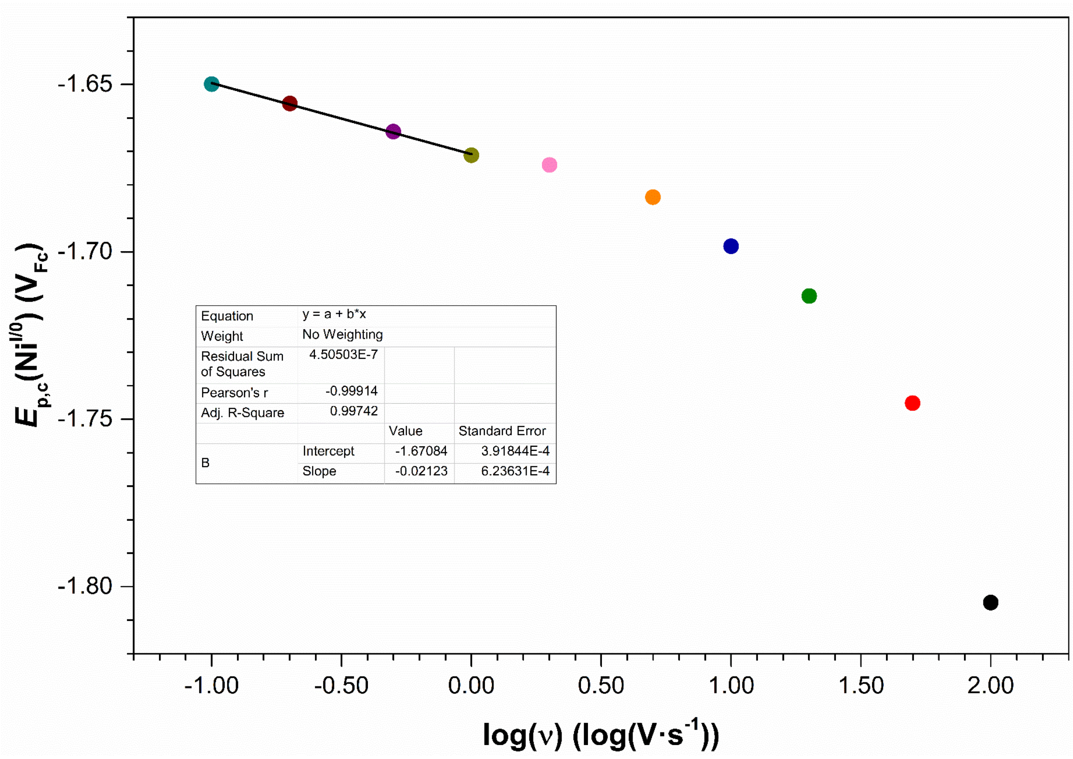

Figure S51. $E_{p,c}(Ni^{I/0})$ vs. log(ν) for **Ni**$_{\mathbf{Cl}}$ under Ar atmosphere ([**Ni**$_{\mathbf{Cl}}$] = 1 mM, MeCN, 0.1 M $^{n}Bu_4NPF_6$, glassy carbon working electrode).

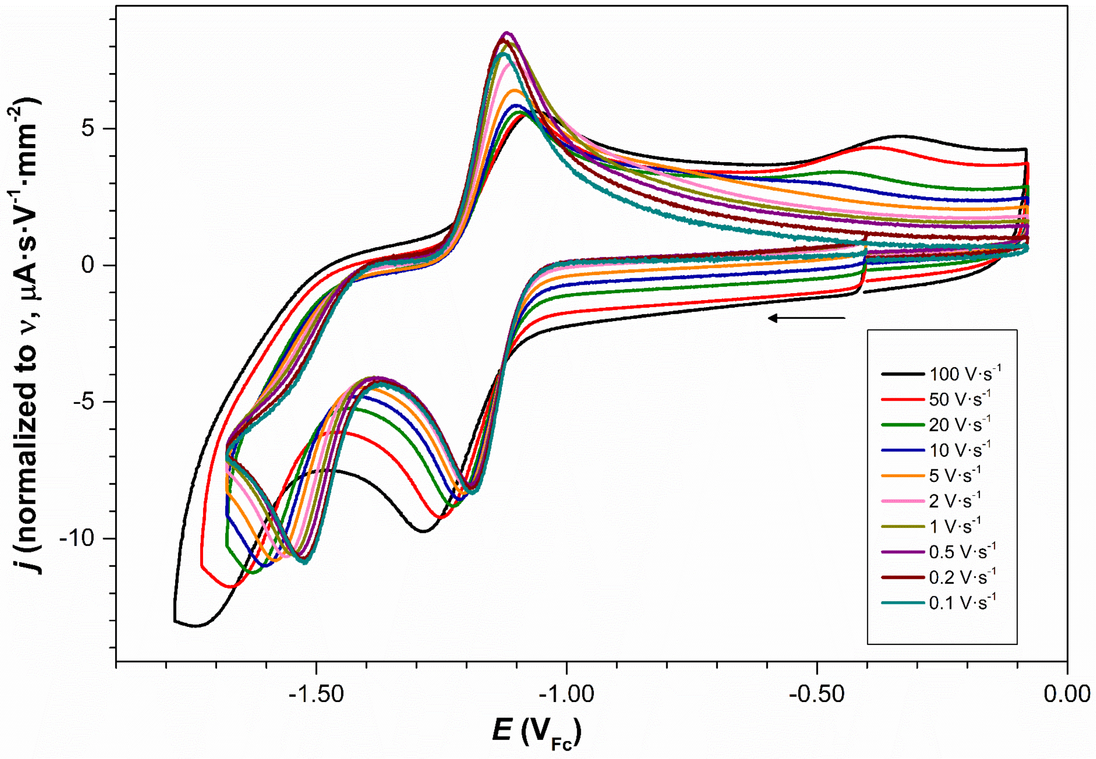

Figure S52. Cyclic voltammograms of **Ni$_{Cl}$** under CO_2 atmosphere at varying scan rates ([**Ni$_{Cl}$**] = 1 mM, MeCN, 0.1 M ^{n}Bu$_4$NPF$_6$, glassy carbon working electrode).

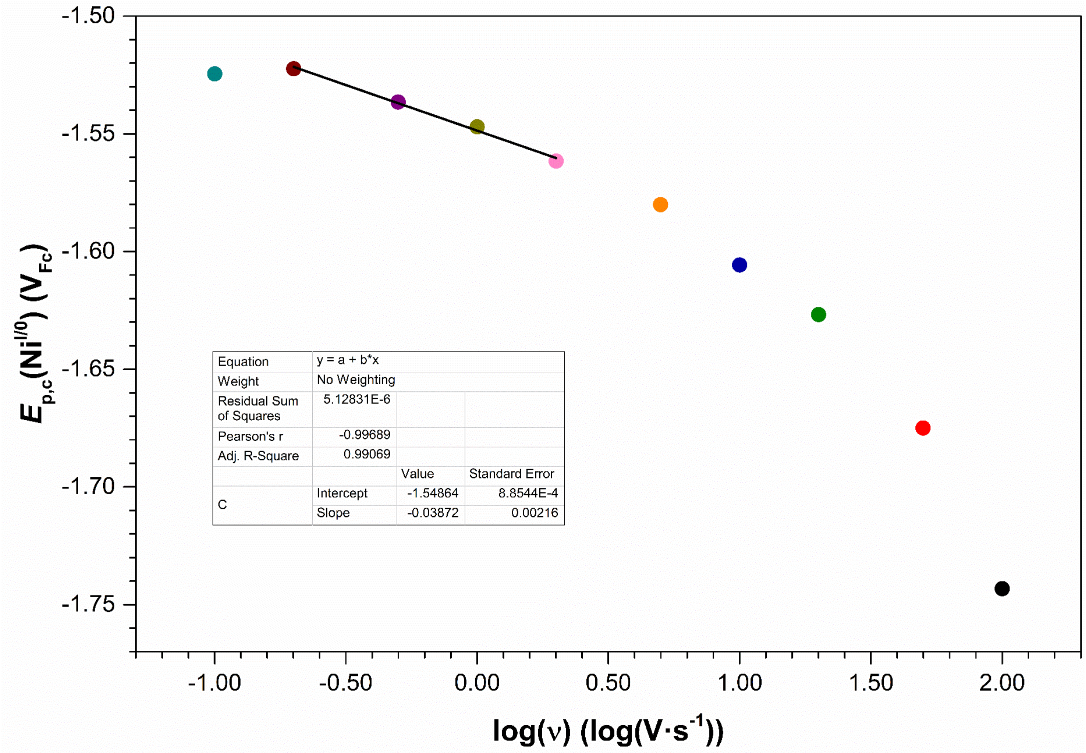

Figure S53. $E_{p,c}$(Ni$^{I/0}$) vs. log(ν) for **Ni$_{Cl}$** under CO_2 atmosphere ([**Ni$_{Cl}$**] = 1 mM, MeCN, 0.1 M ^{n}Bu$_4$NPF$_6$, glassy carbon working electrode).

2.2. Calculations of Rate Constants for Follow-Up Reactions

The forward rate constant k_f of a chemical event (C) following up an electrochemical step (E) corresponding to a redox couple of standard potential E^0 is related under *pure kinetic* conditions (EC mechanism in the *KP* zone) to the voltamperometric peak potential E_p by:[4]

$$E_p = E^0 - 0.78\frac{RT}{F} + \frac{RT}{2F}\ln\left(\frac{RT\,k_f}{F\,\nu}\right) \tag{4}$$

where ν is the voltamperometric scan rate, R the ideal gas constant, *T* the temperature and F the Faraday constant.

Noting *a* the intercept of the linear fit of E_p = f(log(ν)), rearrangement gives:

$$k_f = \frac{F}{RT}\,exp\left(1.56 + \frac{2F}{RT}(a - E^0)\right) \tag{5}$$

Under Ar, the cathodic peak potential of the $Ni^{I/0}$ couple of **Ni_{Cl}** decays logarithmically of scan rate (*KP* zone) by –21.2 mV·dec^{-1} (Figure S51; theoretical value expected at –29.6 mV·dec^{-1}) and with an intercept of *a* = –1.67 V_{Fc}. From this data and prior knowledge of E^0($Ni^{I/0}$) = –1.59 V_{Fc} (see main text), the apparent forward rate constant of the follow-up chemical step was estimated at $k_{f,app}$(Ar) = 3.6·10^{-1} s^{-1}.

Under CO_2, the same analysis at the $Ni^{I/0}$ wave gives *a* = –1.55 V_{Fc} for $E_{p,c}$ = f(log(ν)) (slope of 38.7 mV·dec^{-1}; Figure S53) and thus, taking E^0($Ni^{I/0}$) = –1.59 V_{Fc}, $k_{f,app}$(CO_2) = 4.2·10^3 s^{-1}.

References

(1) Kühl, O. In *Phosphorus-31 NMR Spectroscopy: A Concise Introduction for the Synthetic Organic and Organometallic Chemist*; Kühl, O., Ed.; Springer Berlin Heidelberg: Berlin, Heidelberg, 2008; pp 83-127.

(2) Yang, L.; Powell, D. R.; Houser, R. P. Structural variation in copper(i) complexes with pyridylmethylamide ligands: structural analysis with a new four-coordinate geometry index, τ4. *Dalton Trans.* **2007**, (9), 955-964.

(3) Addison, A. W.; Rao, T. N.; Reedijk, J.; van Rijn, J.; Verschoor, G. C. Synthesis, structure, and spectroscopic properties of copper(II) compounds containing nitrogen–sulphur donor ligands; the crystal and molecular structure of aqua[1,7-bis(N-methylbenzimidazol-2′-yl)-2,6-dithiaheptane]copper(II) perchlorate. *J. Chem. Soc., Dalton Trans.* **1984**, (7), 1349-1356.

(4) Savéant, J.-M.; Costentin, C. In *Elements of Molecular and Biomolecular Electrochemistry*; 2nd ed.; John Wiley & Sons Inc.: 2019; p 86.
